# New Amides and Phenylpropanoid Glucosides from the Fruits of *Piper retrofractum*

**DOI:** 10.1007/s13659-019-0208-z

**Published:** 2019-05-09

**Authors:** Rong Tang, Ya-Qiong Zhang, Dong-Bao Hu, Xue-Fei Yang, Jun Yang, Myint Myint San, Thaung Naing Oo, Yi Kong, Yue-Hu Wang

**Affiliations:** 1 Key Laboratory of Economic Plants and Biotechnology and the Yunnan Key Laboratory for Wild Plant Resources, Kunming Institute of Botany, Chinese Academy of Sciences, Kunming, 650201 People’s Republic of China; 2 School of Life Science & Technology, China Pharmaceutical University, Nanjing, 210009 People’s Republic of China; 3 University of Chinese Academy of Sciences, Beijing, 100049 People’s Republic of China; 4 School of Chemical Biology and Environment, Yuxi Normal University, Yuxi, 653100 People’s Republic of China; 5 Southeast Asia Biodiversity Research Institute, Chinese Academy of Sciences, Yezin, Nay Pyi Taw 05282 Myanmar; 6 Forest Research Institute, Yezin, Nay Pyi Taw 05282 Myanmar

**Keywords:** *Piper retrofractum*, Piperaceae, Antiplatelet, Amides, Phenylpropanoids

## Abstract

**Abstract:**

Two new amides (*E*)-*N*-cinnamoyl-2-methoxypiperidine (**1**) and (*R*)-1-(2-oxopyrrolidin-3-yl)-5,6-dihydropyridin-2(1*H*)-one (**2**), four new amide glucosides, retrofractosides A–D (**3**–**6**), and two new phenylpropanoid glucosides, retrofractosides E (**7**) and F (**8**), together with 24 known compounds (**9**–**32**) were isolated from the fruits of *Piper retrofractum*. The chemical structures of these new compounds were elucidated based on extensive spectroscopic analysis. All of these isolates (**1**–**32**) were evaluated for inhibitory activity against mouse platelet aggregation induced by the peptide AYPGKF-NH_2_. (*E*)-*N*-(Tetrahydro-2*H*-pyran-2-yl)cinnamamide (**9**) showed a weak inhibitory effect, with an inhibition ratio of 52.0% at a concentration of 150 μM.

**Graphical Abstract:**

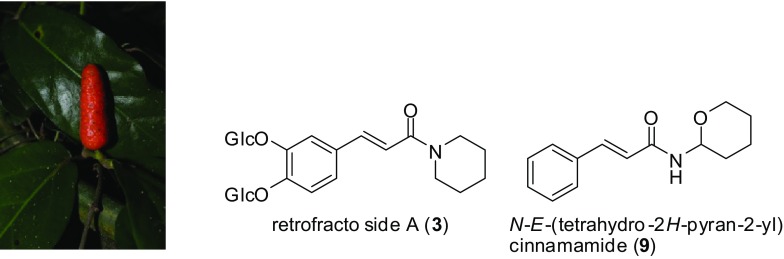

**Electronic supplementary material:**

The online version of this article (10.1007/s13659-019-0208-z) contains supplementary material, which is available to authorized users.

## Introduction

**Fig. 1:**
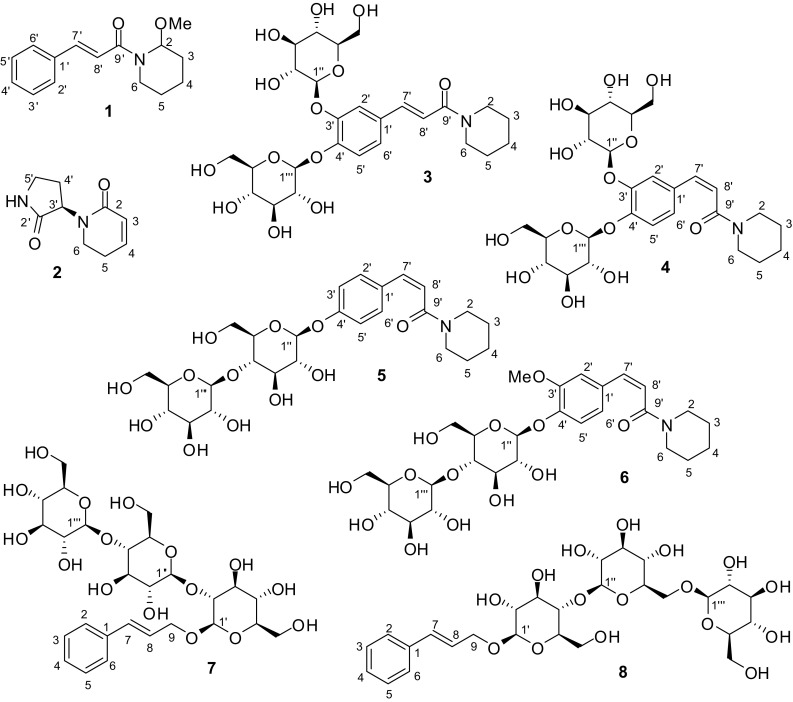
Chemical structures of new compounds (**1**–**8**) from *Piper retrofractum*

*Piper retrofractum* Vahl (Piperaceae) is primarily distributed in Southeast Asia and cultivated in Indonesia and Thailand [1]. The fruits of this plant have been used in folk medicine to treat asthma, bronchitis, dyspepsia, and sleep problems in Southeast Asia [2]. The major constituents of *P. retrofractum* are amides [3–6]. Several amides from the plant exhibit significant biological activities, such as antifungal [5], insecticidal [7], hepatoprotective [8], and gastroprotective activities [9]. *Piper* plants are rich in amides, lignans, and phenylpropanoids with antiplatelet aggregation activities. More than 50 antiplatelet compounds have been found from this genus [10–13]. In a continuing effort to search for antiplatelet compounds from *Piper* plants [11–13], the fruits of *P. retrofractum* were phytochemically studied, which led to the isolation of eight new compounds (**1**–**8**, Fig. 1). The structural elucidation of these new compounds and the results of the antiplatelet bioassays are reported here.

## Results and Discussion

### Structure Elucidation

Compound **1** was obtained as a white amorphous powder. Its molecular formula was determined to be C_15_H_19_NO_2_ by ^13^C NMR data (Table 1) and HREIMS, which revealed a molecular ion peak at *m/z* 245.1423 [M]^+^ (calcd for C_15_H_19_NO_2_, 245.1416), which implies seven degrees of unsaturation.

**Table 1:** ^1^H (800 MHz) and ^13^C NMR (201 MHz) data of **1** in CDCl_3_

No.	*δ*_H_ (*J*, Hz)	*δ*_C_
2	5.91 (br s)	79.9
	5.28 (br s)	83.9
3	1.98 (br d, 12.0), 1.63 (m)	31.2
	1.94 (br d, 13.3), 1.61 (m)	30.2
4	1.85 (2H, m), 1.61 (2H, m)	18.9
5	1.73 (m), 1.51 (m)	26.2
	1.73 (m), 1.50 (m)	25.1
6	4.49 (br d, 12.3), 2.88 (br t, 12.3)	37.1
	3.82 (br d, 12.6), 3.34 (br t, 12.6)	41.1
1′		135.4
2′,6′	7.53 (2H, br d, 7.5), 7.52 (2H, br d, 7.5)	127.94, 127.88
3′,5′	7.38 (4H, m)	129.0
4′	7.36 (2H, m)	129.8
7′	7.69 (d, 15.4), 7.68 (d, 15.4)	143.4, 143.1
8′	6.91 (d, 15.4), 6.90 (d, 15.4)	117.7
9′		166.7, 166.5
2-OMe	3.28 (3H, s)	55.1
	3.25 (3H, s)	54.4

**Fig. 2:**
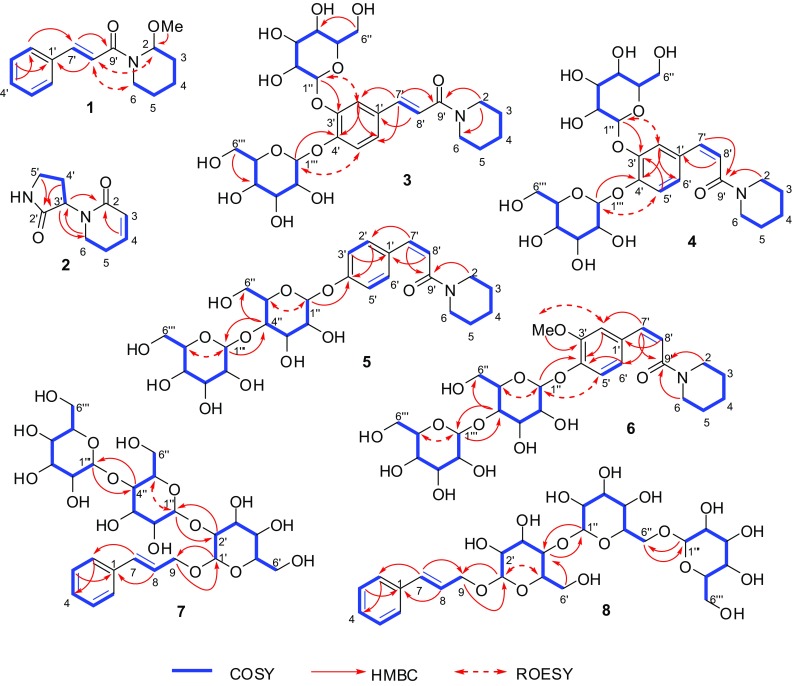
Key 2D NMR correlations of compounds **1**–**8**

The ^1^H NMR and ^13^C NMR data (Table 1) indicated the presence of an (*E*)-cinnamoyl moiety and a 2-methoxypiperidine moiety, as demonstrated by comparing the NMR data with those from (*E*)-*N*-(tetrahydro-2*H*-pyran-2-yl)cinnamamide (**9**) and *N*-benzyloxycarbonyl-2-methoxypiperidine [14]. The double bond was deduced to be an *E* configuration from the coupling constant between H-7′ and H-8′ (*J*_7′,8′_ = 15.4 Hz). Based on the HMBC correlations from OMe to C-2 (Fig. 2), the methoxy group was located at C-2. The (*E*)-cinnamoyl and 2-methoxypiperidine moieties were confirmed by the COSY and HMBC correlations (Fig. 2). Although correlations from H-2 to C-9′ and H_2_-6 to C-9′ were not observed in the HMBC spectrum, based on the molecular formula of **1** that was deduced from its HREIMS spectrum as well as the chemical shift of C-9′ indicating an amide carbonyl group, it can be concluded that these two fragments are connected through an amide bond. The ROESY correlations of H-2/H-8′ and H-6/H-8′ (Fig. 2) also support the deduction. Therefore, the planar structure of **1** was elucidated to be (*E*)-*N*-cinnamoyl-2-methoxypiperidine. It is noteworthy that NMR signals for the two rotational isomers of **1** were observed due to the hindered rotation about the amide bond [15]. No Cotton effects were observed in the ECD spectrum (data not shown). Because the compound was used up in the bioassay, the chiral analysis was not conducted. The absolute configuration of **1** remained unknown.

**Fig. 3:**
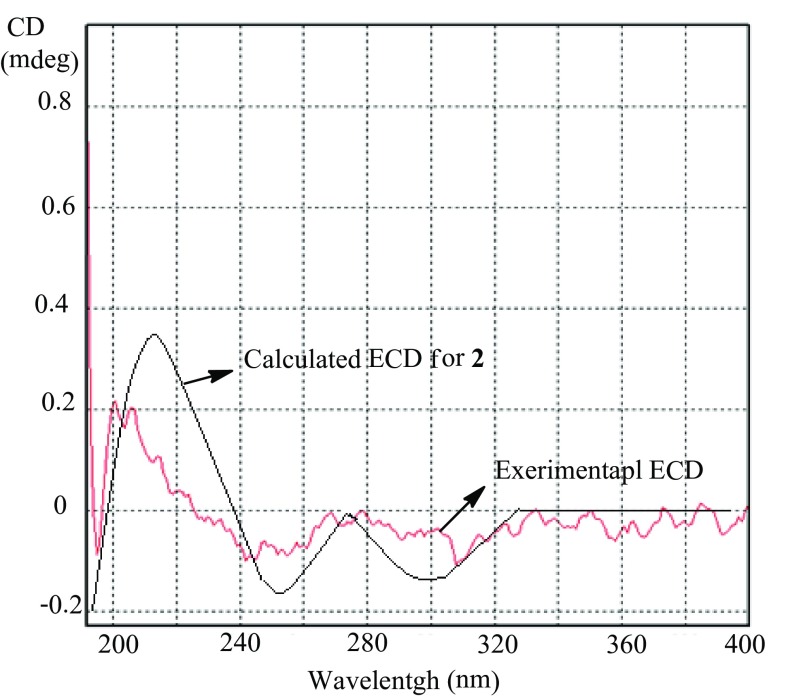
Experimental and calculated ECD spectra for compound **2**

Compound **2** was obtained as a white amorphous powder. Its molecular formula was determined to be C_9_H_12_N_2_O_2_ by ^13^C NMR data and HRESIMS. The ^1^H NMR spectrum exhibited signals for a disubstituted double bond at *δ*_H_ 6.76 (dt, *J* = 9.8, 4.2 Hz) and 5.87 (dt, *J* = 9.8, 1.8 Hz). The ^13^C NMR spectrum indicated the presence of two carbonyl groups, one each at *δ*_C_ 180.7 and 166.6. Comparison of the ^1^H NMR and ^13^C NMR data of **2** with those of 5,6-dihydro-1*H*-pyridin-2-one (**22**) confirmed the presence of a 5,6-dihydro-1*H*-pyridin-2-one moiety [16]. The remaining fragment was deduced to be 3-amino-2-oxopyrrolidine, based on the COSY correlations of H-3′/H_2_-4′ and H_2_-4′/H_2_-5′ and the HMBC correlations from H_2_-5′ to C-2′ and from H_2_-4′ to C-2′ (Fig. 2). These two fragments were found to be connected through a carbon–nitrogen bond based on the HMBC correlations from H-3′ to C-2 and C-6 and from H_2_-6 to C-3′. Thus, the structure of **2** was determined to be 1-(2-oxopyrrolidin-3-yl)-5,6-dihydropyridin-2(1*H*)-one. Several very weak Cotton effects were observed in the ECD spectrum of **2**. At the same time, it was found to have a negative value of optical rotation ($\left[ \alpha \right]_{{\text{D}}}^{{20}}$ − 15.6 (*c* = 0.23, MeOH)). When compound **2** was analyzed by HPLC using a chiral CD-Ph column, only one peak was observed. The chemical calculations of ECD and optical rotation were conducted. Although the Cotton effects were not strong, the calculated ECD spectrum for (*R*)-**2** was mostly consistent with the experimental ECD spectrum for ( − )-**2** (Fig. 3). The calculated value of optical rotation for (*R*)-**2** was negative (− 60). Therefore, the absolute configuration of **2** was suggested to be *R*.

Based on ^13^C NMR data (Table 2) and the HRESIMS ion peak at *m/z* 594.2160 [M + Na]^+^ (calcd for C_26_H_37_NNaO_13_, 594.2163), the molecular formula of retrofractoside A (**3**) was deduced to be C_26_H_37_NO_13_ with nine degrees of unsaturation. The ^1^H and ^13^C NMR data (Table 2) indicated the presence of two *β*-glucopyranosyl groups [*δ*_H_ 4.92 (d, *J* = 7.5 Hz) and 4.91 (d, *J* = 7.6 Hz)], one *E*-double bond [*δ*_H_ 7.48 (d, *J* = 15.5 Hz) and 7.05 (d, *J* = 15.5 Hz)], one trisubstituted phenyl ring [*δ*_H_ 7.58 (s) and 7.26 (2H, overlapped)], and one carbonyl group (*δ*_C_ 167.8). By comparing the NMR data with those of (*E*)-3-phenyl-1-(piperidin-1-yl)prop-2-en-1-one (**17**) and (*E*)-3-(3,4-dihydroxyphenyl)-1-(piperidin-1-yl)prop-2-en-1-one [17, 18], an (*E*)-*N*-caffeoylpiperidine moiety was confirmed, which was supported by the COSY correlations indicating the connection from C-2 to C-6, as well as the HMBC correlations from H_2_-2 to C-6 and C-9′, from H-7′ to C-2′, C-6′, and C-9′, and from H-8′ to C-1′ (Fig. 2). The HMBC correlations (Fig. 2) from H-1′′ to C-3′ and from H-1′′′ to C-4′ suggested that one glucose unit was linked at C-3′, while another unit was linked at C-4′. The configuration of glucose in the plant was determined to be the d-configuration by acidic hydrolysis of piperchabaoside A (**30**) to yield a d-glucopyranose. Therefore, the structure of retrofractoside A (**3**) was elucidated to be (*E*)-*N*-(3,4-di-*O*-*β*-d-glucopyranosyl) caffeoylpiperidine.

**Table 2:** ^1^H (600 MHz) and ^13^C NMR (151 MHz) data of **3** and **4** in methanol-*d*_4_

No.	**3**		**4**	
	*δ*_H_ (*J*, Hz)	*δ*_C_	*δ*_H_ (*J*, Hz)	*δ*_C_
2	3.69 (2H, m)	48.4	3.43 (m) 3.39 (m)	48.8
3	1.64 (2H, m)	28.1	1.29 (2H, m)	27.3
4	1.72 (2H, m)	25.7	1.57 (2H, m)	26.6
5	1.60 (2H, m)	27.1	1.59 (2H, m)	25.5
6	3.65 (2H, m)	44.8	3.67 (m) 3.56 (m)	43.6
1′		132.4		132.7
2′	7.59 (br s)	119.4	7.33 (d, 2.1)	121.0
3′		149.5		149.0
4′		150.7		149.7
5′	7.26 (overlapped)	120.2	7.22 (d, 8.5)	120.3
6′	7.26 (overlapped)	125.7	7.05 (dd, 8.5, 2.1)	125.6
7′	7.48 (d, 15.5)	143.3	6.65 (d, 12.5)	134.1
8′	7.05 (d, 15.5)	118.0	6.03 (d, 12.5)	123.5
9′		167.8		169.7
1′′	4.91 (d, 7.6)	104.2	4.81 (d, 7.6)	104.2
2′′	3.52 (m)	75.3	3.49 (m)	75.2
3′′	3.47 (m)	78.0	3.45 (m)	78.0
4′′	3.37 (dd, 9.7, 8.8)	71.7	3.40 (m)	71.4
5′′	3.41 (m)	78.6	3.37 (m)	78.5
6′′	3.88 (m) 3.69 (m)	62.7	3.90 (dd, 12.0, 2.2) 3.70 (m)	62.6
1′′′	4.92 (d, 7.5)	103.7	4.88 (overlapped)	103.8
2′′′	3.52 (m)	75.1	3.50 (dd, 9.2, 7.4)	75.1
3′′′	3.47 (m)	77.9	3.45 (m)	77.9
4′′′	3.40 (m)	71.4	3.40 (m)	71.4
5′′′	3.41 (m)	78.5	3.37 (m)	78.4
6′′′	3.88 (m) 3.69 (m)	62.6	3.86 (dd, 12.1, 2.0) 3.73 (dd, 12.1, 5.2)	62.5

Retrofractoside B (**4**) was found to have the same molecular formula, C_26_H_37_NO_13_, as that of **3** , based on ^13^C NMR data (Table 2) and HRESIMS. The ^1^H and ^13^C NMR data (Table 2) of **4** also indicated the presence of two *β*-glucopyranosyl groups [*δ*_H_ 4.88 (overlapped, *J*_1′′′,2′′′_ = 7.4 Hz from H-2′′′) and 4.81 (d, *J* = 7.6 Hz)], one trisubstituted phenyl ring [*δ*_H_ 7.33 (d, *J* = 2.1 Hz), 7.22 (d, *J* = 8.5 Hz), and 7.05 (dd, *J* = 8.5, 2.1 Hz)], and one carbonyl group (*δ*_C_ 169.7). However, the double bond in **4** possessed a *Z*-configuration [*δ*_H_ 6.65 (d, *J* = 12.5 Hz) and 6.03 (d, *J* = 12.5 Hz)] rather than an *E*-configuration. Based on the COSY and HMBC correlations (Fig. 2), a (*Z*)-*N*-caffeoylpiperidine moiety in **4** was confirmed. The HMBC correlations (Fig. 2) from H-1′′ to C-3′ and from H-1′′′ to C-4′ suggested the two sugars were located at C-3′ and C-4′, respectively. Thus, the structure of retrofractoside B (**4**) was determined to be (*Z*)-*N*-(3,4-di-*O*-*β*-d-glucopyranosyl) caffeoylpiperidine.

The molecular formula of retrofractoside C (**5**) was determined to be C_26_H_37_NO_12_, based on the ^13^C NMR data (Table 3) and the HRESIMS ion peak at *m/z* 578.2206 [M + Na]^+^ (calcd for C_26_H_37_NNaO_12_, 578.2214). The ^1^H and ^13^C NMR data of **5** (Table 3) indicated the presence of two *β*-glucopyranosyl groups [*δ*_H_ 4.96 (d, *J* = 7.8 Hz) and 4.43 (d, *J* = 7.9 Hz)], a *p*-disubstituted phenyl ring [*δ*_H_ 7.31 (2H, br d, *J* = 8.7 Hz) and 7.07 (2H, br d, *J* = 8.7 Hz)], a *Z*-double bond [*δ*_H_ 6.64 (d, *J* = 12.6 Hz) and 5.97 (d, *J* = 12.6 Hz)], and a carbonyl group [*δ*_C_ 169.8]. By comparing its NMR data with those of **3** and **4**, the amide moiety of **5** was deduced to be (*Z*)-*N*-*p*-coumaroylpiperidine, which was supported by the COSY and HMBC correlations (Fig. 2). The linkage of the sugars and genin was determined to be Glc-(1 → 4)-Glc-O-C-4′ based on the HMBC correlations from H-1′′′ to C-4′′, from H-1′′ to C-4′, from H-4′′ to C-6′′ and C-1′′′ as well as the ROESY correlations of H-1′′/H-5′′ and the COSY correlations of H-5′′/H-6′′ (Fig. 2). Therefore, retrofractoside C (**5**) was determined to be (*Z*)-*N*-*p*-coumaroylpiperidine 4′-*O*-*β*-d-glucopyranosyl-(1 → 4)-*β*-d-glucopyranoside.

**Table 3:** ^1^H (800 MHz) and ^13^C NMR (201 MHz) data of **5** and **6** in methanol-*d*_4_

No.	**5**		**6**	
	*δ*_H_ (*J*, Hz)	*δ*_C_	*δ*_H_ (*J*, Hz)	*δ*_C_
2	3.39 (2H, m)	48.7	3.40 (2H, m)	48.7
3	1.25 (2H, m)	27.1	1.26 (2H, m)	27.2
4	1.57 (2H, m)	25.4	1.57 (2H, m)	25.3
5	1.54 (2H, m)	26.3	1.54 (2H, m)	26.4
6	3.58 (2H, m)	43.3	3.59 (2H, m)	43.3
1′		131.2		131.9
2′	7.31 (br d, 8.7)	130.9	7.05 (d, 1.8)	113.5
3′	7.07 (br d, 8.7)	117.7		150.8
4′		159.2		148.2
5′	7.07 (br d, 8.7)	117.7	7.11 (d, 8.4)	117.6
6′	7.31 (br d, 8.7)	130.9	6.92 (dd, 8.4, 1.8)	122.9
7′	6.64 (d, 12.6)	134.0	6.64 (d, 12.6)	134.1
8′	5.97 (d, 12.6)	122.6	6.00 (d, 12.6)	122.9
9′		169.8		169.8
1′′	4.96 (d, 7.8)	101.7	4.96 (d, 7.7)	102.1
2′′	3.51 (dd, 9.1, 7.8)	74.6	3.56 (m)	74.6
3′′	3.62 (dd, 9.1, 8.9)	76.3	3.62 (dd, 9.1, 8.9)	76.2
4′′	3.66 (m)	80.1	3.68 (dd, 9.7, 8.9)	80.1
5′′	3.59 (m)	76.7	3.55 (m)	76.7
6′′	3.89 (2H, m)	61.6	3.87 (2H, m)	61.6
1′′′	4.43 (d, 7.9 Hz)	104.6	4.44 (d, 7.9)	104.6
2′′′	3.23 (dd, 9.2, 7.9)	74.9	3.23 (dd, 9.1, 7.9)	74.9
3′′′	3.36 (m)	77.9	3.37 (dd, 9.1, 8.9)	77.9
4′′′	3.32 (m)	71.4	3.32 (m)	71.4
5′′′	3.35 (m)	78.2	3.34 (m)	78.2
6′′′	3.88 (dd, 12.0, 2.1) 3.66 (m)	62.5	3.88 (m) 3.66 (m)	62.4
3′-OMe			3.82 (3H, s)	56.7

Retrofractoside D (**6**) was found to have the molecular formula, C_27_H_39_NO_13,_ based on ^13^C NMR data (Table 3) and HRESIMS. The ^1^H and ^13^C NMR data (Table 3) of **6** indicated the presence of two *β*-glucopyranosyl groups [*δ*_H_ 4.96 (d, *J* = 7.7 Hz) and 4.44 (d, *J* = 7.9 Hz)], one 1,2,4-trisubstituted phenyl ring [*δ*_H_ 7.11 (d, *J* = 8.4 Hz), 7.05 (d, *J* = 1.8 Hz), and 6.92 (dd, *J* = 8.4, 1.8 Hz)], one *Z*-double bond [*δ*_H_ 6.64 (d, *J* = 12.6 Hz) and 6.00 (d, *J* = 12.6 Hz)], a carbonyl group [*δ*_C_ 169.8], and a methoxy group [*δ*_H_ 3.82 (3H, s); *δ*_C_ 56.7]. Based on the HMBC correlations from OMe to C-3′ (Fig. 2), the methoxy group was located at C-3′. The amide moiety of **6** was deduced to be (*Z*)-*N*-feruloylpiperidine from the COSY and HMBC correlations (Fig. 2). The linkage of the sugars and genin in **6** was found to be the same mode, Glc-(1 → 4)-Glc-O-C-4′, as that in **5**, based on the HMBC correlations from H-1′′′ to C-4′′, from H-1′′ to C-4′, and from H-4′′ to C-6′′ and C-1′′′ as well as the ROESY correlations of H-1′′/H-5′′ and the COSY correlations H-5′′/H-6′′ (Fig. 2). Accordingly, retrofractoside D (**6**) was determined to be (*Z*)-*N*-feruloylpiperidine 4′-*O*-*β*-d-glucopyranosyl-(1 → 4)-*β*-d-glucopyranoside.

Based on ^13^C NMR (Table 4) and HRESIMS data, the retrofractosides E (**7**) and F (**8**) were determined to have the same molecular formula, C_27_H_40_O_16_. The ^1^H and ^13^C NMR data (Table 4) of **7** and **8** indicated the presence of three *β*-glucopyranosyl groups [*δ*_H_ 4.66 (d, *J* = 7.9 Hz), 4.51 (d, *J* = 7.8 Hz), and 4.39 (d, *J* = 7.9 Hz) in **7**; *δ*_H_ 4.40 (d, *J* = 7.9 Hz), 4.38 (d, *J* = 7.9 Hz), and 4.31 (d, *J* = 7.7 Hz) in **8**] and an (*E*)-cinnamyl alcohol moiety [*δ*_H_ 6.69 (br d, *J* = 16.0 Hz) and 6.37 (dt, *J* = 16.0, 5.9 Hz) in **7**; *δ*_H_ 6.68 (br d, *J* = 16.0 Hz) and 6.36 (dt, *J* = 16.0, 6.0 Hz) in **8**] in these two compounds, which could derive from rozin (**29)** [19] or piperchabaoside A (**30**) [20]. The sugar chain of **7** was elucidated to be Glc-(1 → 4)-Glc-(1 → 2)-Glc based on the HMBC correlations from H-1′′′ to C-4′′, from H-4′′ to C-1′′′, from H-1′′ to C-2′, and from H-2′ to C-1′′ as well as the COSY correlations of H-1′/H-2′ and H-4′′/H-5′′ and the ROESY correlations of H-1′′/H-5′′. According to the HMBC correlations from H-1′′′ to C-6′′, from H_2_-6′′ to C-1′′′, from H-1′′ to C-4′, from H-4′ to C-1′′, and from H-6′a to C-4′ as well as the COSY correlations of H-4′/H-5′ and H-5′/H_2_-6′ and the ROESY correlations of H-1′/H-5′, the sugar chain of **8** was deduced to be Glc-(1 → 6)-Glc-(1 → 4)-Glc. The sugar chains of both compounds were found to be attached to the C-9 position of aglycone, based on the HMBC correlations from H-1′ to C-9 and from H_2_-9 to C-1′. Therefore, the structures of retrofractosides E (**7**) and F (**8**) were determined to be (*E*)-cinnamyl alcohol 9-*O*-*β*-d-glucopyranosyl-(1 → 4)-*β*-d-glucopyranosyl-(1 → 2)-*β*-d-glucopyranoside and (*E*)-cinnamyl alcohol 9-*O*-*β*-d-glucopyranosyl-(1 → 6)-*β*-d-glucopyranosyl-(1 → 4)-*β*-d-glucopyranoside, respectively.

**Table 4:** ^1^H (800 MHz) and ^13^C NMR (201 MHz) data of **7** and **8** in methanol-*d*_4_

No.	**7**		**8**	
	*δ*_H_	*δ*_C_	*δ*_H_	*δ*_C_
1		138.2		138.2
2,6	7.42 (2H, br d, 7.5)	127.6	7.40 (2H, br d, 7.5)	127.5
3,5	7.30 (2H, br t, 7.5)	129.6	7.29 (2H, br t, 7.5)	129.6
4	7.21 (br t, 7.5)	128.7	7.22 (br t, 7.5)	128.7
7	6.69 (br d, 16.0)	133.6	6.68 (br d, 16.0)	133.8
8	6.37 (dt, 16.0, 5.9)	126.7	6.36 (dt, 16.0, 6.0)	126.7
9	4.54 (ddd, 12.8, 5.9, 1.6) 4.35 (ddd, 12.8, 5.9, 1.6)	70.9	4.51 (ddd, 12.5, 6.0, 1.5) 4.32 (ddd, 12.5, 6.0, 1.5)	70.8
1′	4.51 (d, 7.8)	102.1	4.40 (d, 7.9)	103.1
2′	3.47 (dd, 8.9, 7.8)	83.5	3.30 (m)	74.7
3′	3.57 (dd, 9.2, 8.9)	78.1	3.52 (m)	76.7
4′	3.30 (m)	71.4	3.53 (m)	82.3
5′	3.27 (m)	77.8	3.42 (m)	76.2
6′a 6′b	3.86 (m) 3.66 (m)	62.7	3.91 (dd, 12.1, 2.6) 3.83 (dd, 12.1, 4.6)	62.1
1′′	4.66 (d, 7.9)	105.1	4.38 (d, 7.9)	104.9
2′′	3.31 (m)	75.7	3.22 (m)	75.1
3′′	3.53 (dd, 9.1, 9.0)	76.2	3.27 (m)	77.8
4′′	3.59 (dd, 9.4, 9.1)	80.5	3.28 (m)	71.9
5′′	3.41 (m)	76.8	3.56 (m)	76.6
6′′	3.85 (2H, m)	61.8	4.25 (dd, 10.7, 2.2) 3.65 (m)	70.1
1′′′	4.39 (d, 7.9)	104.6	4.31 (d, 7.7)	104.4
2′′′	3.20 (dd, 9.2, 7.9)	74.9	3.22 (m)	74.9
3′′′	3.34 (m)	77.9	3.37 (m)	77.7
4′′′	3.30 (m)	71.4	3.28 (m)	71.5
5′′′	3.34 (m)	78.0	3.35 (m)	77.9
6′′′	3.86 (m) 3.66 (m)	62.4	3.86 (dd, 12.1, 1.8) 3.66 (m)	62.7

(*E*)-*N*-(Tetrahydro-2*H*-pyran-2-yl)cinnamamide (**9**) was first reported as a natural product. It has also been synthesized [21]. The NMR data for compound **9** are presented in this paper. The other known compounds, which are piperine (**10**) [22], methyl piperate (**11**) [23], piperanine (**12**) [24], pipernonaline (**13**) [25], piperchabamide B (**14**) [9], piperolein-B (**15**) [22], 3-phenyl-1-(piperidin-1-yl)propan-1-one (**16**) [17] (*E*)-3-phenyl-1-(piperidin-1-yl)prop-2-en-1-one (**17**) [17], *trans*-fagaramide (**18**) [26], pipercide (**19**) [27], guineensine (**20**) [27], dihydropiperlonguminine (**21**) [25], 5,6-dihydro-1*H*-pyridin-2-one (**22**) [28], 3-chloro-4-hydroxy-2-piperidone (**23**) [29], octahydro-4-hydroxy-3*α*-methyl-7-methylene-*α*-(1-methylethyl)-1*H*-indene-1-methanol (**24**) [30], alismoxide (**25**) [31] (4*S,*4a*S,*6*S,*8a*S*)-octahydro-4-hydroxy-4,8a-dimethyl-6-(1-methylethenyl)naphthalen-1(*2H*)-one (**26**) [16], *ent*-4(15)-eudesmene-1*β*,6*α*-diol (**27**) [32], methylsalicylate-2-*O*-*β*-d-glucopyranoside (**28**) [33], rozin (**29**) [19], piperchabaoside A (**30**) [20] (6*S*,9*R*)-roseoside (**31**) [34], and 2′-*O*-methyluridine (**32**) [35], were identified by comparing their spectroscopic data with those in the literature.

### In Vitro Platelet Aggregation Assay

All the isolates (**1**–**32**) were evaluated for inhibitory activity against mouse platelet aggregation induced by the peptide H–Ala–Tyr–Pro–Gly–Lys–Phe–NH_2_ (AYPGKF-NH_2_). As shown in Table 5, (*E*)-*N*-cinnamoyl-2-methoxypiperidine (**1**), (*E*)-*N*-(tetrahydro-2*H*-pyran-2-yl)cinnamamide (**9**), 3-phenyl-1-(piperidin-1-yl)propan-1-one (**16**), and 2′-*O*-methyluridine (**32**) showed weak inhibitory effects at a concentration of 100 μM, with inhibitory rates of 21.4% (75 μM AYPGKF-NH_2_), 35.5% (75 μM AYPGKF-NH_2_), 26.2% (75 μM AYPGKF-NH_2_), and 36.1% (100 μM AYPGKF-NH_2_). The strongest inhibitory activity was exhibited by 150 μM of compound **9**, with an inhibition of 52.0%. The other tested compounds were found to be inactive.

**Table 5:** Inhibitory effects of compounds from *Piper retrofractum *on mouse platelet aggregation induced by AYPGKF-NH_2_

Compound	Concentration (μM)	Inhibition (%)
(*E*)-*N*-Cinnamoyl-2-methoxypiperidine (**1**)	100	21.4^b^
(*E*)-*N*-(Tetrahydro-2*H*-pyran-2-yl)cinnamamide (**9**)	150	52.0^b^
	130	42.5^b^
	100	35.5^b^
	80	7.6^b^
3-Phenyl-1-(piperidin-1-yl)propan-1-one (**16**)	100	26.2^b^
2′-*O*-Methyluridine (**32**)	100	36.1^c^

^a^Inhibition of compounds **2**–**8**, **10**–**15**, and **17**–**31** was less than 20%

^b^Induced by 75 μM AYP-NH_2_

^c^Induced by 100 μM AYP-NH_2_

AYPGKF-NH_2_ is the gold agonist of protease-activated receptor 4 (PAR4) [36, 37], and PAR4 is believed to be a novel anti-platelet target with low bleeding liability [38, 39]. Therefore, if compound **9** were able to inhibit the aggregation of platelets induced by AYPGKF-NH_2_, then it would be a novel anti-platelet agent.

## Experimental Section

### General Experimental Procedures

Optical rotations were recorded using a JASCO P-1020 Polarimeter (Jasco Corp., Tokyo, Japan). Ultraviolet (UV) spectra were obtained using a Shimadzu UV-2401 PC spectrophotometer (Shimadzu, Kyoto, Japan). ^1^H and ^13^C Nuclear magnetic resonance (NMR) spectra were collected on a Bruker AM-400, DRX-500, Avance III-600, and Ascend™ 800 MHz spectrometers (Bruker Corp., Karlsruhe, Germany) with tetramethylsilane (TMS) as an internal standard. Electrospray ionization mass spectrometry (ESIMS) and high-resolution electrospray ionization mass spectrometry (HRESIMS) analyses were performed on an API QSTAR Pulsar 1 spectrometer (Applied Biosystems/MDS Sciex, Foster City, CA, USA). HREIMS was performed on a Waters AutoSpec Premier p776 spectrometer (Waters, Milford, MA, USA). Silica gel G (80–100 and 300–400 mesh, Qingdao Meigao Chemical Co., Ltd., Qingdao, China), C_18_ silica gel (40–75 μm, Fuji Silysia Chemical Ltd., Aichi, Japan), and Sephadex LH-20 (GE Healthcare Bio-Sciences AB, Uppsala, Sweden) were used for column chromatography, and silica gel GF_254_ (Qingdao Meigao Chemical Co., Ltd.) on precoated plates was used for preparative thin layer chromatography (TLC). TLC spots were visualized under UV light at 254 nm and by dipping into 5% H_2_SO_4_ in alcohol followed by heating. Semipreparative high-performance liquid chromatography (HPLC) was performed on an Agilent 1200 series pump (Agilent Technologies, Santa Clara, USA) equipped with a diode array detector and a Waters XBridge C_18_ column (5.0 μm, *ϕ* 10 × 250 mm), an Agilent Zorbax SB-C_18_ column (5.0 μm, *ϕ* 9.4 × 250 mm), a Welch Ultimate AQ-C_18_ column (5.0 μm, *ϕ* 4.6 × 300 mm), a Welch Ultimate AQ-C_18_ column (5.0 μm, *ϕ* 7.8 × 250 mm), and a chiral CD-Ph column (Shiseido, Japan, 5.0 μm, *ϕ* 4.6 × 250 mm).

### Plant Material

Fruits of *P. retrofractum* were bought from Zay cho Market of Mandalay, Myanmar in December 2015. The plant was identified by the author Jun Yang. A voucher specimen (No. my-80) was deposited at the Key Laboratory of Economic Plants and Biotechnology, Kunming Institute of Botany, Chinese Academy of Sciences.

### Extraction and Isolation

The air-dried fruits of *P. retrofractum* (2.4 kg) were extracted with 90% EtOH (10 L) three times under ambient temperature. The crude extract (260.9 g) was then suspended in H_2_O (1 L) and successively partitioned with petroleum ether (PE, 3 × 1 L), ethyl acetate (EtOAc, 3 × 1 L), and *n*-butanol (*n*-BuOH, 3 × 1 L). After evaporation of the solvent in vacuum, the PE fraction (113.1 g), EtOAc fraction (83.9 g), and *n*-BuOH fraction (43.4 g) were obtained.

The PE fraction (A) was separated by a silica gel column via elution by PE/EtOAc (100:0 → 1:1, v/v) to yield compounds **10** (5.0 g) and **11** (3.5 g), along with nine fractions (Fr. A-1 to Fr. A-9). Fr. A-9 was purified by a C_18_ silica gel column and eluted with MeOH/H_2_O (10% → 100%), to yield seven fractions (Fr. A-9-1 to Fr. A-9-7). Fr. A-9-3 was applied to a silica gel column and eluted with PE/Acetone (20:1 → 1:1) to afford four parts (Fr. A-9-3-1 to Fr. A-9-3-4). Fr. A-9-3-4 was further purified by HPLC (Agilent Zorbax SB-C_18_ column, 5.0 μm, *ϕ* 9.4 × 250 mm, MeOH-H_2_O, 10:90, v/v, 2 mL/min) to give compounds **9** (17.5 mg, *t*_R_ = 13.225 min) and **25** (3.2 mg, *t*_R_ = 6.482 min). Fr. A-9-4 was separated on a silica gel column, with elution by PE/Acetone (15:1), to provide Fr. A-9-4-1 to Fr. A-9-4-3. Fr. A-9-4-1 was further purified by HPLC (Agilent Zorbax SB-C_18_ column, 5.0 μm, *ϕ* 9.4 × 250 mm, MeOH-H_2_O, 60:40, 2 mL/min) to afford compounds **1** (1.5 mg, *t*_R_ = 52.230 min) and **16** (4.0 mg, *t*_R_ = 48.003 min). Fr. A-9-4-2 was separated into two parts by a Sephadex LH-20 column, eluted with MeOH. Fr. A-9-4-2-1 was then purified by HPLC (Welch Ultimate AQ-C_18_ column, 5.0 μm, *ϕ* 7.8 × 250 mm, CH_3_CN-H_2_O, 30:70, 2 mL/min) to afford compounds **24** (1.7 mg, *t*_R_ = 56.221 min), **26** (0.7 mg, *t*_R_ = 63.039 min), and **27** (21.3 mg, *t*_R_ = 36.159 min).

Compound **17** (40.0 mg, *t*_R_ = 30.258 min) was obtained from Fr. A-9-4-2-2 by HPLC (Agilent Zorbax SB-C_18_ column, 5.0 μm, *ϕ* 9.4 × 250 mm, CH_3_CN-H_2_O, 40:60, 2 mL/min). Fr. A-9-4-3 was applied to a Sephadex LH-20 column and eluted with MeOH to afford compound **18** (17.5 mg). The remaining part was then further purified by HPLC (Agilent Zorbax SB-C_18_ column, 5.0 μm, *ϕ* 9.4 × 250 mm, MeOH-H_2_O, 60:40, 2 mL/min) to provide compound **21** (9.4 mg, *t*_R_ = 36.088 min). Fr. A-9-5 was applied to a silica gel column, with elution by PE/EtOAc (20:1), and a Sephadex LH-20 column, with elution by MeOH, yielding compound **12** (7.4 mg). Fr. A-9-7 was separated by a silica gel column via elution by PE/Acetone (10:1) to afford compounds **13** (5.7 g), **19** (7.2 g), and **20** (1.2 g). Finally, the remaining part of Fr. A-9-7 was applied to a Sephadex LH-20 column, eluted with MeOH and then further purified by HPLC (Agilent Zorbax SB-C_18_ column, 5.0 μm, *ϕ* 9.4 × 250 mm, MeOH-H_2_O, 85:15, 2 mL/min), yielding compounds **14** (4.5 mg, *t*_R_ = 25.337 min) and **15** (4.5 mg, *t*_R_ = 17.900 min).

The *n*-BuOH fraction (C) was separated by a silica gel column via elution by CH_2_Cl_2_/MeOH (20:1 → 1:1, v/v), to yield five fractions (Fr. C-1 to Fr. C-5).

Fr. C-1 was applied to a C_18_ silica gel column and eluted with MeOH/H_2_O (5% → 100%) to yield fractions Fr. C-1--1 and Fr. C-1-2. Fr.C-1-1 was further purified using HPLC (Welch Ultimate AQ-C_18_ column, 5.0 μm, *ϕ* 7.8 × 250 mm, MeOH-H_2_O, 7:93, 2 mL/min) to afford compound **23** (2.3 mg, *t*_R_ = 10.681 min). Fr. C-1-2 was applied to a Sephadex LH-20 column, eluted with MeOH, and then purified by HPLC (Welch Ultimate AQ-C_18_ column, 5.0 μm, *ϕ* 7.8 × 250 mm, MeOH-H_2_O, 7:93, 2 mL/min), and compounds **2** (4.0 mg, *t*_R_ = 36.216 min) and **22** (7.4 mg, *t*_R_ = 15.353 min) were obtained.

Fr. C-2 was separated by a C_18_ silica gel column via elution by MeOH/H_2_O (5% → 100%), to yield four fractions (Fr. C-2-1 to Fr. C-2-4). Fr. C-2-2 was applied to a Sephadex LH-20 column, eluted with MeOH, and then purified by HPLC (Welch Ultimate AQ-C_18_ column, 5.0 μm, *ϕ* 7.8 × 250 mm, MeOH-H_2_O, 7:93, 2 mL/min) to provide compound **32** (1.8 mg, *t*_R_ = 24.453 min). Fr. C-2-3 was applied to a Sephadex LH-20 column and eluted with MeOH to afford two fractions. Fr. C-2-3-1 was purified by HPLC (Welch Ultimate AQ-C_18_ column, 5.0 μm, *ϕ* 7.8 × 250 mm, MeOH-H_2_O, 40:60, 2 mL/min) to give compound **31** (10.7 mg, *t*_R_ = 8.977 min), and compound **28** (1.2 mg, *t*_R_ = 9.417 min) was obtained from Fr. C-2-3-2, purified by HPLC (Welch Ultimate AQ-C_18_ column, 5.0 μm, *ϕ* 7.8 × 250 mm, MeOH-H_2_O, 38:52, 2 mL/min). Fr. C-2-4 was separated by a silica gel column, with elution by CH_2_Cl_2_/MeOH (50:1 → 1:1, v/v), and then purified by HPLC (Welch Ultimate AQ-C_18_ column, 5.0 μm, *ϕ* 7.8 × 250 mm, MeOH-H_2_O, 65:35, 2 mL/min), and compound **29** (3.6 mg, *t*_R_ = 6.597 min) was obtained.

Fr. C-4 was applied to a C_18_ silica gel column and eluted with MeOH/H_2_O (5% → 100%) to yield fractions Fr. C-4-1 and Fr. C-4-2. Fr. C-4-1 was further purified by a silica gel column, with elution by EtOAc/MeOH (15:1), to provide compound **30** (8.6 mg). Fr. C-4-2 was applied to a Sephadex LH-20 column, eluted with MeOH, and then purified by HPLC (Welch Ultimate AQ-C_18_ column, 5.0 μm, *ϕ* 7.8 × 250 mm, MeOH-H_2_O, 27:73, 2 mL/min) to afford compounds **5** (0.7 mg, *t*_R_ = 7.802 min) and **6** (0.8 mg, *t*_R_ = 14.055 min).

Fr. C-5 was separated on a C_18_ silica gel column via elution by MeOH/H_2_O (5% → 100%), to yield fraction Fr. C-5-1. Then, Fr. C-5-1 was applied to a Sephadex LH-20 column and eluted with MeOH to yield two fractions. Fr. C-5-1-1 was further applied to a silica gel column, eluted with EtOAc/MeOH (10:1), and then purified by HPLC (Welch Ultimate AQ-C_18_ column, 5.0 μm, *ϕ* 7.8 × 250 mm, CH_3_CN-H_2_O, 15:85, 2 mL/min) to afford compounds **3** (2.1 mg, *t*_R_ = 14.066 min), **4** (2.1 mg, *t*_R_ = 11.151 min), **7** (0.5 mg, *t*_R_ = 15.715 min), and **8** (0.7 mg, *t*_R_ = 26.034 min).

### Spectroscopic Data of Compounds

#### (*E*)-*N*-Cinnamoyl-2-methoxypiperidine (**1**)

White amorphous powder; $\left[ \alpha \right]_{{\text{D}}}^{{23}}$ − 15.3 (*c* = 0.15, MeOH); UV (MeOH) *λ*_max_ (log*ε* ) 399 (1.29), 279 (3.66), 217 (3.52), 205 (3.53) nm; ^1^H NMR and ^13^C NMR data see Table 1; ESIMS (positive) *m/z* 268 [M + Na]^+^, 513 [2M + Na]^+^; HREIMS *m/z* 245.1423 [M]^+^ (calcd for C_15_H_19_NO_2_, 245.1416).

#### (*R*)-1-(2-Oxopyrrolidin-3-yl)-5,6-dihydropyridin-2(1*H*)-one (**2**)

White amorphous powder; $\left[ \alpha \right]_{{\text{D}}}^{{20}}$ − 15.6 (*c* = 0.23, MeOH); UV (MeOH) *λ*_max_ (log*ε* ) 246 (2.32), 232 (2.27), 196 (3.05) nm; ^1^H NMR (CD_3_OD, 500 MHz) *δ*_H_ 6.76 (dt, *J* = 9.8, 4.2 Hz, H-4), 6.23 (m, H-3′), 5.87 (dt, *J* = 9.8, 1.8 Hz, H-3), 3.38 (2H, m, H_2_-6), 2.51 (m, H-5′a), 2.51 (m, H-4′a), 2.44 (2H, m, H_2_-5), 2.36 (m, H-5′b), 2.01 (m, H-4′b); ^13^C NMR (CD_3_OD, 126 MHz) *δ*_C_ 180.7 (C-2′), 166.6 (C-2), 143.6 (C-4), 125.2 (C-3), 65.0 (C-3′), 39.7 (C-6), 30.5 (C-5′), 25.7 (C-4′), 25.3 (C-5); ESIMS (positive) *m/z* 203 [M + Na]^+^, 383 [2 M + Na]^+^; HRESIMS (positive) *m/z* 203.0793 [M + Na]^+^ (calcd for C_9_H_12_N_2_NaO_2_, 203.0797).

#### Retrofractoside A (**3**)

White amorphous powder; $\left[ \alpha \right]_{{\text{D}}}^{{19}}$ − 120.2 (*c* = 0.10, MeOH); UV (MeOH) *λ*_max_ (log*ε* ) 290 (4.33), 248 (3.93), 213 (4.28), 207 (4.28), 196 (4.33) nm; ^1^H NMR and ^13^C NMR data see Table 2; ESIMS (positive) *m/z* 594 [M + Na]^+^; HRESIMS (positive) *m/z* 594.2160 [M + Na]^+^ (calcd for C_26_H_37_NNaO_13_, 594.2163).

#### Retrofractoside B (**4**)

White amorphous powder; $\left[ \alpha \right]_{{\text{D}}}^{{21}}$ − 73.5 (*c* = 0.12, MeOH); UV (MeOH) *λ*_max_ (log*ε*) 264 (3.95), 242 (3.82), 197 (4.30) nm; ^1^H NMR and ^13^C NMR data see Table 2; ESIMS (positive) *m/z* 594 [M + Na]^+^; HRESIMS (positive) *m/z* 594.2161 [M + Na]^+^ (calcd for C_26_H_37_NNaO_13_, 594.2163).

#### Retrofractoside C (**5**)

White amorphous powder; $\left[ \alpha \right]_{{\text{D}}}^{{27}}$ − 67.5 (*c* = 0.04, MeOH); UV (MeOH) *λ*_max_ (log*ε* ) 265 (3.83), 203 (4.06) nm; ^1^H NMR and ^13^C NMR data see Table 3; ESIMS (positive) *m/z* 578 [M + Na]^+^; HRESIMS (positive) *m/z* 578.2206 [M + Na]^+^ (calcd for C_26_H_37_NNaO_12_, 578.2214).

#### Retrofractoside D (**6**)

White amorphous powder; $\left[ \alpha \right]_{{\text{D}}}^{{22}}$ − 52.6 (*c* = 0.10, MeOH); UV (MeOH) *λ*_max_ (log*ε* ) 293 (3.96), 266 (4.16), 205 (4.46) nm; ^1^H NMR and ^13^C NMR data see Table 3; ESIMS (positive) *m/z* 608 [M + Na]^+^; HRESIMS (positive) *m/z* 608.2317 [M + Na]^+^ (calcd for C_27_H_39_NNaO_13_, 608.2319).

#### Retrofractoside E (**7**)

Brown syrup; $\left[ \alpha \right]_{{\text{D}}}^{{19}}$ − 51.1 (*c* = 0.10, MeOH); UV (MeOH) *λ*_max_ (log*ε* ) 250 (3.51), 231 (3.42), 197 (4.03) nm; ^1^H NMR and ^13^C NMR data see Table 4; ESIMS (positive) *m/z* 643 [M + Na]^+^; HRESIMS (positive) *m/z* 643.2218 [M + Na]^+^ (calcd for C_27_H_40_NaO_16_, 643.2214).

#### Retrofractoside F (**8**)

Brown syrup; $\left[ \alpha \right]_{{\text{D}}}^{{19}}$ − 67.5 (*c* = 0.08, MeOH); UV (MeOH) *λ*_max_ (log*ε* ) 249 (4.29), 224 (3.90), 200 (4.51) nm; ^1^H NMR and ^13^C NMR data see Table 4; ESIMS (positive) *m/z* 643 [M + Na]^+^; HRESIMS (positive) *m/z* 643.2217 [M + Na]^+^ (calcd for C_27_H_40_NaO_16_, 643.2214).

#### (*E*)-*N*-(Tetrahydro-2*H*-pyran-2-yl)cinnamamide (**9**)

White amorphous powder; $\left[ \alpha \right]_{{\text{D}}}^{{22}}$ − 7.8 (*c* = 0.07, MeOH); UV (MeOH) *λ*_max_ (log*ε* ) 322 (1.71), 276 (2.36), 207 (2.76) nm; ^1^H NMR (CDCl_3_, 600 MHz) *δ*_H_ 7.66 (d, *J* = 15.6 Hz, H-7), 7.50 (2H, m, H-2,6), 7.37 (2H, m, H-3,5), 7.36 (m, H-4), 6.36 (d, *J* = 15.6 Hz, H-8), 5.98 (br d, *J* = 8.3 Hz, NH), 5.26 (m, H-2′), 4.01 (m, H-6′a), 3.66 (td, *J* = 11.2, 3.5 Hz, H-6′b), 1.91 (m, H-4′a), 1.88 (m, H-3′a), 1.65 (m, H-4′b), 1.55 (2H, m, H-5′), 1.45 (m, H-3′b); ^13^C NMR (CDCl_3_, 151 MHz) *δ*_C_ 165.2 (C-9), 142.4 (C-7), 134.8 (C-1), 130.0 (C-4), 129.0 (C-3,5), 128.0 (C-2,6), 120.4 (C-8), 78.1 (C-2′), 67.6 (C-6′), 31.9 (C-3′), 25.2 (C-5′), 23.0 (C-4′); ESIMS (positive) *m/z* 254 [M + Na]^+^, 485 [2M + Na]^+^; HRESIMS (positive) *m/z* 254.1152 [M + Na]^+^ (calcd for C_14_H_17_NNaO_2_, 254.1157).

### Acidic Hydrolysis of Piperchabaoside A (**30**)

Piperchabaoside A (4.6 mg) was dissolved in 2 M HCl (1 mL) and stirred at 90 °C for 3 h. After cooling, the solution was evaporated until dry under reduced pressure. The reaction mixture was purified by silica gel column chromatography (CH_2_Cl_2_-MeOH-H_2_O, 500:10:0, 300:10:0, 200:10:1, 100:10:1) to afford d-glucopyranose (1.2 mg), which was identified based on its ^1^H NMR spectrum and optical rotation value: $\left[ \alpha \right]_{{\text{D}}}^{{21}}$ + 23.9 (*c* 0.12, H_2_O) [40].

### In Vitro Platelet Aggregation Assay

The inhibitory effects of compounds against ICR mice platelet aggregation induced by AYPGKF-NH_2_ were evaluated according to previously published methods [41–44].

#### Animals

Adult Institute of Cancer Research (ICR) mice (30–40 g) were obtained from Nanjing Qinglongshan Animal Centre (Nanjing, Jiangsu province, China). All animals were housed under controlled temperature (21–25 °C) and light (12 h light, 12 h dark) with ad libitum access to food and water for one week before the experiments. All the experiments were performed according to the guidelines and the regulations of the Ethical Committee of China Pharmaceutical University (CPU2016-S07, 5 March 2016).

#### Materials and Reagents

AYPGKF-NH_2_ was purchased from Ningbo Kangbei biochemical Co. Ltd. (Ningbo, Zhejiang province, China). All other chemicals used in this study were of analytical grade.

#### Platelet Preparation

Blood was withdrawn from ICR mice through the abdominal aorta and then anticoagulated with 3.2% sodium citrate (1:9 citrate/blood, v/v). Platelet-rich plasma (PRP) was obtained by centrifugation at 1080 rpm for 10 min. To prepare the gel-filtered platelets, PRP was applied to a column packed with Sepharose 2B beads and eluted with Tyrode’s buffer into a series of 15 mL tubes [41, 43]. The collected platelets in each tube were counted, combined, and adjusted to 2.5 × 10^8^ /mL using Tyrode’s buffer.

#### Platelet Aggregation Assay

In vitro platelet aggregation was measured according to the turbidimetric method, using a four-channel aggregometer (LBY-NJ4, Pulisheng Instrument Co. Ltd., Beijing, China) [42, 44]. Gel-filtered ICR mice platelets were preincubated with samples or vehicles for 5 min at 37 °C. Then, platelet aggregation was induced by AYPGKF-NH_2_. The maximum aggregation rate was measured within 5 min with continuous stirring. The light transmittance was calibrated with Tyrode’s buffer. The percentage (%) of inhibition of platelet aggregation was calculated by the following formula: [(X − Y)/X] × 100%, where X is the maximum aggregation rate of vehicle-treated gel-filtered platelets and Y is the maximum aggregation rate of sample-treated gel-filtered platelets.

## Conclusion

The phytochemical investigation of the fruits of *Piper retrofractum* in this study led to the identification of 32 compounds, including two new amides, four new amide glucosides, and two new phenylpropanoid glucosides. In vitro platelet aggregation assays of all the isolates were conducted, and the results showed that (*E*)-*N*-(tetrahydro-2*H*-pyran-2-yl)cinnamamide (**9**) possessed weak inhibitory activity against mouse platelet aggregation induced by AYPGKF-NH_2_, which is a gold agonist of protease-activated receptor 4.

## Conflict of interest

Authors declare that there are no conflicts of interest associated with this work.

## Electronic supplementary material

Supplementary material associated with this article (1D and 2D NMR and HRMS spectra of new compounds, chemical structures of known compounds, and computational methods). Below is the link to the electronic supplementary material.

Supplementary file1 (PDF 6444 kb)
