# Supplementary material for: New Amides and Phenylpropanoid Glucosides from the Fruits of Piper retrofractum
Source: Nat Prod Bioprospect. 2019 May 9;9(3):231–41. doi: 10.1007/s13659-019-0208-z (PMC6538700; doi:10.1007/s13659-019-0208-z)
Supplement: Supplementary file 1 — Supplementary file1 (PDF 6444 kb) [file 13659_2019_208_MOESM1_ESM.pdf]

# Supplementary Material for

## New Amides and Phenylpropanoid Glucosides from the Fruits of *Piper retrofractum*

Rong Tang[1,3] · Ya-Qiong Zhang[2] · Dong-Bao Hu[4] · Xue-Fei Yang[1,5] · Jun Yang[1,5] · Myint Myint San[6] · Thaung Naing Oo[6] · Yi Kong[2] · Yue-Hu Wang[1,5]

[1] Key Laboratory of Economic Plants and Biotechnology and the Yunnan Key Laboratory for Wild Plant Resources, Kunming Institute of Botany, Chinese Academy of Sciences, Kunming 650201, People's Republic of China

[2] School of Life Science & Technology, China Pharmaceutical University, Nanjing 210009, China

[3] University of Chinese Academy of Sciences, Beijing 100049, People's Republic of China

[4] School of Chemical Biology and Environment, Yuxi Normal University, Yuxi 653100, People's Republic of China

[5] Southeast Asia Biodiversity Research Institute, Chinese Academy of Sciences, Yezin, Nay Pyi Taw 05282, Myanmar

[6] Forest Research Institute, Yezin, Nay Pyi Taw 05282, Myanmar

∗ Corresponding authors:

E-mail address: wangyuehu@mail.kib.ac.cn; yikong668@163.com.

## Table of Contents

**Computational methods**
**Fig. S1** Chemical structures of known compounds (**9**–**32**)
**Fig. S2** $^{1}$H NMR spectrum of **1** in $CDCl_3$
**Fig. S3** $^{13}$C NMR spectrum of **1** in $CDCl_3$
**Fig. S4** HSQC spectrum of **1**
**Fig. S5** $^{1}$H-$^{1}$H COSY spectrum of **1**
**Fig. S6** HMBC spectrum of **1**
**Fig. S7** ROESY spectrum of **1**
**Fig. S8** HREIMS spectrum of **1**
**Fig. S9** UV spectrum of **1**
**Fig. S10** OR spectrum of **1**
**Fig. S11** $^{1}$H NMR spectrum of **2** in methanol-$d_4$
**Fig. S12** $^{13}$C NMR spectrum of **2** in methanol-$d_4$
**Fig. S13** HSQC spectrum of **2**
**Fig. S14** $^{1}$H-$^{1}$H COSY spectrum of **2**
**Fig. S15** HMBC spectrum of **2**
**Fig. S16** ROESY spectrum of **2**
**Fig. S17** HRESIMS spectrum of **2**
**Fig. S18** UV spectrum of **2**
**Fig. S19** OR spectrum of **2**
**Fig. S20** ECD spectrum of **2**
**Fig. S21** $^{1}$H NMR spectrum of **3** in methanol-$d_4$
**Fig. S22** $^{13}$C NMR spectrum of **3** in methanol-$d_4$
**Fig. S23** HSQC spectrum of **3**
**Fig. S24** $^{1}$H-$^{1}$H COSY spectrum of **3**
**Fig. S25** HMBC spectrum of **3**
**Fig. S26** ROESY spectrum of **3**
**Fig. S27** HRESIMS spectrum of **3**
**Fig. S28** UV spectrum of **3**
**Fig. S29** OR spectrum of **3**
**Fig. S30** $^{1}$H NMR spectrum of **4** in methanol-$d_4$
**Fig. S31** $^{13}$C NMR spectrum of **4** in methanol-$d_4$
**Fig. S32** HSQC spectrum of **4**
**Fig. S33** $^{1}$H-$^{1}$H COSY spectrum of **4**
**Fig. S34** HMBC spectrum of **4**
**Fig. S35** ROESY spectrum of **4**
**Fig. S36** HRESIMS spectrum of **4**
**Fig. S37** UV spectrum of **4**
**Fig. S38** OR spectrum of **4**
**Fig. S39** $^{1}$H NMR spectrum of **5** in methanol-$d_4$
**Fig. S40** $^{13}$C NMR spectrum of **5** in methanol-$d_4$
**Fig. S41** HSQC spectrum of **5**

**Fig. S42** $^{1}H$-$^{1}H$ COSY spectrum of **5**
**Fig. S43** HMBC spectrum of **5**
**Fig. S44** ROESY spectrum of **5**
**Fig. S45** HRESIMS spectrum of **5**
**Fig. S46** UV spectrum of **5**
**Fig. S47** OR spectrum of **5**
**Fig. S48** $^{1}H$ NMR spectrum of **6** in methanol-$d_4$
**Fig. S49** $^{13}C$ NMR spectrum of **6** in methanol-$d_4$
**Fig. S50** HSQC spectrum of **6**
**Fig. S51** $^{1}H$-$^{1}H$ COSY spectrum of **6**
**Fig. S52** HMBC spectrum of **6**
**Fig. S53** ROESY spectrum of **6**
**Fig. S54** HRESIMS spectrum of **6**
**Fig. S55** UV spectrum of **6**
**Fig. S56** OR spectrum of **6**
**Fig. S57** $^{1}H$ NMR spectrum of **7** in methanol-$d_4$
**Fig. S58** $^{13}C$ NMR spectrum of **7** in methanol-$d_4$
**Fig. S59** HSQC spectrum of **7**
**Fig. S60** $^{1}H$-$^{1}H$ COSY spectrum of **7**
**Fig. S61** HMBC spectrum of **7**
**Fig. S62** ROESY spectrum of **7**
**Fig. S63** HRESIMS spectrum of **7**
**Fig. S64** UV spectrum of **7**
**Fig. S65** OR spectrum of **7**
**Fig. S66** $^{1}H$ NMR spectrum of **8** in methanol-$d_4$
**Fig. S67** $^{13}C$ NMR spectrum of **8** in methanol-$d_4$
**Fig. S68** HSQC spectrum of **8**
**Fig. S69** $^{1}H$-$^{1}H$ COSY spectrum of **8**
**Fig. S70** HMBC spectrum of **8**
**Fig. S71** ROESY spectrum of **8**
**Fig. S72** HRESIMS spectrum of **8**
**Fig. S73** UV spectrum of **8**
**Fig. S74** OR spectrum of **8**

## Computational methods

All DFT and TD-DFT calculations were carried out at 298 K in the gas phase with Gaussian 09.[1] Conformational searches were carried out at the molecular mechanics level of theory employing MMFF force fields.[2-7] The conformers with relative energy within 10 kcal/mol of the lowest-energy conformer were selected and further geometry optimized at the B3LYP/6-311++G(2d,p) level. All the lowest-energy conformers, which correspond to 99% of the total Boltzmann distribution, were selected for ECD spectra calculation. The Boltzmann factor for each conformer was calculated based on Gibbs free energy. Vibrational analysis at the B3LYP/6-311++G(2d,p) level of theory resulted in no imaginary frequencies, confirming the considered conformers as real minima. TDDFT was employed to calculate excitation energy (in nm) and rotatory strength R in dipole velocity form, at the B3LYP/6-311++G(2d,p) level.

References


[1] Gaussian 09.Rev.Cl; Frisch, M. J., et al. Gaussian, Inc., Pittsburgh PA, 2009.

[2] Mayer Z.A.; Kallay M.; Kubinyi M. ; Keglevich G.*J. M. Strut.* 2009,906, 94-99.

[3] Silva G.V.J.;NetoA.C.*Tetrahedron*.2005,61,7763-7767.

[4] Borkowski E.J.;Suvire F.D.;Enriz R.D. *J. Mol. Struct*. 2010, 953,83-90.

[5] Zhao, S. D.; Shen, L.; Luo, D. Q.; Zhu, H. J. *Curr. Org. Chem.*2011,*15*, 1843-1862.

[6] Li, Q. M.; Ren, J.; Zhou, B. D.; Bai, B.; Liu, X. C.; Wen, M. L.; Zhu, H. J. *Tetrahedron.* 2013, *69*, 3067-3074.

[7] Hu D.B., Li W.X., Zhao Z.Z., Feng T., Yin R.H., Li Z.H., Liu J.K., Zhu H.J. *Tetrahedron Lett.* 2014, 55, 6530–6533.


**Caculated ECD data**

| | |
|---|---|
| 400 | 0 |
| 399.2016 | 0 |
| 398.4064 | 0 |
| 397.6143 | 0 |
| 396.8254 | 0 |
| 396.0396 | 0 |
| 395.2569 | 0 |
| 394.4773 | 0 |
| 393.7008 | 0 |
| 392.9273 | 0 |
| 392.1569 | 0 |
| 391.3894 | 0 |
| 390.625 | 0 |
| 389.8635 | 0 |
| 389.1051 | 0 |
| 388.3495 | 0 |
| 387.5969 | 0 |
| 386.8472 | 0 |
| 386.1004 | 0 |

| | |
|---|---|
| 385.3565 | 0 |
| 384.6154 | 0 |
| 383.8772 | 0 |
| 383.1418 | 0 |
| 382.4092 | 0 |
| 381.6794 | 0 |
| 380.9524 | 0 |
| 380.2281 | 0 |
| 379.5066 | 0 |
| 378.7879 | 0 |
| 378.0718 | 0 |
| 377.3585 | 0 |
| 376.6478 | 0 |
| 375.9399 | 0 |
| 375.2345 | 0 |
| 374.5318 | 0 |
| 373.8318 | 0 |
| 373.1343 | 0 |
| 372.4395 | 0 |
| 371.7472 | 0 |
| 371.0575 | 0 |
| 370.3704 | 0 |
| 369.6858 | 0 |
| 369.0037 | 0 |
| 368.3241 | 0 |
| 367.6471 | 0 |
| 366.9725 | 0 |
| 366.3004 | 0 |
| 365.6307 | 0 |
| 364.9635 | 0 |
| 364.2987 | 0 |
| 363.6364 | 0 |
| 362.9764 | 0 |
| 362.3188 | 0 |
| 361.6637 | 0 |
| 361.0108 | 0 |
| 360.3604 | 0 |
| 359.7122 | 0 |
| 359.0664 | 0 |
| 358.4229 | 0 |
| 357.7818 | 0 |
| 357.1429 | 0 |
| 356.5062 | 0 |
| 355.8719 | 0 |

| | |
|---|---|
| 355.2398 | 0 |
| 354.6099 | 0 |
| 353.9823 | 0 |
| 353.3569 | 0 |
| 352.7337 | 0 |
| 352.1127 | 0 |
| 351.4938 | 0 |
| 350.8772 | 0 |
| 350.2627 | 0 |
| 349.6504 | 0 |
| 349.0401 | 0 |
| 348.4321 | 0 |
| 347.8261 | 0 |
| 347.2222 | 0 |
| 346.6205 | 0 |
| 346.0208 | 0 |
| 345.4231 | 0 |
| 344.8276 | 0 |
| 344.2341 | 0 |
| 343.6426 | 0 |
| 343.0532 | 0 |
| 342.4658 | 0 |
| 341.8803 | 0 |
| 341.2969 | 0 |
| 340.7155 | 0 |
| 340.1361 | 0 |
| 339.5586 | 0 |
| 338.9831 | 0 |
| 338.4095 | 0 |
| 337.8378 | 0 |
| 337.2681 | 0 |
| 336.7003 | 0 |
| 336.1345 | 0 |
| 335.5705 | 0 |
| 335.0084 | 0 |
| 334.4482 | 0 |
| 333.8898 | 0 |
| 333.3333 | 0 |
| 332.7787 | 0 |
| 332.2259 | 0 |
| 331.675 | 0 |
| 331.1258 | 0 |
| 330.5785 | 0 |
| 330.033 | 0 |

| | |
|---|---|
| 329.4893 | 0 |
| 328.9474 | 0 |
| 328.4072 | 0 |
| 327.8689 | 0 |
| 327.3322 | 0 |
| 326.7974 | 0 |
| 326.2643 | 0 |
| 325.7329 | 0 |
| 325.2033 | 0 |
| 324.6753 | 0 |
| 324.1491 | 0 |
| 323.6246 | 0 |
| 323.1018 | 0 |
| 322.5806 | 0 |
| 322.0612 | 0 |
| 321.5434 | 0 |
| 321.0273 | 0 |
| 320.5128 | 0 |
| 320 | 0 |
| 319.4888 | 0 |
| 318.9793 | 0 |
| 318.4713 | 0 |
| 317.965 | 0 |
| 317.4603 | 0 |
| 316.9572 | 0 |
| 316.4557 | 0 |
| 315.9558 | 0 |
| 315.4574 | 0 |
| 314.9606 | 0 |
| 314.4654 | 0 |
| 313.9717 | 0 |
| 313.4796 | 0 |
| 312.989 | 0 |
| 312.5 | 0 |
| 312.0125 | 0 |
| 311.5265 | 0 |
| 311.042 | 0 |
| 310.559 | 0 |
| 310.0775 | 0 |
| 309.5975 | 0 |
| 309.119 | 0 |
| 308.642 | -1E-06 |
| 308.1664 | -1E-06 |
| 307.6923 | -2E-06 |

| | |
|---|---|
| 307.2197 | -3E-06 |
| 306.7485 | -4E-06 |
| 306.2787 | -7E-06 |
| 305.8104 | -1.1E-05 |
| 305.3435 | -1.7E-05 |
| 304.878 | -2.6E-05 |
| 304.414 | -4.1E-05 |
| 303.9514 | -6.3E-05 |
| 303.4901 | -9.6E-05 |
| 303.0303 | -0.00015 |
| 302.5719 | -0.00022 |
| 302.1148 | -0.00033 |
| 301.6591 | -0.00049 |
| 301.2048 | -0.00072 |
| 300.7519 | -0.00105 |
| 300.3003 | -0.00153 |
| 299.8501 | -0.00219 |
| 299.4012 | -0.00313 |
| 298.9537 | -0.00444 |
| 298.5075 | -0.00624 |
| 298.0626 | -0.0087 |
| 297.619 | -0.01204 |
| 297.1768 | -0.01653 |
| 296.7359 | -0.02253 |
| 296.2963 | -0.03047 |
| 295.858 | -0.04089 |
| 295.421 | -0.05446 |
| 294.9853 | -0.07196 |
| 294.5508 | -0.09436 |
| 294.1176 | -0.12278 |
| 293.6858 | -0.15854 |
| 293.2551 | -0.20313 |
| 292.8258 | -0.25827 |
| 292.3977 | -0.32585 |
| 291.9708 | -0.40794 |
| 291.5452 | -0.5068 |
| 291.1208 | -0.62478 |
| 290.6977 | -0.7643 |
| 290.2758 | -0.92779 |
| 289.8551 | -1.11759 |
| 289.4356 | -1.33587 |
| 289.0173 | -1.58451 |
| 288.6003 | -1.86498 |
| 288.1844 | -2.17822 |

| | |
|---|---|
| 287.7698 | -2.52453 |
| 287.3563 | -2.9034 |
| 286.944 | -3.31347 |
| 286.533 | -3.75239 |
| 286.123 | -4.2168 |
| 285.7143 | -4.70228 |
| 285.3067 | -5.20336 |
| 284.9003 | -5.7136 |
| 284.495 | -6.2257 |
| 284.0909 | -6.73162 |
| 283.6879 | -7.22279 |
| 283.2861 | -7.69037 |
| 282.8854 | -8.12546 |
| 282.4859 | -8.51943 |
| 282.0874 | -8.86421 |
| 281.6901 | -9.15255 |
| 281.294 | -9.3783 |
| 280.8989 | -9.53669 |
| 280.5049 | -9.62446 |
| 280.112 | -9.64003 |
| 279.7203 | -9.58361 |
| 279.3296 | -9.45716 |
| 278.94 | -9.2644 |
| 278.5515 | -9.01066 |
| 278.1641 | -8.70271 |
| 277.7778 | -8.34855 |
| 277.3925 | -7.95718 |
| 277.0083 | -7.53825 |
| 276.6252 | -7.10181 |
| 276.2431 | -6.65802 |
| 275.8621 | -6.21679 |
| 275.4821 | -5.78758 |
| 275.1032 | -5.37912 |
| 274.7253 | -4.9992 |
| 274.3484 | -4.65452 |
| 273.9726 | -4.35054 |
| 273.5978 | -4.0914 |
| 273.224 | -3.87989 |
| 272.8513 | -3.7174 |
| 272.4796 | -3.60398 |
| 272.1088 | -3.53838 |
| 271.7391 | -3.51816 |
| 271.3704 | -3.53976 |
| 271.0027 | -3.59865 |

| | |
|---|---|
| 270.636 | -3.68949 |
| 270.2703 | -3.80626 |
| 269.9055 | -3.94249 |
| 269.5418 | -4.09135 |
| 269.179 | -4.24594 |
| 268.8172 | -4.39941 |
| 268.4564 | -4.54515 |
| 268.0965 | -4.677 |
| 267.7376 | -4.78936 |
| 267.3797 | -4.8774 |
| 267.0227 | -4.93709 |
| 266.6667 | -4.96538 |
| 266.3116 | -4.96019 |
| 265.9574 | -4.92047 |
| 265.6043 | -4.84619 |
| 265.252 | -4.73829 |
| 264.9007 | -4.5986 |
| 264.5503 | -4.42975 |
| 264.2008 | -4.23504 |
| 263.8522 | -4.0183 |
| 263.5046 | -3.78371 |
| 263.1579 | -3.53569 |
| 262.8121 | -3.27872 |
| 262.4672 | -3.01718 |
| 262.1232 | -2.75524 |
| 261.7801 | -2.49676 |
| 261.4379 | -2.24518 |
| 261.0966 | -2.00346 |
| 260.7562 | -1.77403 |
| 260.4167 | -1.55882 |
| 260.078 | -1.35919 |
| 259.7403 | -1.17602 |
| 259.4034 | -1.00972 |
| 259.0674 | -0.86027 |
| 258.7322 | -0.72731 |
| 258.3979 | -0.61017 |
| 258.0645 | -0.50797 |
| 257.732 | -0.41963 |
| 257.4003 | -0.34399 |
| 257.0694 | -0.27982 |
| 256.7394 | -0.22587 |
| 256.4103 | -0.18092 |
| 256.0819 | -0.1438 |
| 255.7545 | -0.11342 |

| | |
|---|---|
| 255.4278 | -0.08877 |
| 255.102 | -0.06894 |
| 254.7771 | -0.05313 |
| 254.4529 | -0.04063 |
| 254.1296 | -0.03083 |
| 253.8071 | -0.02322 |
| 253.4854 | -0.01735 |
| 253.1646 | -0.01287 |
| 252.8445 | -0.00947 |
| 252.5253 | -0.00691 |
| 252.2068 | -0.00501 |
| 251.8892 | -0.0036 |
| 251.5723 | -0.00257 |
| 251.2563 | -0.00182 |
| 250.941 | -0.00128 |
| 250.6266 | -0.00089 |
| 250.3129 | -0.00062 |
| 250 | -0.00042 |
| 249.6879 | -0.00029 |
| 249.3766 | -0.0002 |
| 249.066 | -0.00013 |
| 248.7562 | -8.7E-05 |
| 248.4472 | -5.8E-05 |
| 248.139 | -3.8E-05 |
| 247.8315 | -2.5E-05 |
| 247.5248 | -1.6E-05 |
| 247.2188 | -1.1E-05 |
| 246.9136 | -8E-06 |
| 246.6091 | -7E-06 |
| 246.3054 | -7E-06 |
| 246.0025 | -8E-06 |
| 245.7002 | -1.1E-05 |
| 245.3988 | -1.7E-05 |
| 245.098 | -2.5E-05 |
| 244.798 | -3.8E-05 |
| 244.4988 | -5.8E-05 |
| 244.2002 | -8.8E-05 |
| 243.9024 | -0.00013 |
| 243.6054 | -0.0002 |
| 243.309 | -0.00029 |
| 243.0134 | -0.00043 |
| 242.7184 | -0.00063 |
| 242.4242 | -0.00091 |
| 242.1308 | -0.00131 |

| | |
|---|---|
| 241.838 | -0.00187 |
| 241.5459 | -0.00265 |
| 241.2545 | -0.00372 |
| 240.9639 | -0.00519 |
| 240.6739 | -0.00718 |
| 240.3846 | -0.00985 |
| 240.096 | -0.01341 |
| 239.8082 | -0.01812 |
| 239.521 | -0.02429 |
| 239.2345 | -0.03232 |
| 238.9486 | -0.04267 |
| 238.6635 | -0.05591 |
| 238.379 | -0.07269 |
| 238.0952 | -0.09377 |
| 237.8121 | -0.12005 |
| 237.5297 | -0.1525 |
| 237.2479 | -0.19225 |
| 236.9668 | -0.2405 |
| 236.6864 | -0.29855 |
| 236.4066 | -0.36778 |
| 236.1275 | -0.44959 |
| 235.8491 | -0.54539 |
| 235.5713 | -0.65656 |
| 235.2941 | -0.78435 |
| 235.0176 | -0.92988 |
| 234.7418 | -1.09403 |
| 234.4666 | -1.27738 |
| 234.192 | -1.48016 |
| 233.9181 | -1.70218 |
| 233.6449 | -1.94276 |
| 233.3722 | -2.20073 |
| 233.1002 | -2.47434 |
| 232.8289 | -2.76131 |
| 232.5581 | -3.05884 |
| 232.288 | -3.36363 |
| 232.0186 | -3.67197 |
| 231.7497 | -3.97981 |
| 231.4815 | -4.28289 |
| 231.2139 | -4.57689 |
| 230.9469 | -4.85752 |
| 230.6805 | -5.12073 |
| 230.4147 | -5.3628 |
| 230.1496 | -5.58053 |
| 229.8851 | -5.77132 |

| | |
|---|---|
| 229.6211 | -5.93324 |
| 229.3578 | -6.06515 |
| 229.0951 | -6.16667 |
| 228.833 | -6.23817 |
| 228.5714 | -6.28071 |
| 228.3105 | -6.29596 |
| 228.0502 | -6.28605 |
| 227.7904 | -6.25343 |
| 227.5313 | -6.20071 |
| 227.2727 | -6.13047 |
| 227.0148 | -6.04512 |
| 226.7574 | -5.94676 |
| 226.5006 | -5.83701 |
| 226.2443 | -5.71702 |
| 225.9887 | -5.58732 |
| 225.7336 | -5.44793 |
| 225.4791 | -5.29833 |
| 225.2252 | -5.13759 |
| 224.9719 | -4.96449 |
| 224.7191 | -4.77765 |
| 224.4669 | -4.57571 |
| 224.2152 | -4.3575 |
| 223.9642 | -4.1222 |
| 223.7136 | -3.8695 |
| 223.4637 | -3.59969 |
| 223.2143 | -3.31375 |
| 222.9654 | -3.01339 |
| 222.7171 | -2.70103 |
| 222.4694 | -2.37978 |
| 222.2222 | -2.05329 |
| 221.9756 | -1.72568 |
| 221.7295 | -1.40135 |
| 221.4839 | -1.08479 |
| 221.2389 | -0.78047 |
| 220.9945 | -0.49258 |
| 220.7506 | -0.22493 |
| 220.5072 | 0.019225 |
| 220.2643 | 0.237284 |
| 220.022 | 0.427372 |
| 219.7802 | 0.588392 |
| 219.539 | 0.720019 |
| 219.2982 | 0.822677 |
| 219.0581 | 0.897473 |
| 218.8184 | 0.946117 |

| | |
|---|---|
| 218.5792 | 0.970811 |
| 218.3406 | 0.974136 |
| 218.1025 | 0.958932 |
| 217.8649 | 0.928174 |
| 217.6279 | 0.884863 |
| 217.3913 | 0.83192 |
| 217.1553 | 0.772107 |
| 216.9197 | 0.707954 |
| 216.6847 | 0.641709 |
| 216.4502 | 0.575308 |
| 216.2162 | 0.51036 |
| 215.9827 | 0.448148 |
| 215.7497 | 0.389642 |
| 215.5172 | 0.335521 |
| 215.2853 | 0.286205 |
| 215.0538 | 0.241891 |
| 214.8228 | 0.202591 |
| 214.5923 | 0.168166 |
| 214.3623 | 0.138365 |
| 214.1328 | 0.112859 |
| 213.9037 | 0.091265 |
| 213.6752 | 0.073176 |
| 213.4472 | 0.058177 |
| 213.2196 | 0.045864 |
| 212.9925 | 0.035853 |
| 212.766 | 0.02779 |
| 212.5399 | 0.021353 |
| 212.3142 | 0.016255 |
| 212.0891 | 0.012246 |
| 211.8644 | 0.009107 |
| 211.6402 | 0.00665 |
| 211.4165 | 0.004709 |
| 211.1932 | 0.00314 |
| 210.9705 | 0.001812 |
| 210.7482 | 0.000597 |
| 210.5263 | -0.00063 |
| 210.3049 | -0.00202 |
| 210.084 | -0.00374 |
| 209.8636 | -0.00599 |
| 209.6436 | -0.00904 |
| 209.4241 | -0.01325 |
| 209.205 | -0.01908 |
| 208.9864 | -0.02712 |
| 208.7683 | -0.03818 |

| | |
|---|---|
| 208.5506 | −0.05327 |
| 208.3333 | −0.07371 |
| 208.1165 | −0.10119 |
| 207.9002 | −0.13784 |
| 207.6843 | −0.1863 |
| 207.4689 | −0.24985 |
| 207.2539 | −0.3325 |
| 207.0393 | −0.43909 |
| 206.8252 | −0.57539 |
| 206.6116 | −0.7482 |
| 206.3983 | −0.96544 |
| 206.1856 | −1.23617 |
| 205.9732 | −1.57066 |
| 205.7613 | −1.98031 |
| 205.5498 | −2.47761 |
| 205.3388 | −3.07596 |
| 205.1282 | −3.78946 |
| 204.918 | −4.63257 |
| 204.7083 | −5.61974 |
| 204.499 | −6.76485 |
| 204.2901 | −8.0807 |
| 204.0816 | −9.5783 |
| 203.8736 | −11.2662 |
| 203.666 | −13.1496 |
| 203.4588 | −15.2298 |
| 203.252 | −17.5036 |
| 203.0457 | −19.9621 |
| 202.8398 | −22.5909 |
| 202.6342 | −25.3692 |
| 202.4292 | −28.2701 |
| 202.2245 | −31.2603 |
| 202.0202 | −34.3008 |
| 201.8163 | −37.3474 |
| 201.6129 | −40.3513 |
| 201.4099 | −43.261 |
| 201.2072 | −46.0229 |
| 201.005 | −48.5832 |
| 200.8032 | −50.8897 |
| 200.6018 | −52.8932 |
| 200.4008 | −54.5492 |
| 200.2002 | −55.8197 |

| Center Number | Atomic Number | Atomic Type | Coordinates (Angstroms) X | Y | Z |
|---|---|---|---|---|---|

---------------------------------------------------------------------

| | | | | | |
|---|---|---|---|---|---|
| 1 | 6 | 0 | -1.463236 | 1.090409 | 0.174834 |
| 2 | 7 | 0 | -0.521251 | 0.125669 | -0.136806 |
| 3 | 6 | 0 | -2.873406 | 0.625513 | 0.247015 |
| 4 | 1 | 0 | -3.576589 | 1.376357 | 0.592070 |
| 5 | 6 | 0 | -3.253523 | -0.587701 | -0.169113 |
| 6 | 1 | 0 | -4.305941 | -0.865254 | -0.179638 |
| 7 | 6 | 0 | -2.239467 | -1.577263 | -0.675292 |
| 8 | 1 | 0 | -2.540838 | -2.603005 | -0.431638 |
| 9 | 1 | 0 | -2.190590 | -1.520075 | -1.774044 |
| 10 | 6 | 0 | -0.868021 | -1.300675 | -0.058575 |
| 11 | 1 | 0 | -0.096855 | -1.868134 | -0.589500 |
| 12 | 1 | 0 | -0.847580 | -1.627881 | 0.988643 |
| 13 | 8 | 0 | -1.176007 | 2.275936 | 0.329728 |
| 14 | 6 | 0 | 0.875736 | 0.513948 | -0.117382 |
| 15 | 1 | 0 | 0.885023 | 1.563844 | 0.197091 |
| 16 | 6 | 0 | 1.642073 | 0.355654 | -1.445302 |
| 17 | 1 | 0 | 1.308925 | -0.551192 | -1.963587 |
| 18 | 1 | 0 | 1.499864 | 1.203610 | -2.119560 |
| 19 | 6 | 0 | 3.108925 | 0.190363 | -0.994568 |
| 20 | 1 | 0 | 3.693867 | -0.437015 | -1.675311 |
| 21 | 1 | 0 | 3.613696 | 1.164116 | -0.913225 |
| 22 | 7 | 0 | 2.959284 | -0.446145 | 0.309581 |
| 23 | 1 | 0 | 3.737963 | -0.785395 | 0.858329 |
| 24 | 6 | 0 | 1.734195 | -0.271363 | 0.895638 |
| 25 | 8 | 0 | 1.397653 | -0.655932 | 2.002450 |

## Data for calculation of optical rotation

Calculating GIAO nuclear magnetic shielding tensors.

SCF GIAO Magnetic shielding tensor (ppm):

1 C Isotropic = 35.4564 Anisotropy = 110.5718
XX= -39.0507 YX= 5.0785 ZX= 16.3207
XY= 21.7277 YY= 41.6783 ZY= -15.9333
XZ= 22.6121 YZ= -15.1856 ZZ= 103.7414
Eigenvalues: -44.4327 41.6309 109.1709
2 N Isotropic = 147.6901 Anisotropy = 99.1295
XX= 168.5144 YX= -56.9046 ZX= -2.8795
XY= -82.0969 YY= 99.9830 ZY= 3.2176
XZ= -19.4692 YZ= -5.4226 ZZ= 174.5730
Eigenvalues: 56.3628 172.9311 213.7765
3 C Isotropic = 68.1615 Anisotropy = 117.2454
XX= -13.3497 YX= 31.6535 ZX= 30.8479

XY= 17.4378 YY= 84.0586 ZY= -26.9430

XZ= 23.7026 YZ= -24.4095 ZZ= 133.7756

Eigenvalues: -25.9161 84.0755 146.3251

4 H Isotropic = 26.4002 Anisotropy = 3.7734

XX= 26.0259 YX= 1.5051 ZX= 0.1922

XY= 1.0014 YY= 28.1664 ZY= 0.8682

XZ= -0.2613 YZ= 0.9556 ZZ= 25.0083

Eigenvalues: 24.6367 25.6481 28.9158

5 C Isotropic = 57.5619 Anisotropy = 155.4752

XX= -42.8044 YX= 6.5495 ZX= 24.2115

XY= -2.3743 YY= 69.2447 ZY= -34.3959

XZ= 22.2019 YZ= -33.4111 ZZ= 146.2454

Eigenvalues: -45.9562 57.4299 161.2120

6 H Isotropic = 25.8798 Anisotropy = 3.1633

XX= 25.8978 YX= -0.9598 ZX= -0.4439

XY= -0.8603 YY= 27.1913 ZY= 1.1790

XZ= 0.3119 YZ= 1.1118 ZZ= 24.5501

Eigenvalues: 24.0815 25.5692 27.9886

7 C Isotropic = 163.3840 Anisotropy = 7.4865

XX= 165.9179 YX= -1.3229 ZX= -0.3311

XY= -5.5851 YY= 158.5143 ZY= -7.3699

XZ= 1.2352 YZ= 1.4647 ZZ= 165.7200

Eigenvalues: 156.4203 165.3568 168.3750

8 H Isotropic = 30.3128 Anisotropy = 8.3967

XX= 30.6184 YX= 2.7243 ZX= -0.0402

XY= 3.5836 YY= 34.0287 ZY= -0.1909

XZ= 0.1255 YZ= -0.1489 ZZ= 26.2912

Eigenvalues: 26.2834 28.7444 35.9106

9 H Isotropic = 29.8343 Anisotropy = 7.2916

XX= 28.9963 YX= 0.2427 ZX= 1.1820

XY= 0.1926 YY= 26.5394 ZY= 1.7455

XZ= 0.6810 YZ= 2.5153 ZZ= 33.9674

Eigenvalues: 25.9714 28.8361 34.6954

10 C Isotropic = 148.4115 Anisotropy = 27.2509

XX= 147.7478 YX= 5.5872 ZX= 5.9436

XY= 2.5491 YY= 165.6913 ZY= -2.5606

XZ= 9.9928 YZ= -1.9209 ZZ= 131.7954

Eigenvalues: 128.1435 150.5122 166.5787

11 H Isotropic = 29.3794 Anisotropy = 4.3771

XX= 32.2157 YX= 0.6903 ZX= -0.8795

XY= -0.3516 YY= 31.3850 ZY= 2.6874

XZ= -0.6882 YZ= 1.3014 ZZ= 24.5375

Eigenvalues: 23.9216 31.9191 32.2975
12 H Isotropic = 28.2627 Anisotropy = 6.8081
XX= 27.1020 YX= 2.4606 ZX= 2.0599
XY= 2.8621 YY= 28.2750 ZY= -4.3119
XZ= 2.4620 YZ= -3.5225 ZZ= 29.4111
Eigenvalues: 22.3297 29.6569 32.8014
13 O Isotropic = -53.0981 Anisotropy = 594.4255
XX= -155.1025 YX= -24.9523 ZX= 61.2508
XY= -25.1418 YY= -321.2072 ZY= -95.0684
XZ= 32.4201 YZ= -140.5149 ZZ= 317.0155
Eigenvalues: -343.7113 -158.7685 343.1856
14 C Isotropic = 138.0529 Anisotropy = 38.4680
XX= 156.5105 YX= 8.2599 ZX= 9.4745
XY= 1.1620 YY= 110.4918 ZY= -7.6003
XZ= 12.2996 YZ= -3.4537 ZZ= 147.1563
Eigenvalues: 108.8110 141.6494 163.6982
15 H Isotropic = 26.8120 Anisotropy = 5.8745
XX= 28.2445 YX= 0.1351 ZX= -1.4948
XY= -1.8128 YY= 28.8253 ZY= 2.5105
XZ= -1.6137 YZ= 2.8738 ZZ= 23.3662
Eigenvalues: 22.0514 27.6562 30.7283
16 C Isotropic = 161.1731 Anisotropy = 25.2165
XX= 177.7070 YX= -5.4674 ZX= 3.5038
XY= -0.0387 YY= 138.2497 ZY= 1.0126
XZ= -5.6534 YZ= -4.7754 ZZ= 167.5626
Eigenvalues: 137.9299 167.6053 177.9841
17 H Isotropic = 30.3251 Anisotropy = 6.0646
XX= 30.8767 YX= 2.0057 ZX= -0.1087
XY= 1.6725 YY= 29.1205 ZY= 3.6412
XZ= -0.1217 YZ= 4.0931 ZZ= 30.9782
Eigenvalues: 25.6237 30.9835 34.3682
18 H Isotropic = 30.1951 Anisotropy = 7.4658
XX= 28.6102 YX= -1.0420 ZX= -0.1212
XY= -0.5152 YY= 30.1538 ZY= -3.6448
XZ= -0.4760 YZ= -4.5354 ZZ= 31.8214
Eigenvalues: 26.5132 28.8999 35.1723
19 C Isotropic = 150.7131 Anisotropy = 33.1364
XX= 142.3833 YX= -0.5718 ZX= 1.1337
XY= 0.2167 YY= 142.9706 ZY= -12.9714
XZ= 0.2718 YZ= -13.7834 ZZ= 166.7855
Eigenvalues: 136.9658 142.3695 172.8041
20 H Isotropic = 29.1300 Anisotropy = 8.5535

XX= 30.3672 YX= -2.2346 ZX= -3.2985
XY= -2.6363 YY= 26.8661 ZY= 1.0426
XZ= -3.9002 YZ= 1.8945 ZZ= 30.1567
Eigenvalues: 25.5641 26.9935 34.8323
21 H Isotropic = 28.9299 Anisotropy = 8.9397
XX= 29.5464 YX= 4.4002 ZX= -0.3539
XY= 4.5722 YY= 30.5583 ZY= -1.8131
XZ= -0.2879 YZ= -1.9486 ZZ= 26.6849
Eigenvalues: 24.8718 27.0282 34.8897
22 N Isotropic = 148.1956 Anisotropy = 132.3750
XX= 234.8604 YX= -24.3075 ZX= 16.7474
XY= 28.3166 YY= 135.3018 ZY= 43.7762
XZ= -48.2180 YZ= 43.9549 ZZ= 74.4246
Eigenvalues: 50.2714 157.8698 236.4456
23 H Isotropic = 28.6400 Anisotropy = 12.0097
XX= 34.8998 YX= -3.8362 ZX= 5.3845
XY= -0.9357 YY= 25.0815 ZY= -2.0733
XZ= 1.1082 YZ= -1.5191 ZZ= 25.9387
Eigenvalues: 23.6607 25.6129 36.6465
24 C Isotropic = 29.0729 Anisotropy = 85.2901
XX= -30.8080 YX= 46.3469 ZX= -16.6883
XY= 33.3123 YY= 61.0797 ZY= 26.7824
XZ= -6.6921 YZ= 16.5962 ZZ= 56.9471
Eigenvalues: -48.9781 50.2639 85.9330
25 O Isotropic = -31.7369 Anisotropy = 588.4852
XX= -153.7492 YX= 112.6007 ZX= 142.3235
XY= 87.4510 YY= 214.1618 ZY= 250.2964
XZ= 112.5723 YZ= 196.2552 ZZ= -155.6234
Eigenvalues: -305.4388 -150.3586 360.5866

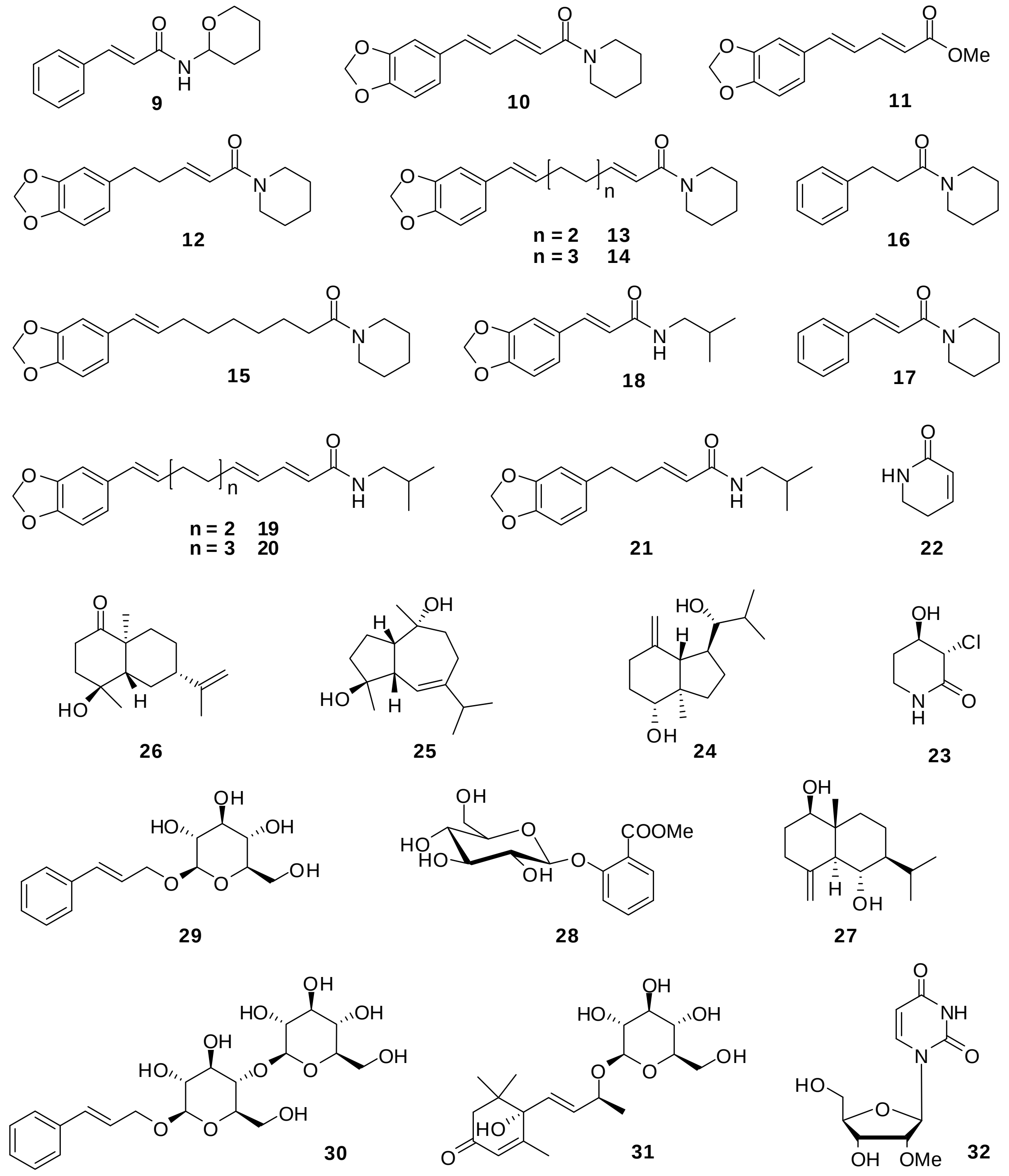


**Fig. S1** Chemical structures of known compounds (**9**–**32**) from *Piper retrofractum*

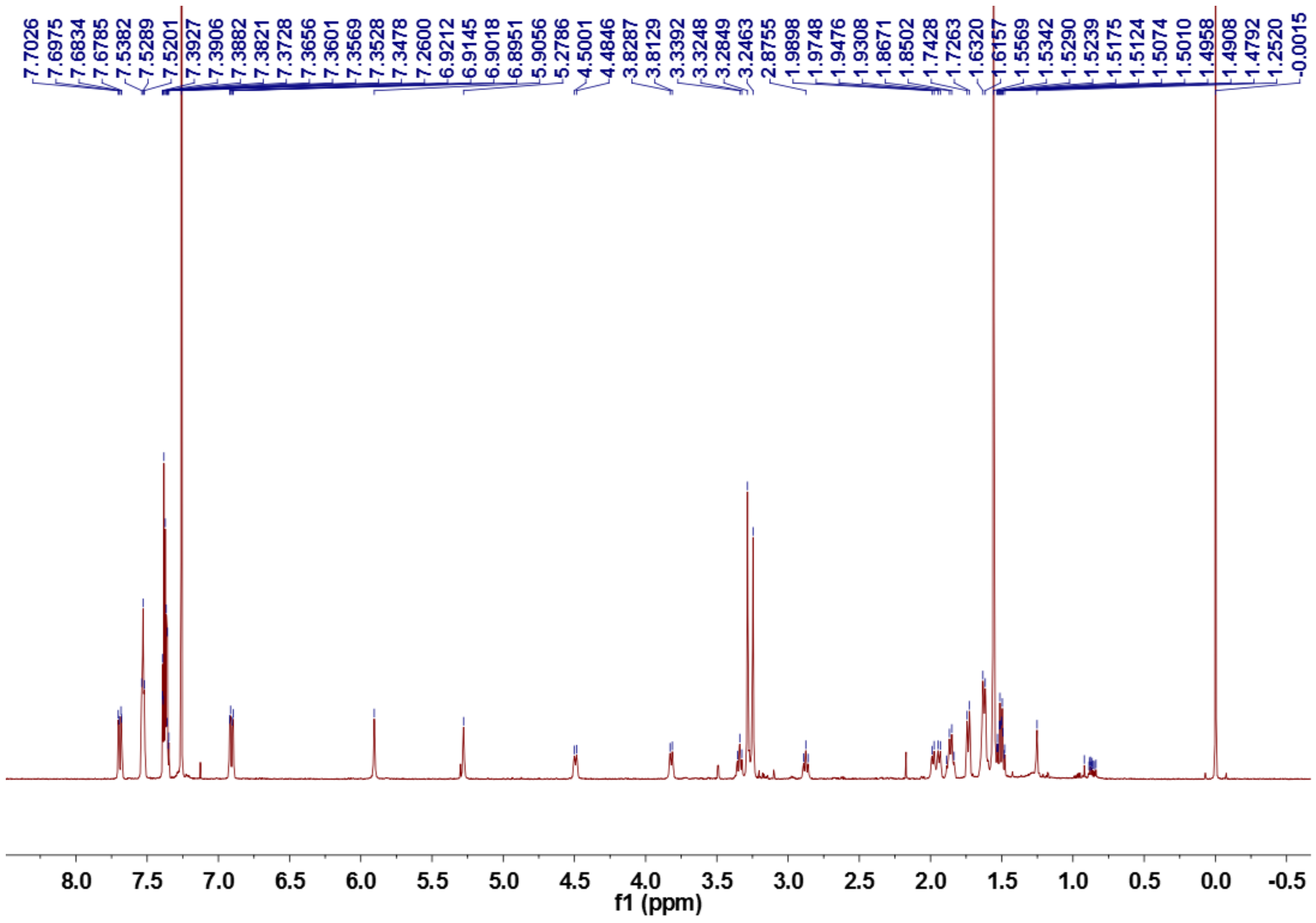

**Fig. S2** $^{1}$H NMR spectrum of compound **1** ($CDCl_3$, 800 MHz)

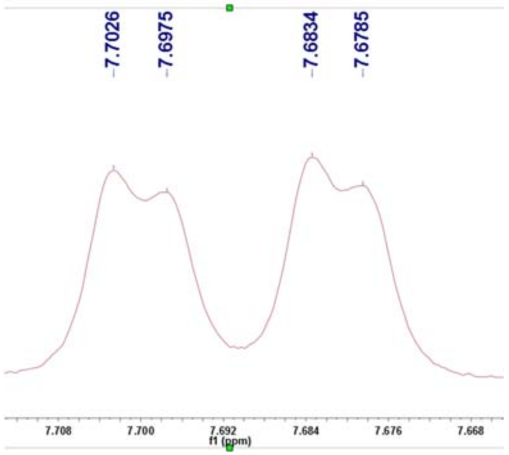

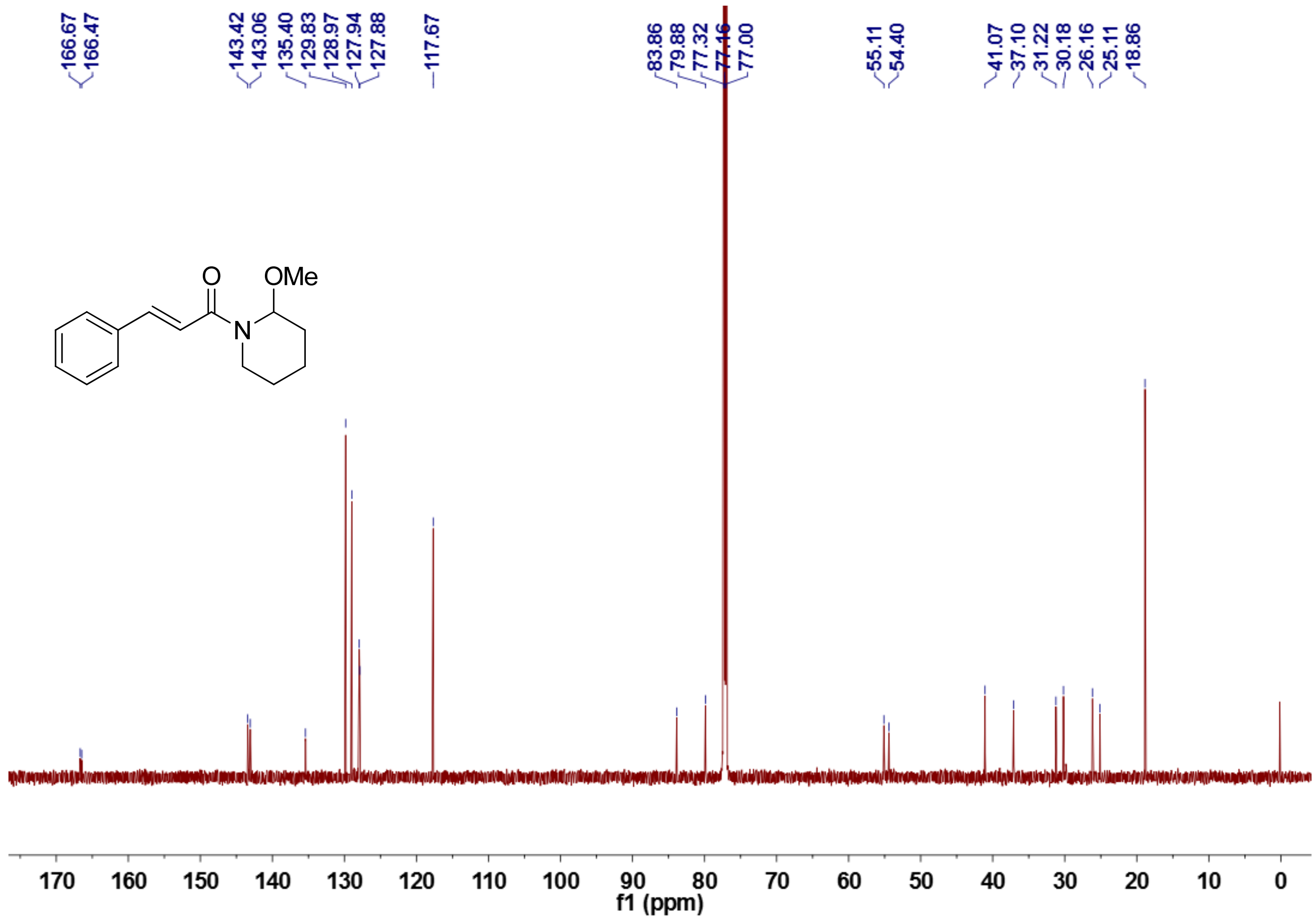


**Fig. S3** $^{13}C$ NMR spectrum of compound **1** ($CDCl_3$, 201 MHz)

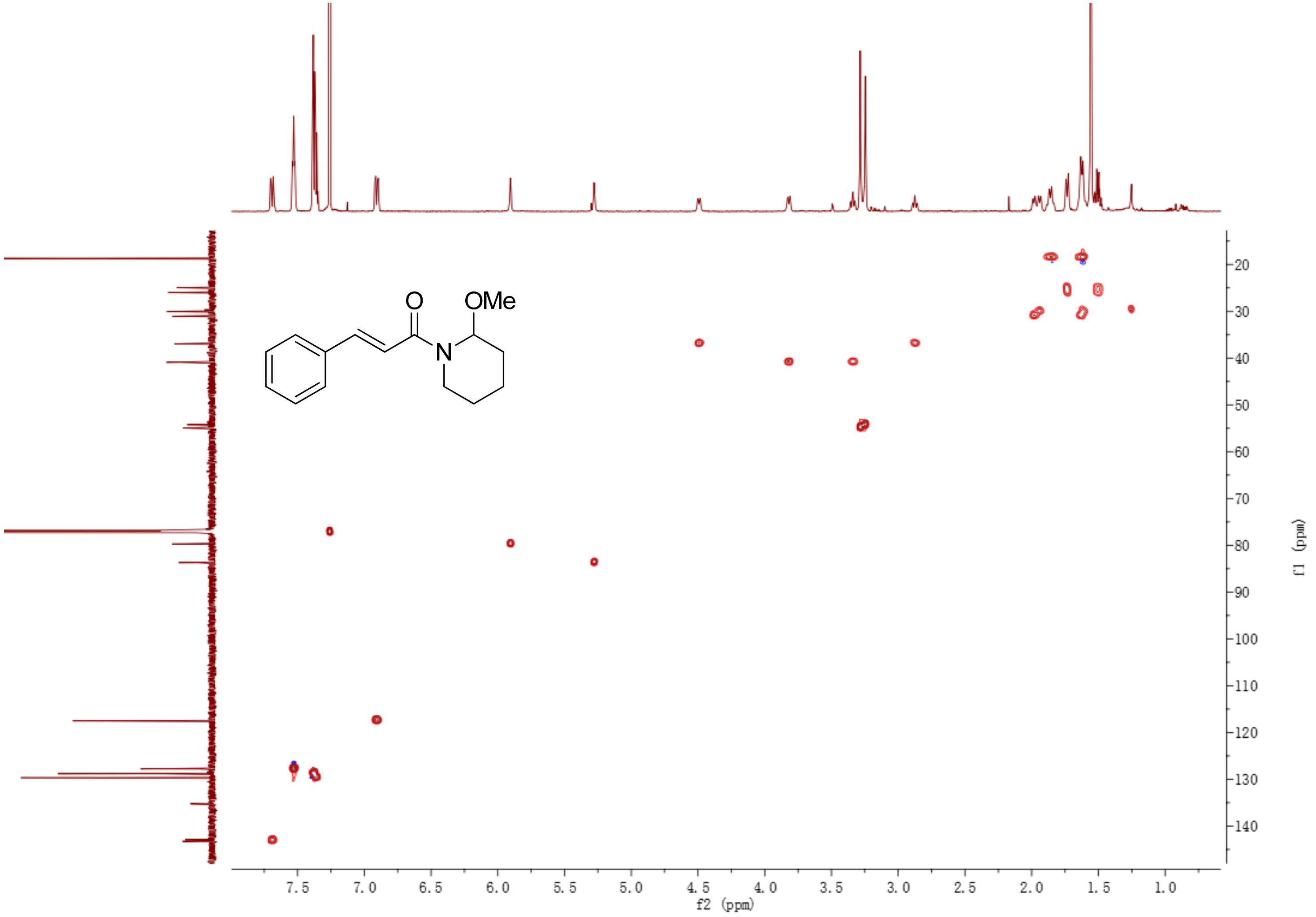


**Fig. S4** HSQC spectrum of compound **1**

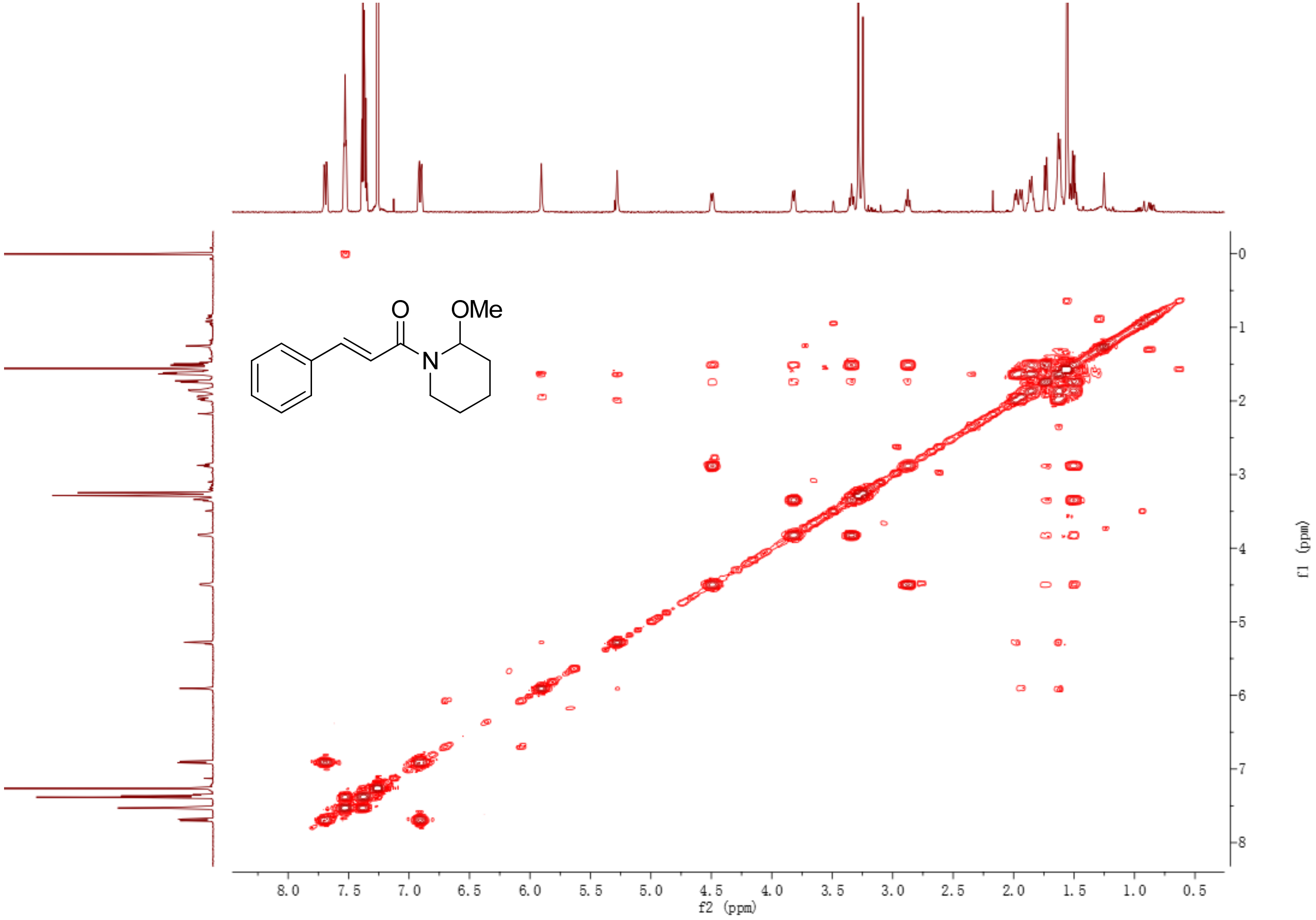


**Fig. S5** $^{1}H$–$^{1}H$ COSY spectrum of compound **1**

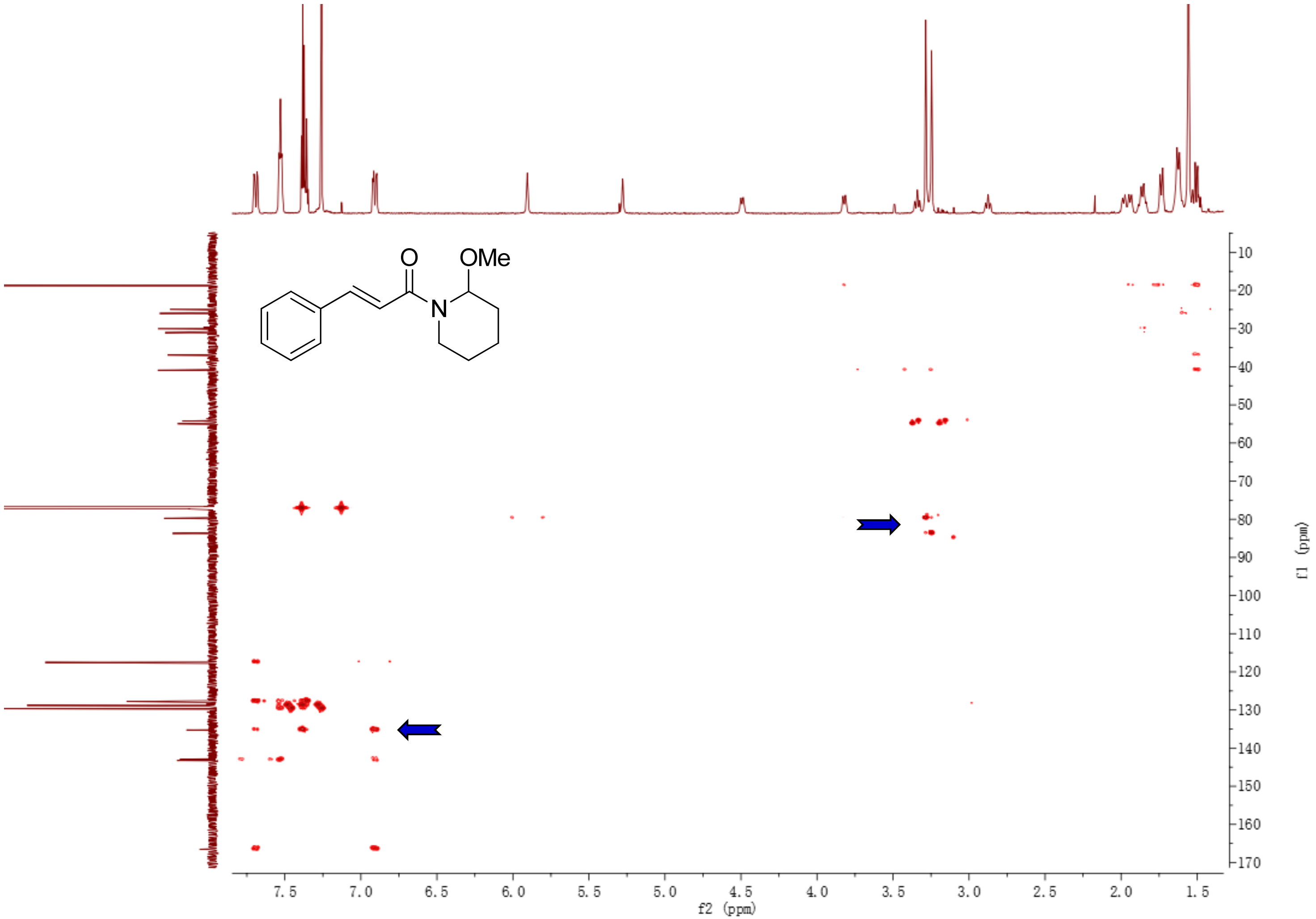


**Fig. S6** HMBC spectrum of compound **1**

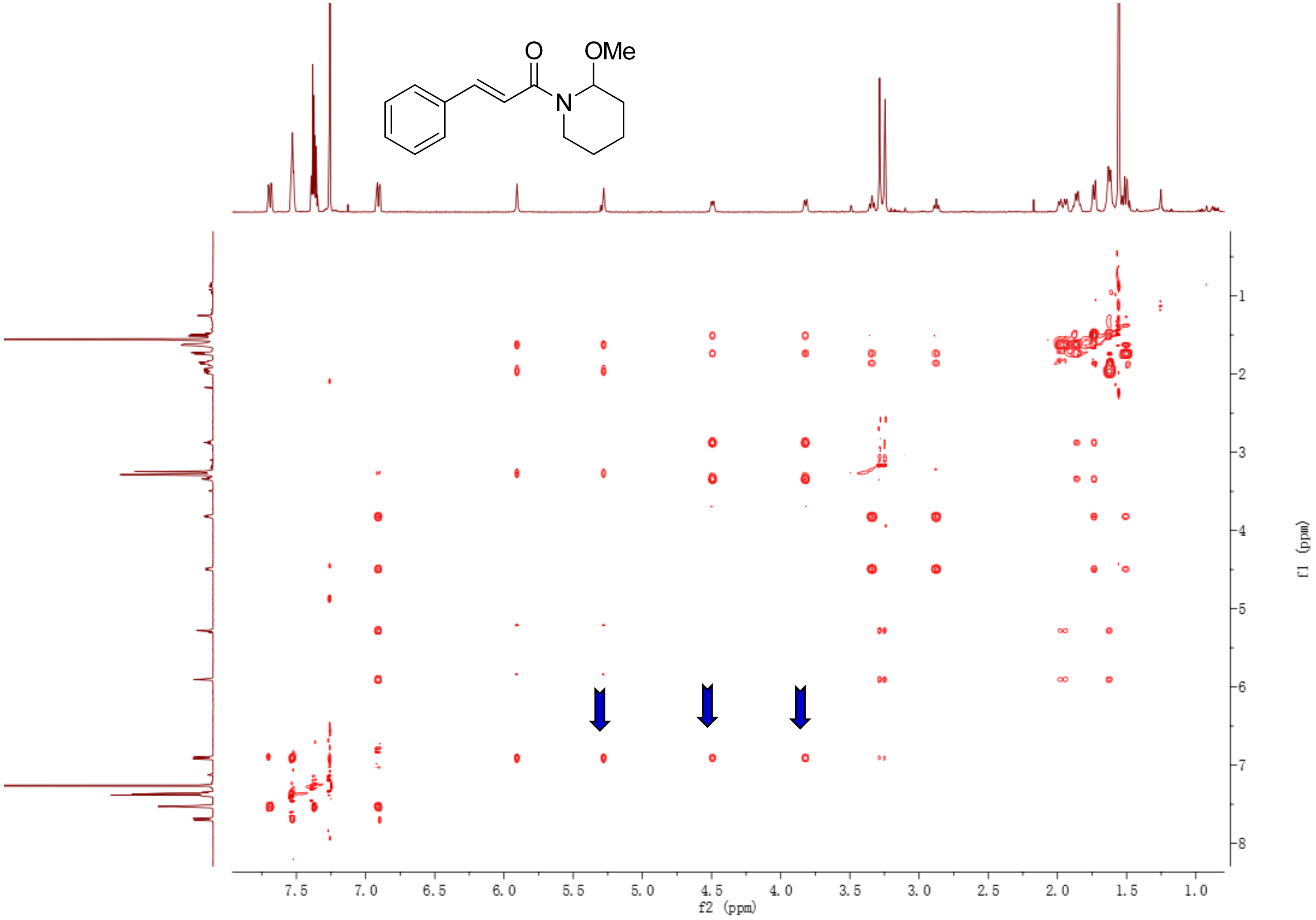


**Fig. S7** ROESY spectrum of compound **1**

**Elemental Composition Report** **Page 1**

**Single Mass Analysis**
Tolerance = 10.0 PPM / DBE: min = -10.0, max = 120.0
Selected filters: None

Monoisotopic Mass, Odd and Even Electron Ions
14 formula(e) evaluated with 1 results within limits (up to 51 closest results for each mass)
Elements Used:
C: 0-200 H: 0-400 N: 1-1 O: 1-3

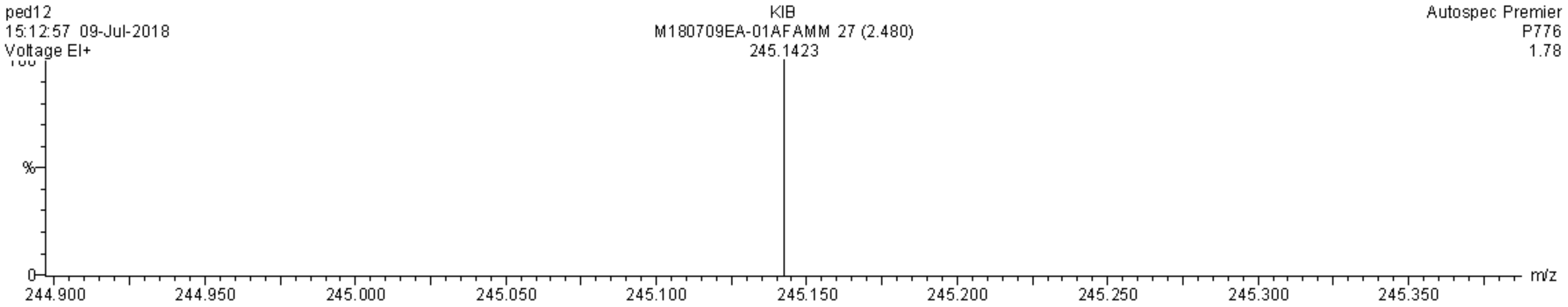


| | | | | | | |
|---|---|---|---|---|---|---|
| Minimum: | | | | -10.0 | | |
| Maximum: | | 200.0 | 10.0 | 120.0 | | |
| Mass | Calc. Mass | mDa | PPM | DBE | i-FIT | Formula |
| 245.1423 | 245.1416 | 0.7 | 2.9 | 7.0 | 5546025.5 | C15 H19 N O2 |

**Fig. S8** HREIMS spectrum of compound **1**

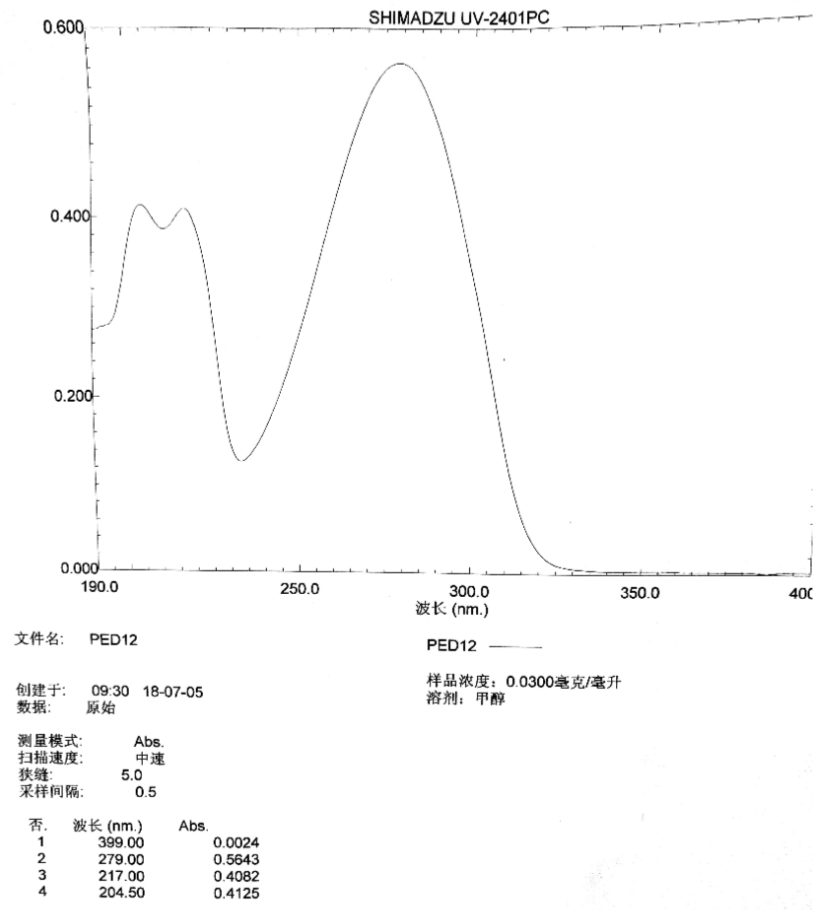


**Fig. S9** UV spectrum of compound **1**

Optical rotation measurement

Model : P-1020 (A060460638)

| No. | Sample | Mode | Data | Monitor<br>Blank | Temp.<br>Cell<br>Temp Point | Date<br>Comment<br>Sample Name | Light<br>Filter<br>Operator | Cycle Time<br>Integ Time |
|---|---|---|---|---|---|---|---|---|
| No.1 | 42 (1/3) | Sp.Rot | -16.6670 | -0.0025<br>0.0000 | 23.1<br>10.00<br>Cell | Tue Jul 03 20:29:03 2018<br>0.00150g/mL MeOH<br>PED12 | Na<br>589nm | 2 sec<br>2 sec |
| No.2 | 42 (2/3) | Sp.Rot | -14.6670 | -0.0022<br>0.0000 | 23.1<br>10.00<br>Cell | Tue Jul 03 20:29:08 2018<br>0.00150g/mL MeOH<br>PED12 | Na<br>589nm | 2 sec<br>2 sec |
| No.3 | 42 (3/3) | Sp.Rot | -14.6670 | -0.0022<br>0.0000 | 23.1<br>10.00<br>Cell | Tue Jul 03 20:29:13 2018<br>0.00150g/mL MeOH<br>PED12 | Na<br>589nm | 2 sec<br>2 sec |

**Fig. S10** OR spectrum of compound **1**

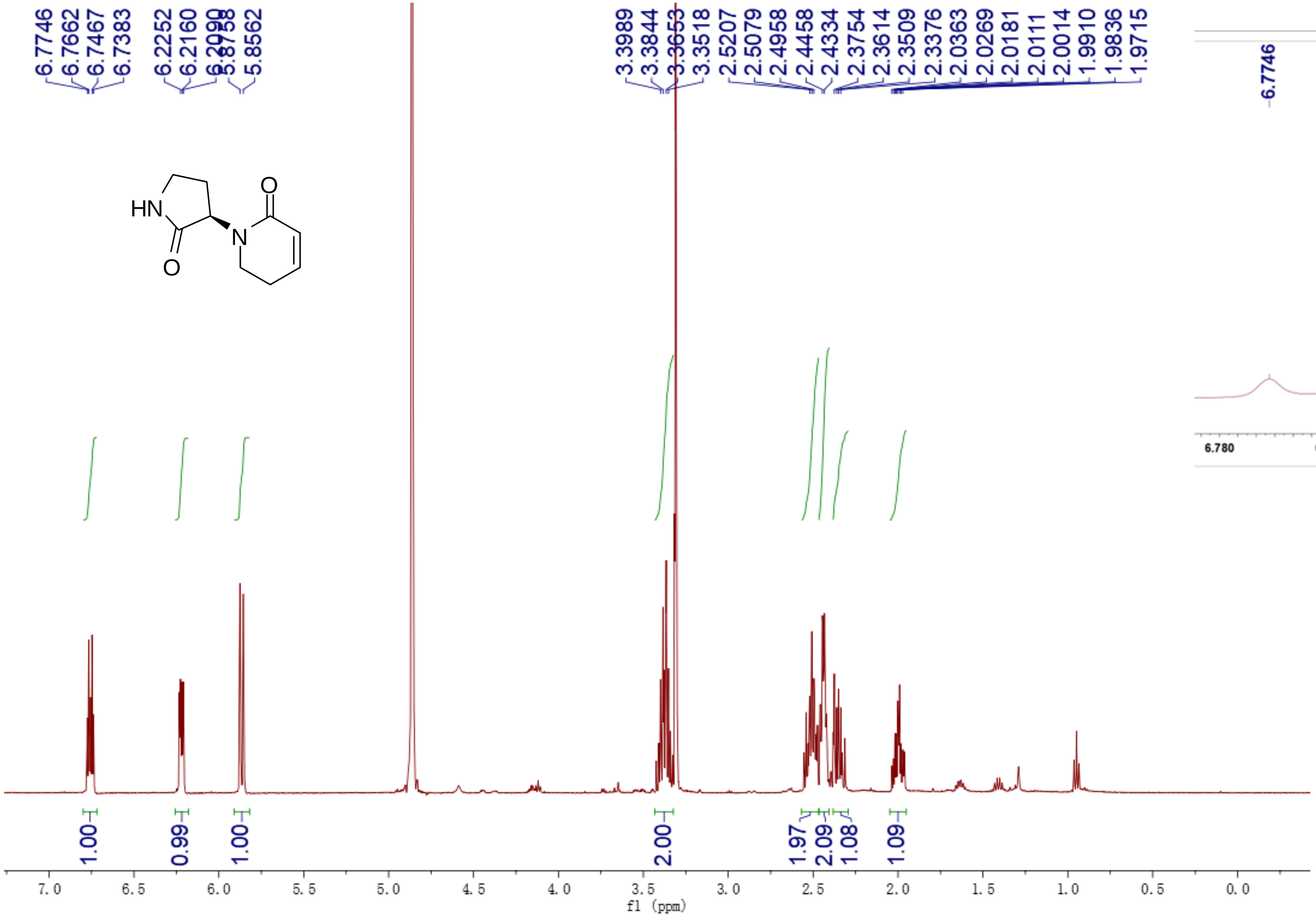

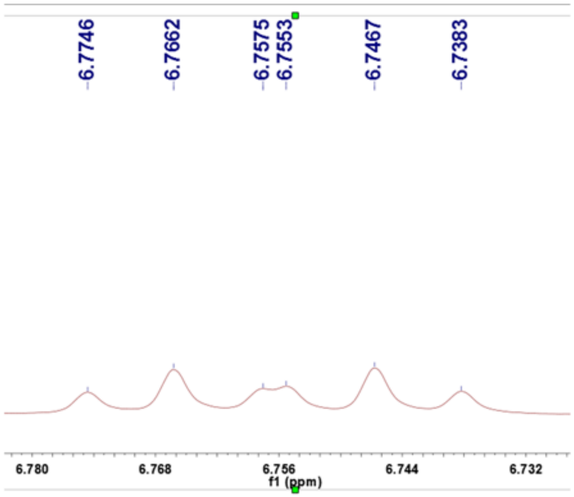


**Fig. S11** $^{1}$H NMR spectrum of compound **2** (methanol-$d_4$, 500 MHz)

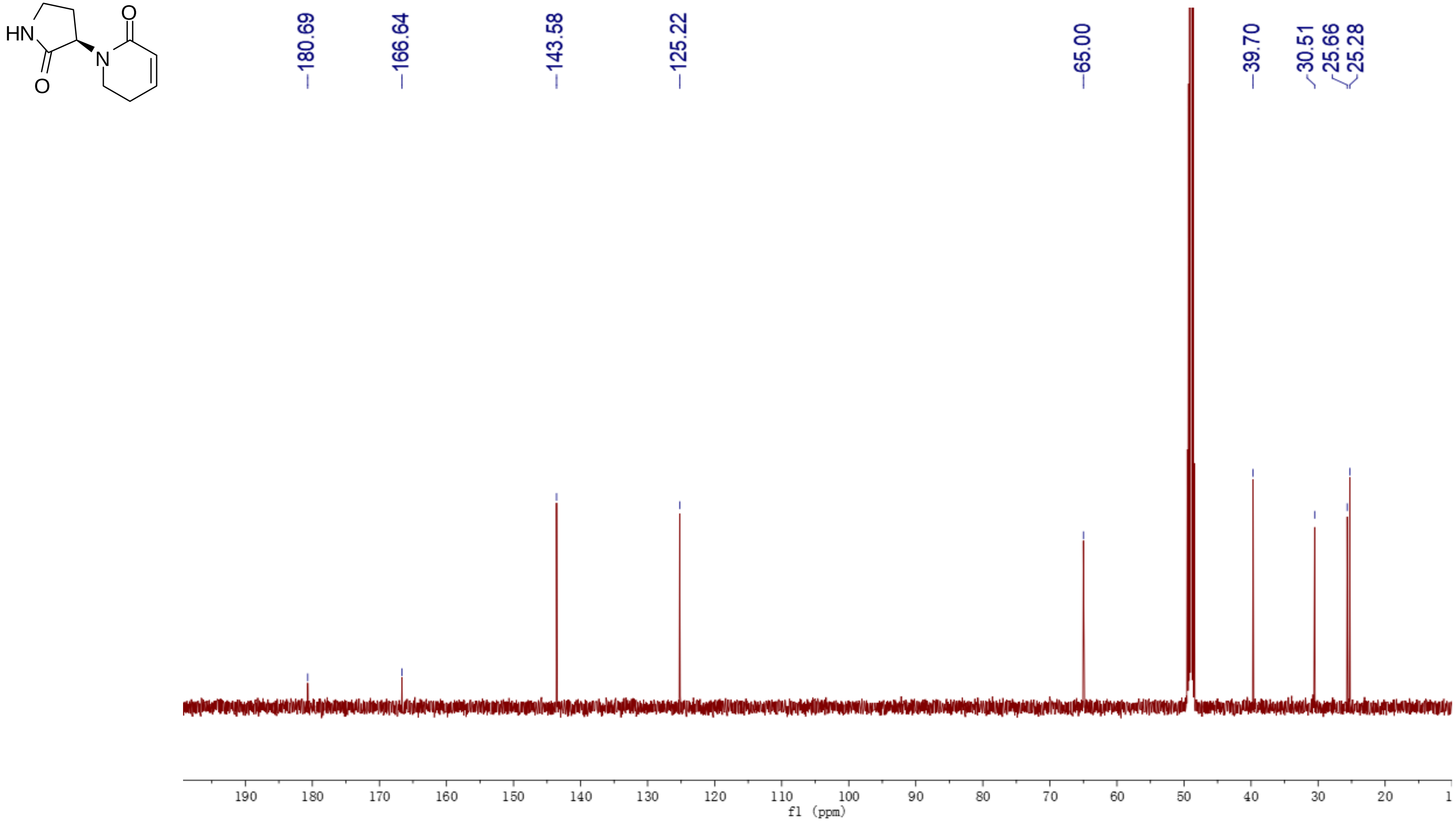


**Fig. S12** $^{13}$C NMR spectrum of compound **2** (methanol-$d_4$, 126 MHz)

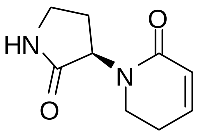


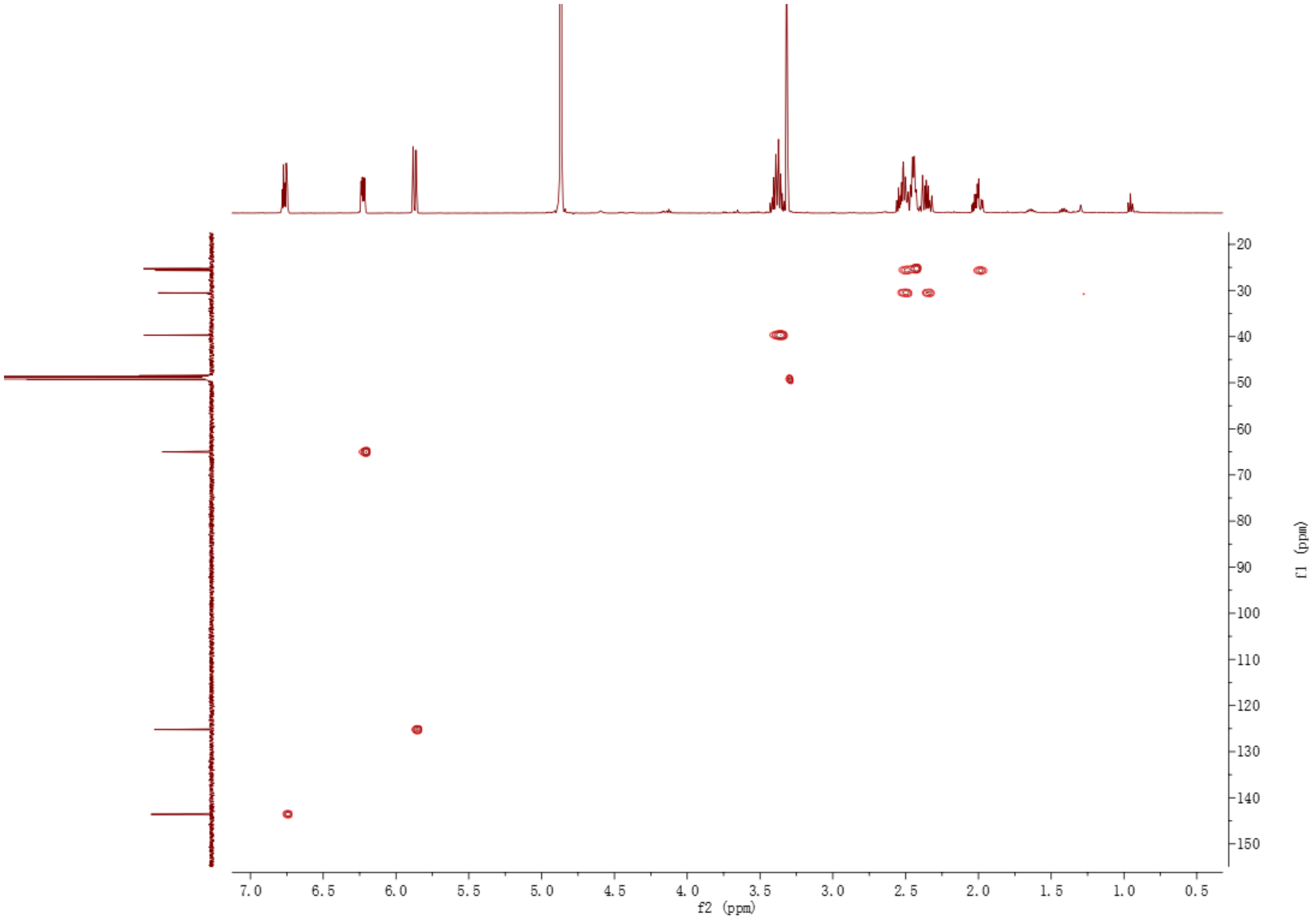


**Fig. S13** HSQC spectrum of compound **2**

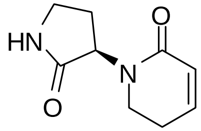

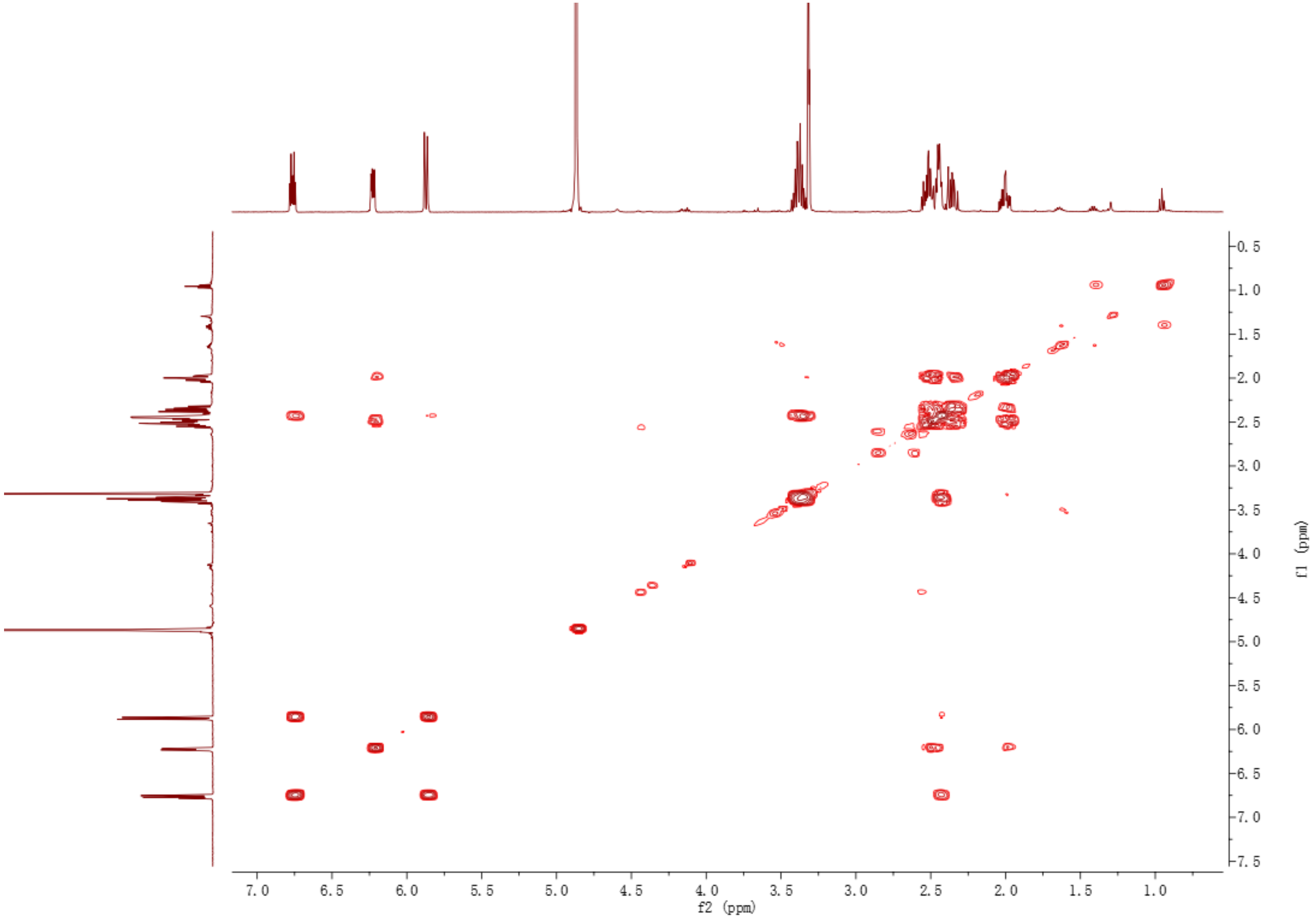


**Fig. S14** $^{1}H$–$^{1}H$ COSY spectrum of compound **2**

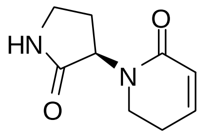


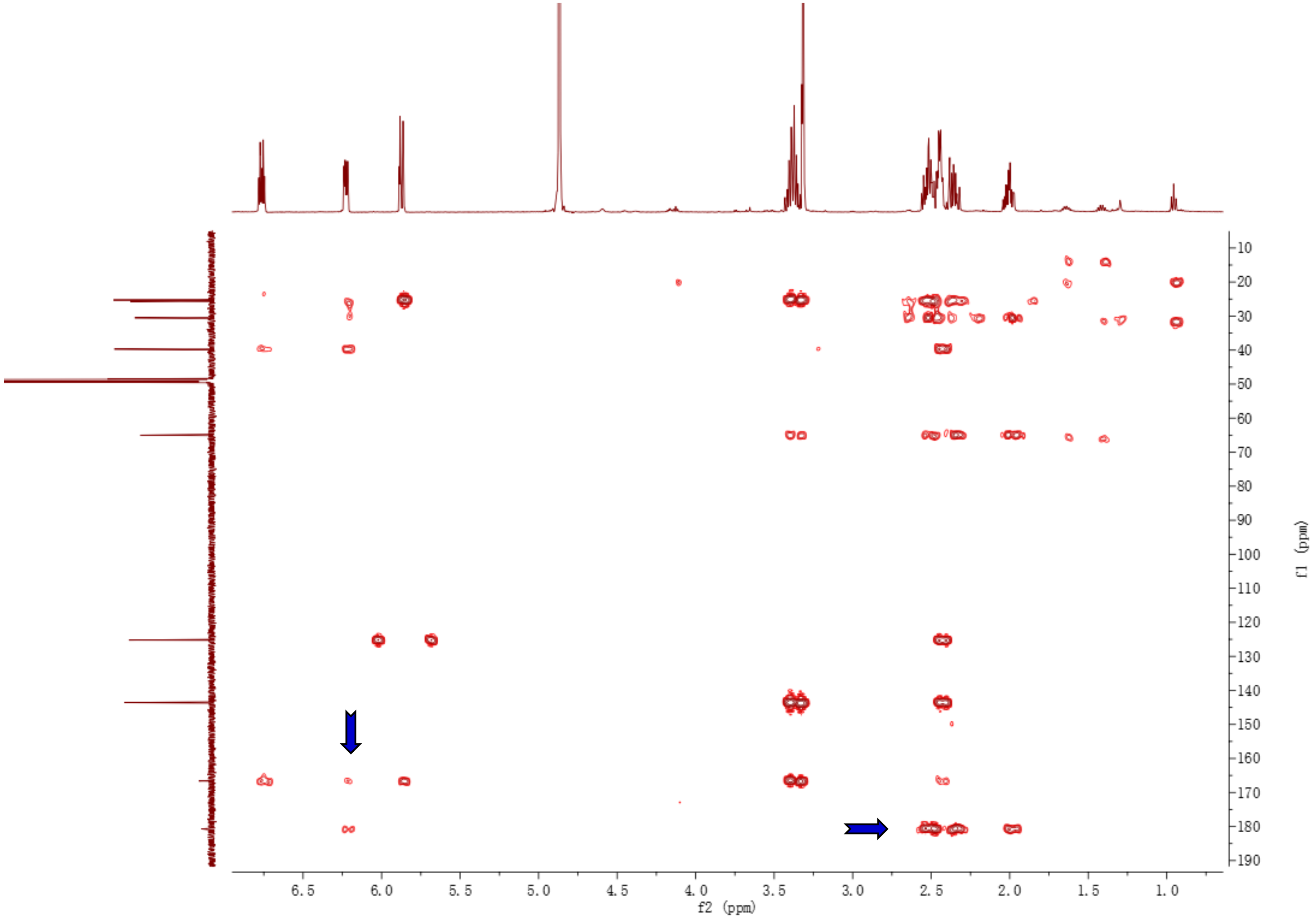


**Fig. S15** HMBC spectrum of compound **2**

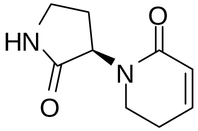


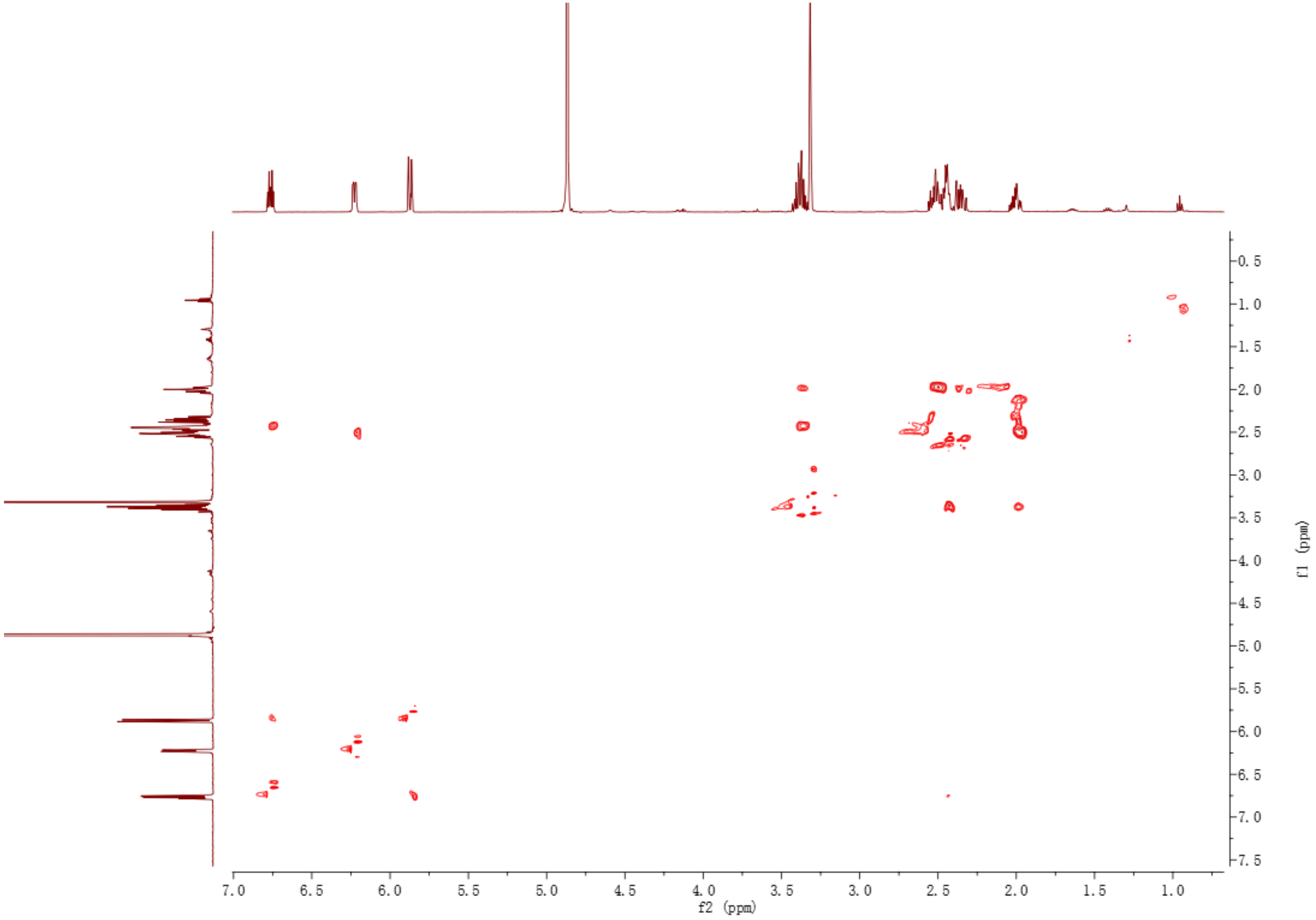


**Fig. S16** ROESY spectrum of compound **2**

## User Spectra

| Fragmentor Voltage | Collision Energy | Ionization Mode |
|---|---|---|
| 200 | 0 | ESI |

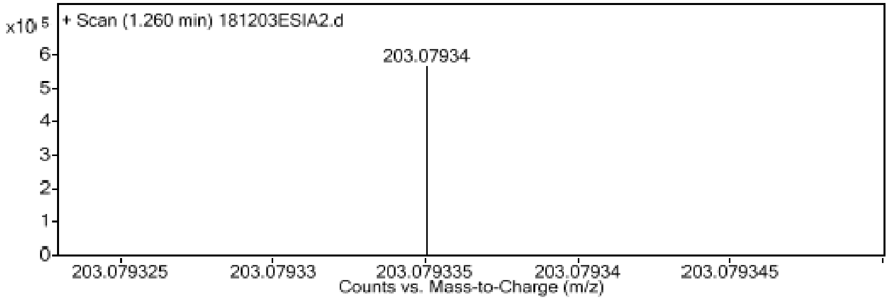


**Peak List**

| *m/z* | z | Abund | Formula | Ion |
|---|---|---|---|---|
| 106.02674 | 1 | 72286.21 | | |
| 121.05087 | 1 | 431707.63 | | |
| 142.02368 | 1 | 30008.54 | | |
| 203.07934 | 1 | 564116.06 | C9 H12 N2 Na O2 | M+ |
| 203.21044 | | 30214.92 | | |
| 204.08192 | 1 | 63958.44 | C9 H12 N2 Na O2 | M+ |
| 225.06087 | 1 | 36059.59 | | |
| 244.10559 | 1 | 172050.39 | | |
| 922.0098 | 1 | 159073.08 | | |
| 923.01264 | 1 | 29820.2 | | |

**Formula Calculator Element Limits**

| Element | Min | Max |
|---|---|---|
| C | 0 | 200 |
| H | 0 | 400 |
| O | 0 | 10 |
| Na | 1 | 1 |
| N | 2 | 2 |

**Formula Calculator Results**

| Formula | CalculatedMass | Mz | Diff.(mDa) | Diff. (ppm) | DBE |
|---|---|---|---|---|---|
| C9 H12 N2 Na O2 | 203.0797 | 203.0793 | 0.3 | 1.5 | 4.5 |

--- End Of Report ---

**Fig. S17** HRESIMS spectrum of compound **2**

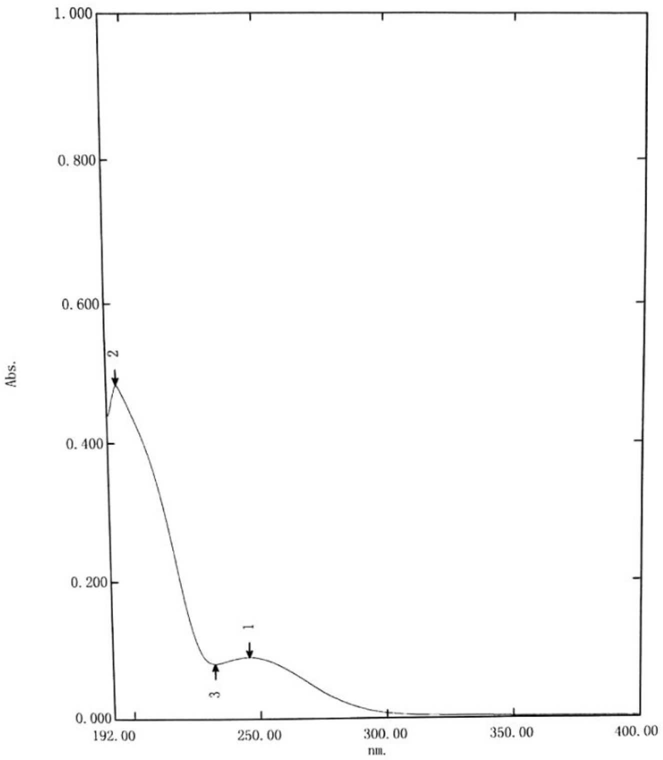


[测定属性]
波长范围(nm): 192.00 到 400.00
扫描速度: 中速
采样间隔: 0.2
自动采样间隔: 启用
扫描模式: 单个

[仪器属性]
仪器类型: UV-2700 系列
测定方式: 吸收值
狭缝宽: 5.0 nm
积分时间: 0.1 秒
光源转换波长: 323.0 nm
检测器单元: 直接
S/R 转换: 标准
阶梯校正: OFF

[附件属性]
附件: 无

[数据处理参数]
阈值: 0.0010000
点: 4
内插: 停用
平均: 停用

[样品准备属性]
重量: 3.5mg
体积: 1.5ml
稀释: 30倍
光程长: 10 mm
附加信息: 20181207 09:11:19
ped92 CH3OH

| No. | P/V | 波长(nm) | Abs. | 描述 |
|---|---|---|---|---|
| 1 | | 245.80 | 0.090 | |
| 2 | | 195.80 | 0.484 | |
| 3 | | 232.20 | 0.080 | |

**Fig. S18** UV spectrum of compound **2**

## Rudolph Research Analytical

This sample was measured on an Autopol VI, Serial #91058
Manufactured by Rudolph Research Analytical, Hackettstown, NJ, USA.

Measurement Date : Friday, 07-DEC-2018

Set Temperature : OFF

Time Delay : Disabled

Delay between Measurement : Disabled

| n | Average | Std.Dev. | % RSD | Maximum | Minimum |
|---|---|---|---|---|---|
| 5 | -15.63 | 0.15 | -0.95 | -15.45 | -15.84 |

| S.No | Sample ID | Time | Result | Scale | OR °Arc | WLG.nm | Lg.mm | Conc.g/100ml | Temp. |
|---|---|---|---|---|---|---|---|---|---|
| 1 | ped92 | 09:26:43 AM | -15.84 | SR | -0.0369 | 589 | 100.00 | 0.233 | 19.7 |
| 2 | ped92 | 09:26:51 AM | -15.71 | SR | -0.0366 | 589 | 100.00 | 0.233 | 19.7 |
| 3 | ped92 | 09:26:59 AM | -15.62 | SR | -0.0364 | 589 | 100.00 | 0.233 | 19.7 |
| 4 | ped92 | 09:27:07 AM | -15.54 | SR | -0.0362 | 589 | 100.00 | 0.233 | 19.6 |
| 5 | ped92 | 09:27:15 AM | -15.45 | SR | -0.0360 | 589 | 100.00 | 0.233 | 19.6 |

**Fig. S19** OR spectrum of compound **2**

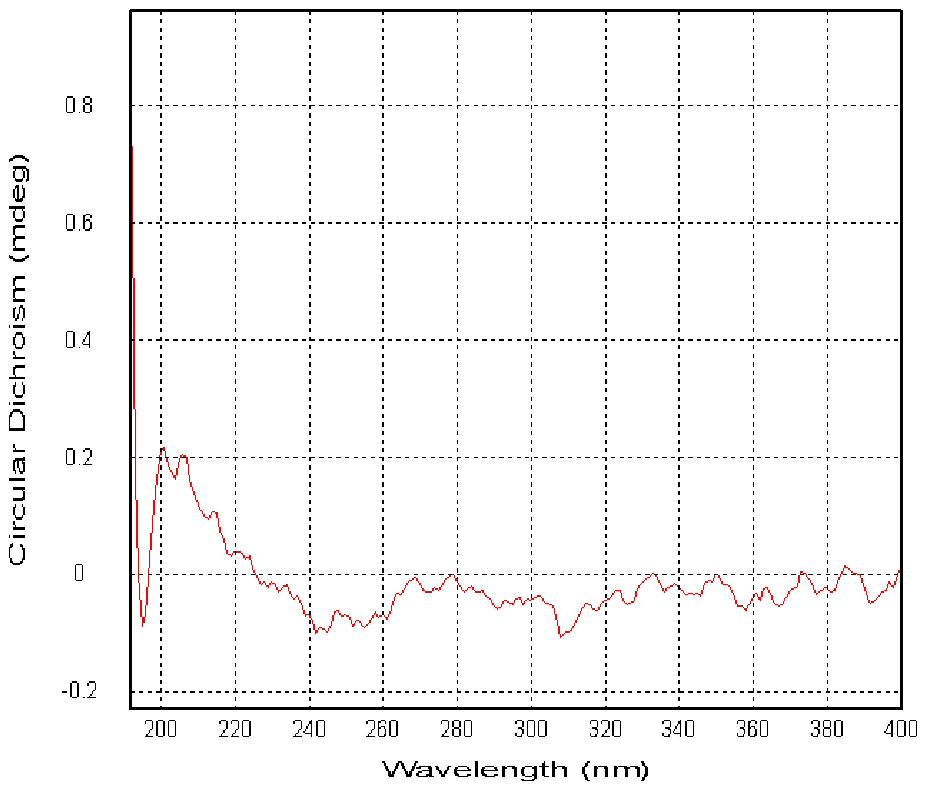


**Fig. S20** ECD spectrum of compound **2**

File: ped9200459.dsx
ProBinaryX
Attributes :
- Time Stamp :Mon Dec 10 16:30:49 2018

- File ID : {CF4AEC8B-3661-4ef3-9E14-1B942246CE63}
- Is CFR Compliant : false
- Original data has not been modified.

Remarks:
- User: CD
- Date: 2018/12/10
- Instrument: 0547
- DetectorType: LAAPD
- DichOS Calibration Correction Curve: 0547/2
- HV (CDDC channel): 487.793 v
- Time per point: 0.25 s
- Description: ped92
- Concentration: 0.144 mg/ml $CH_3OH$
- Pathlength: 1 mm
- Temperature: ---- C

Settings:
- Time-per-point: 0.25s (25us x 10000)
- SE
- Wavelength: 192nm - 400nm
- Step Size: 1nm
- Bandwidth: 1nm
- 3 repeats in set.
- -iter option selected

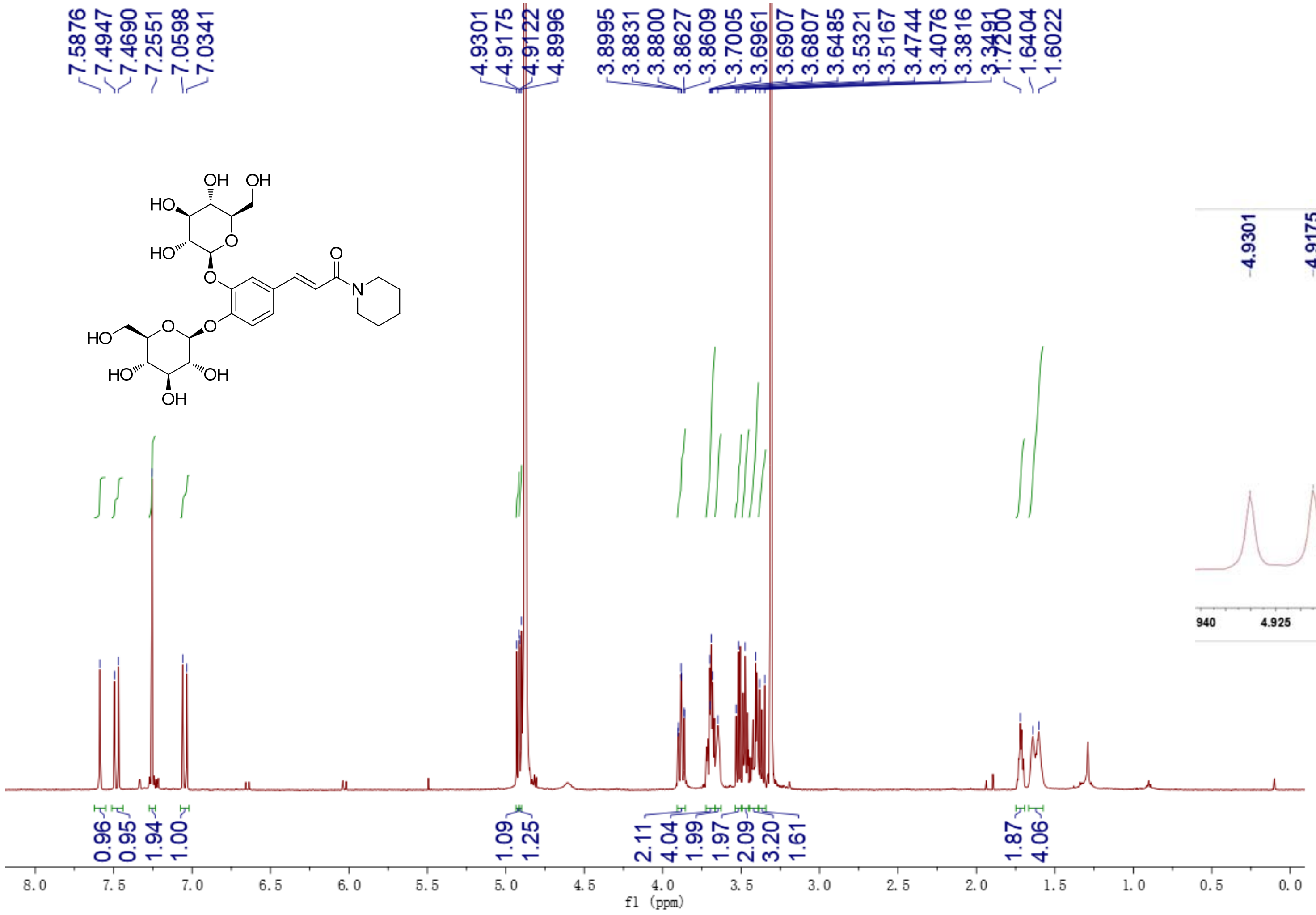


**Fig. S21** $^1$H NMR spectrum of compound **3** (methanol-$d_4$, 600 MHz)

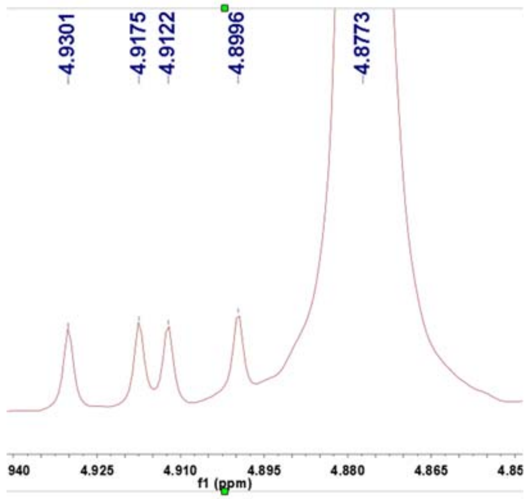

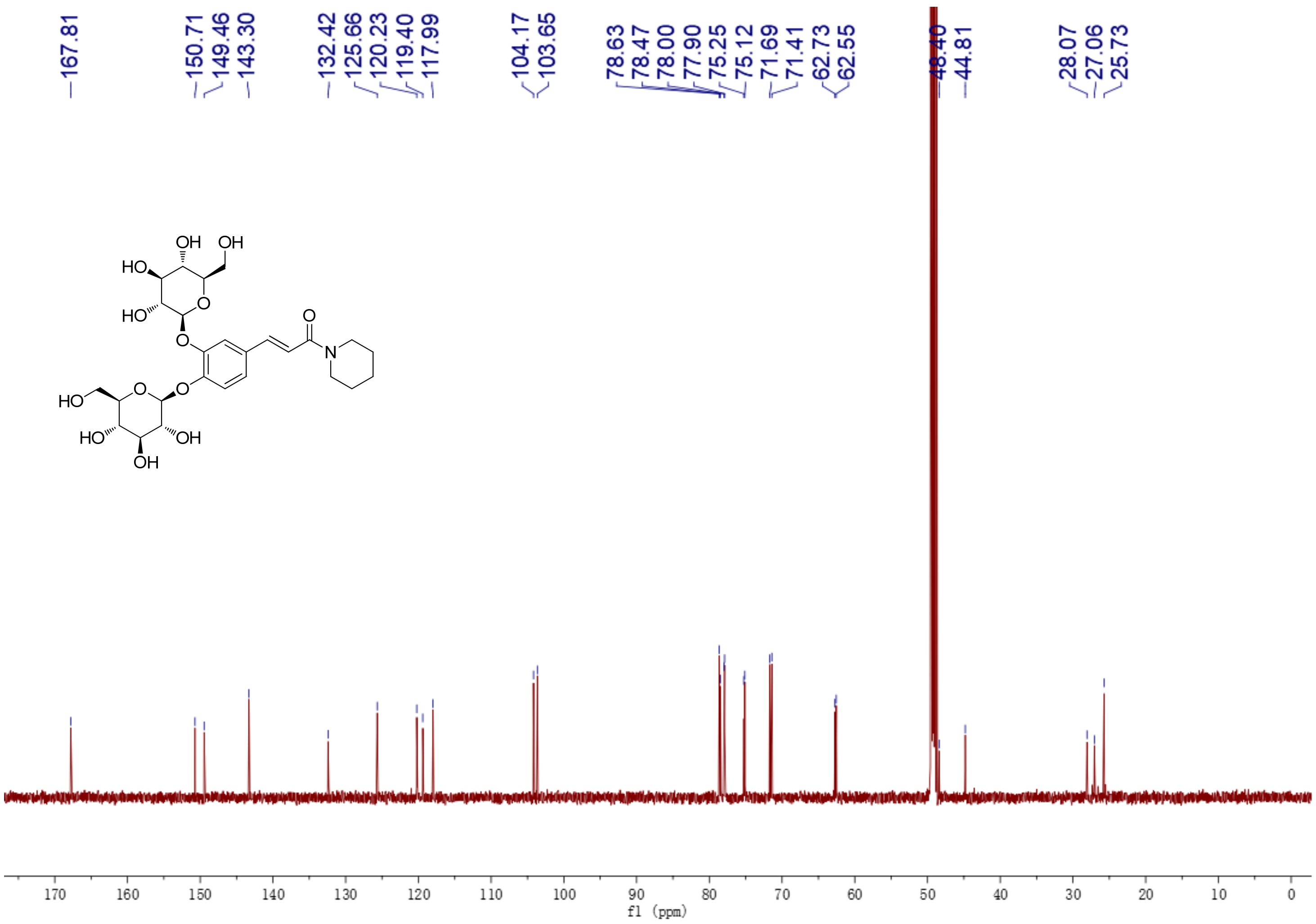


**Fig. S22** $^{13}C$ NMR spectrum of compound **3** (methanol-$d_4$, 151 MHz)

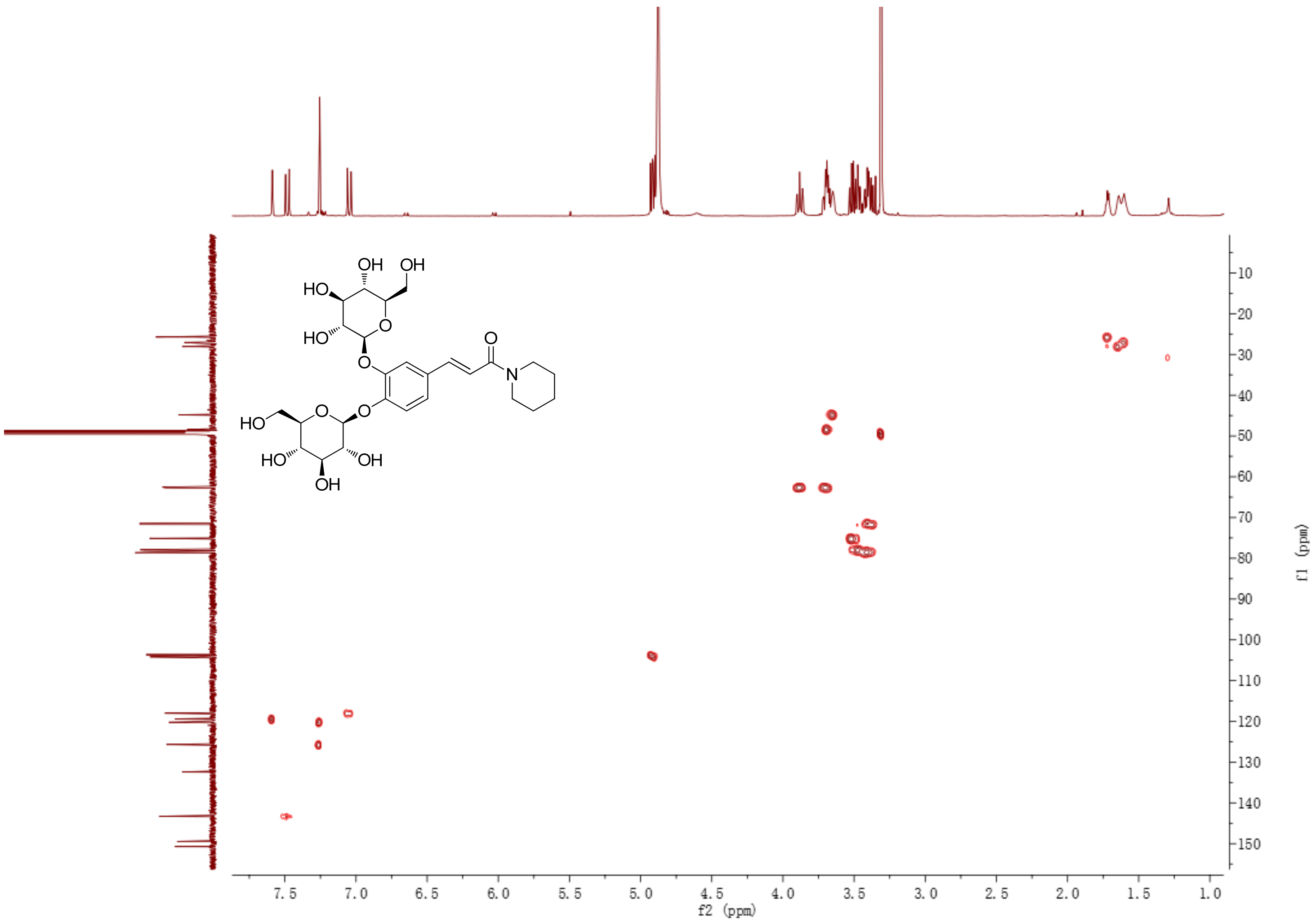


**Fig. S23** HSQC spectrum of compound **3**

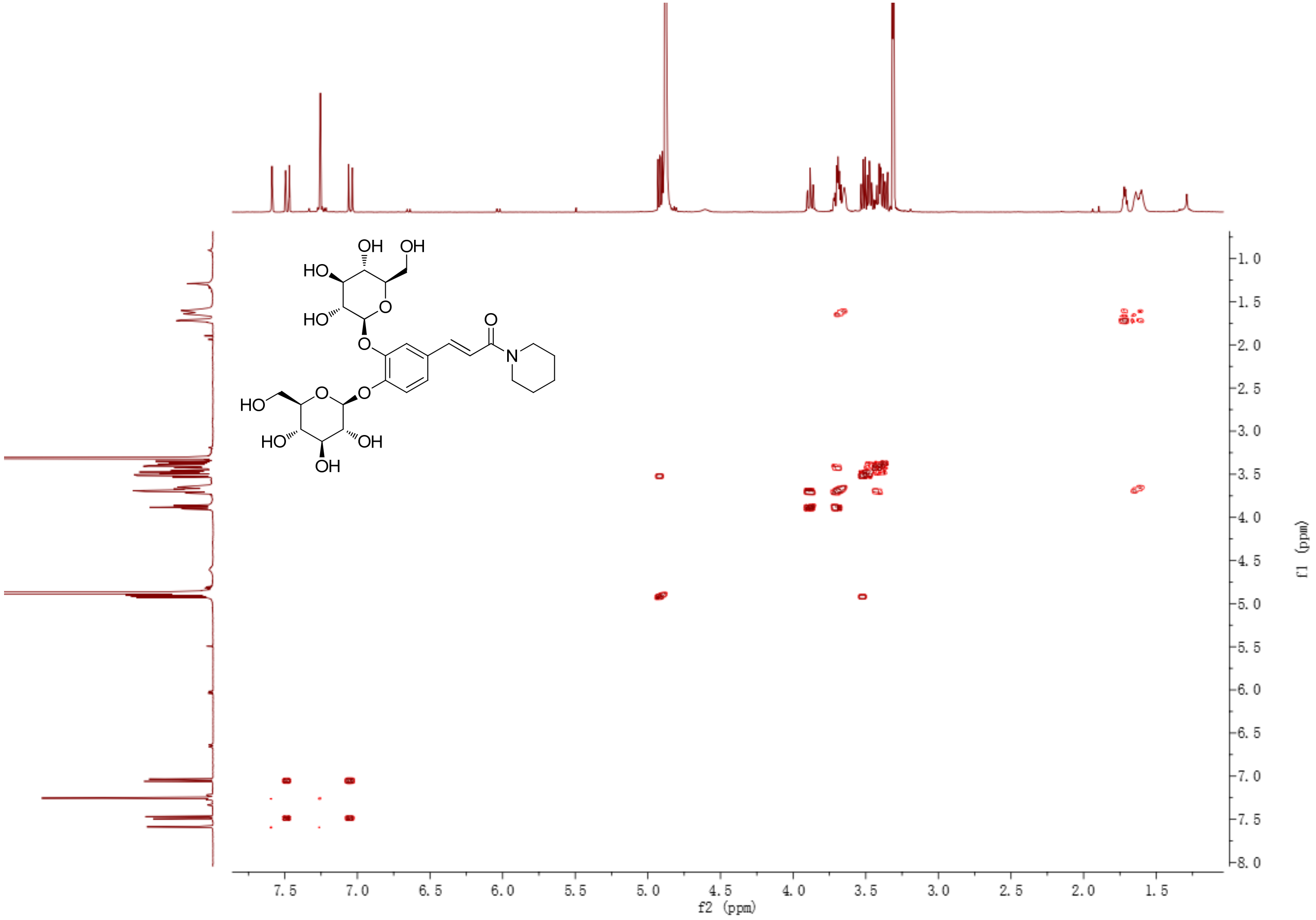


**Fig. S24** $^{1}$H–$^{1}$H COSY spectrum of compound **3**

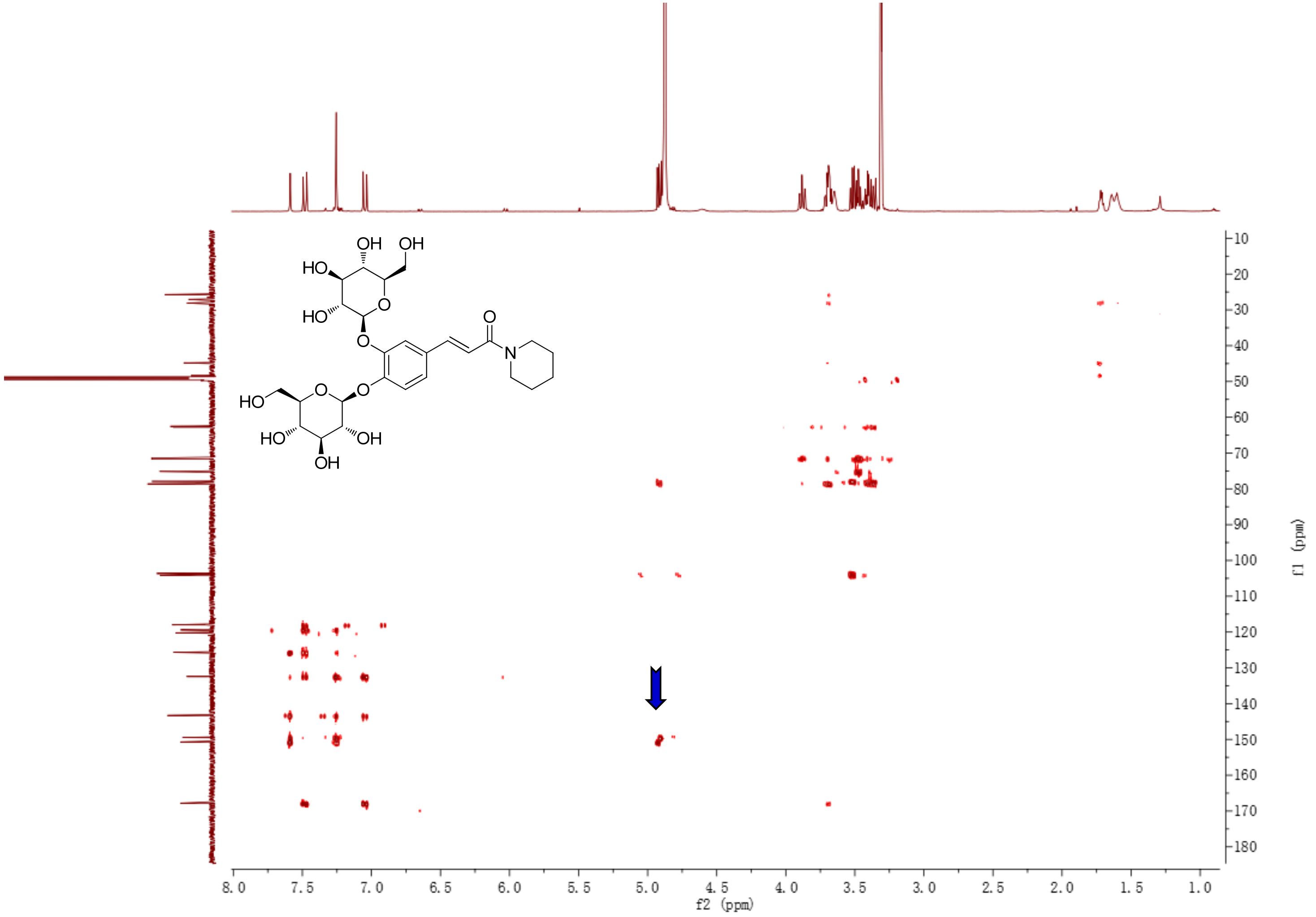


**Fig. S25** HMBC spectrum of compound **3**

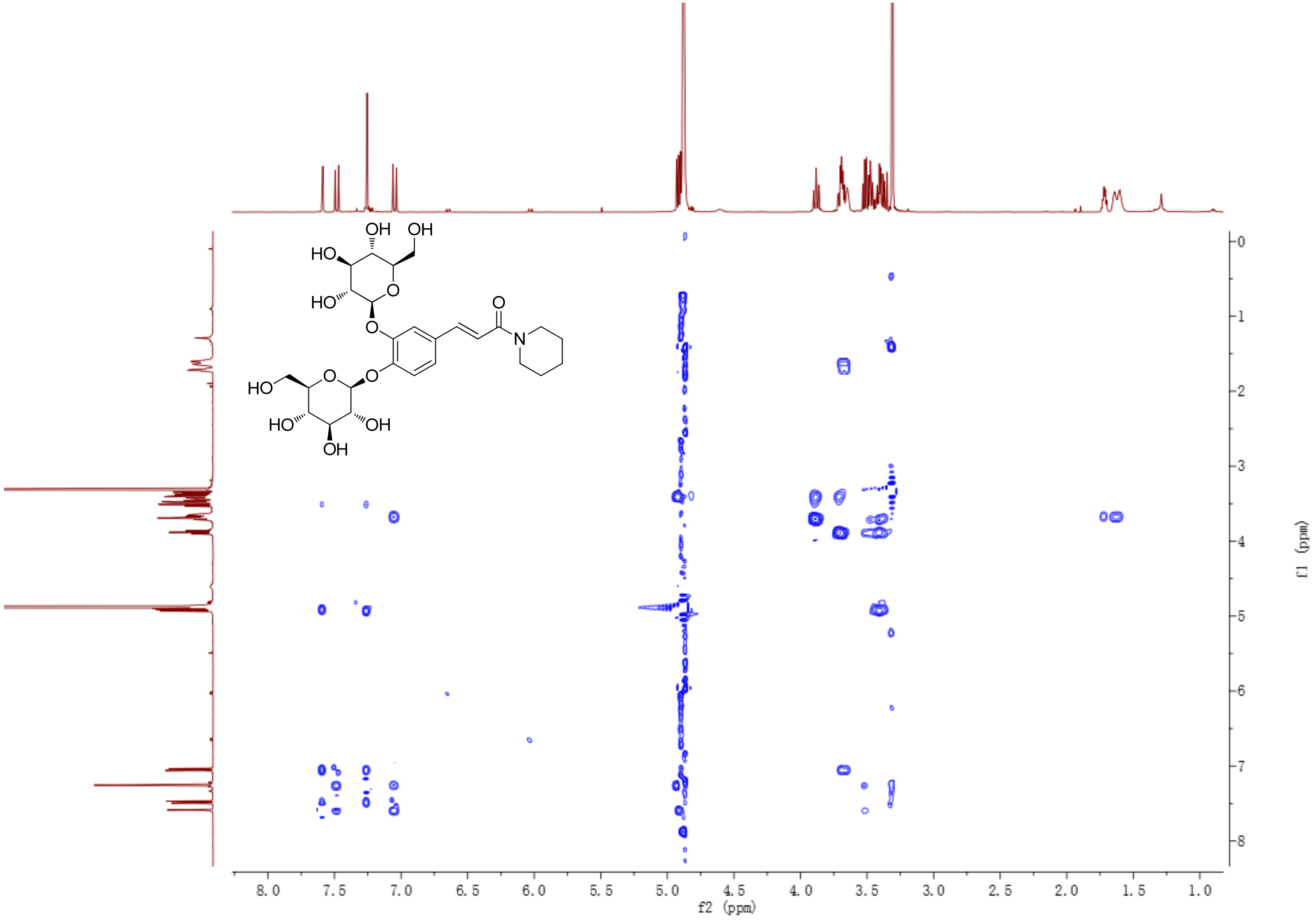


**Fig. S26** ROESY spectrum of compound **3**

## User Spectra

| Fragmentor Voltage | Collision Energy | Ionization Mode |
|---|---|---|
| 200 | 0 | ESI |

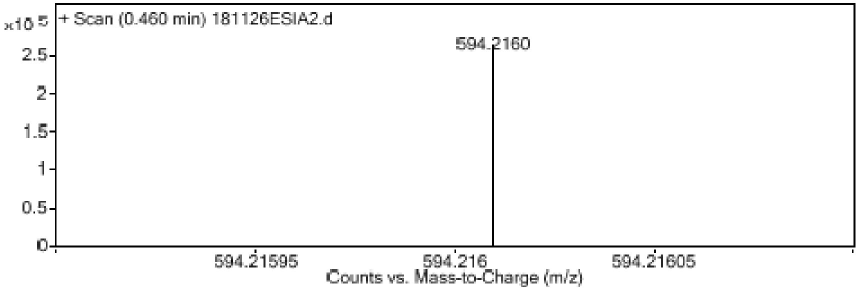


Peak List

| *m/z* | z | Abund | Formula | Ion |
|---|---|---|---|---|
| 105.0428 | | 45083.37 | | |
| 121.0509 | 1 | 237039.61 | | |
| 284.3308 | 1 | 32395.39 | | |
| 312.3622 | 1 | 25614.01 | | |
| 594.216 | 1 | 263344.81 | C26 H37 N Na O13 | M+ |
| 595.2191 | 1 | 73951.8 | C26 H37 N Na O13 | M+ |
| 610.1886 | 1 | 24433.31 | | |
| 692.2132 | 1 | 46007.93 | | |
| 922.0098 | 1 | 102349.88 | | |
| 1165.4406 | 1 | 22666.94 | | |

Formula Calculator Element Limits

| Element | Min | Max |
|---|---|---|
| C | 0 | 200 |
| H | 0 | 400 |
| O | 1 | 15 |
| Na | 1 | 1 |
| N | 1 | 1 |

Formula Calculator Results

| Formula | Calculated Mass | Mz | Diff. (mDa) | Diff. (ppm) | DBE |
|---|---|---|---|---|---|
| C26 H37 N Na O13 | 594.2163 | 594.2160 | 0.3 | 0.4 | 8.5 |

End Of Report

**Fig. S27** HRESIMS spectrum of compound **3**

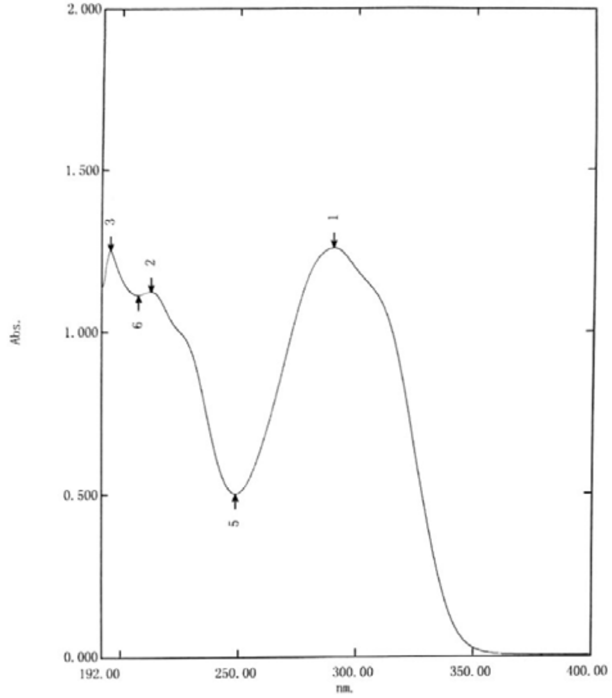


[测定属性]
波长范围(nm): 192.00 到 400.00
扫描速度: 中速
采样间隔: 0.2
自动采样间隔: 启用
扫描模式: 单个

[仪器属性]
仪器类型: UV-2700 系列
测定方式: 吸收值
狭缝宽: 5.0 nm
积分时间: 0.1 秒
光源转换波长: 323.0 nm
检测器单元: 直接
S/R 转换: 标准
阶梯校正: OFF

[附件属性]
附件: 无

[数据处理参数]
阈值: 0.0010000
点: 4
内插: 停用
平均: 停用

[样品准备属性]
重量: 1.5mg
体积: 1.5ml
稀释: 30倍
光程长: 10 mm
附加信息: 20181207 09:01:14
ped86 CH3OH

| No. | P/V | 波长(nm) | Abs. | 描述 |
|---|---|---|---|---|
| 1 | ↑ | 289.80 | 1.260 | |
| 2 | ↑ | 212.80 | 1.124 | |
| 3 | ↑ | 195.60 | 1.250 | |
| 4 | ↓ | 379.60 | 0.002 | |
| 5 | ↓ | 248.40 | 0.501 | |
| 6 | ↓ | 207.40 | 1.113 | |

**Fig. S28** UV spectrum of compound **3**

## Rudolph Research Analytical

This sample was measured on an Autopol VI, Serial #91058
Manufactured by Rudolph Research Analytical, Hackettstown, NJ, USA.

Measurement Date : Friday, 07-DEC-2018

Set Temperature : OFF

Time Delay : Disabled

Delay between Measurement : Disabled

| n | Average | Std.Dev. | % RSD | Maximum | Minimum |
|---|---|---|---|---|---|
| 5 | -120.16 | 1.42 | -1.18 | -119.20 | -122.60 |

| S.No | Sample ID | Time | Result | Scale | OR °Arc | WLG.nm | Lg.mm | Conc.g/100ml | Temp. |
|---|---|---|---|---|---|---|---|---|---|
| 1 | ped86 | 09:00:51 AM | -122.60 | SR | -0.1226 | 589 | 100.00 | 0.100 | 19.3 |
| 2 | ped86 | 09:01:06 AM | -120.20 | SR | -0.1202 | 589 | 100.00 | 0.100 | 19.3 |
| 3 | ped86 | 09:01:14 AM | -119.60 | SR | -0.1196 | 589 | 100.00 | 0.100 | 19.2 |
| 4 | ped86 | 09:01:23 AM | -119.20 | SR | -0.1192 | 589 | 100.00 | 0.100 | 19.2 |
| 5 | ped86 | 09:01:30 AM | -119.20 | SR | -0.1192 | 589 | 100.00 | 0.100 | 19.2 |

**Fig. S29** OR spectrum of compound **3**

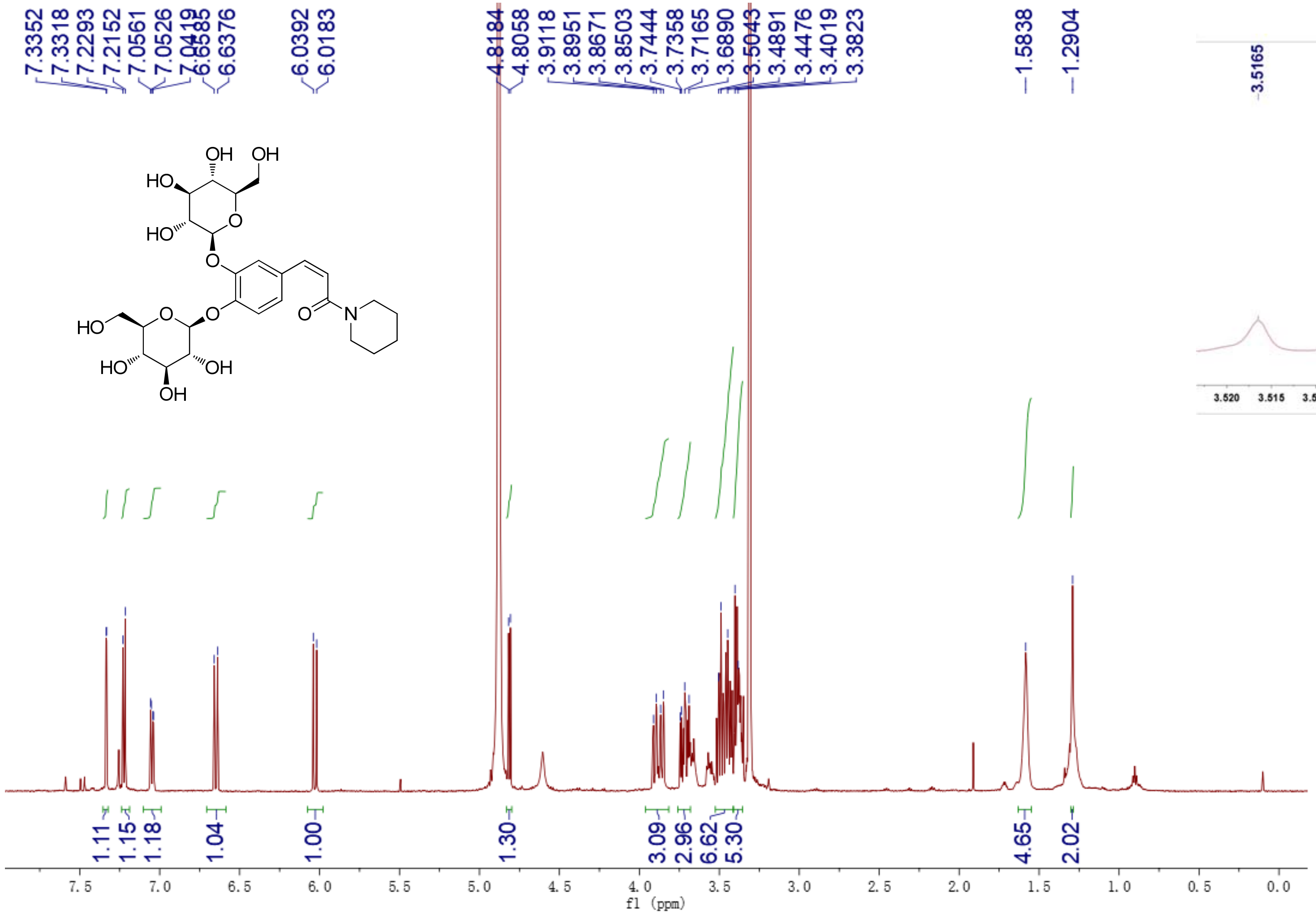

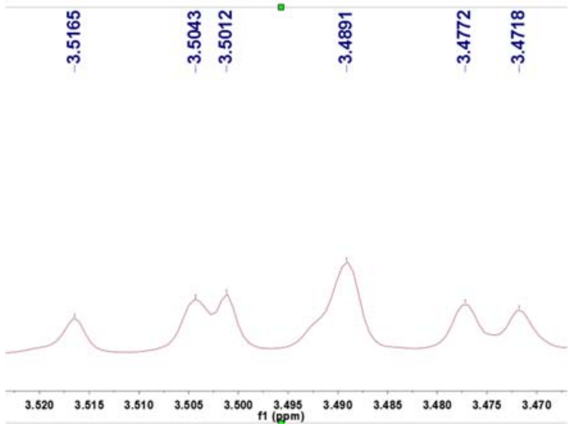


**Fig. S30** $^{1}$H NMR spectrum of compound **4** (methanol-$d_4$, 600 MHz)

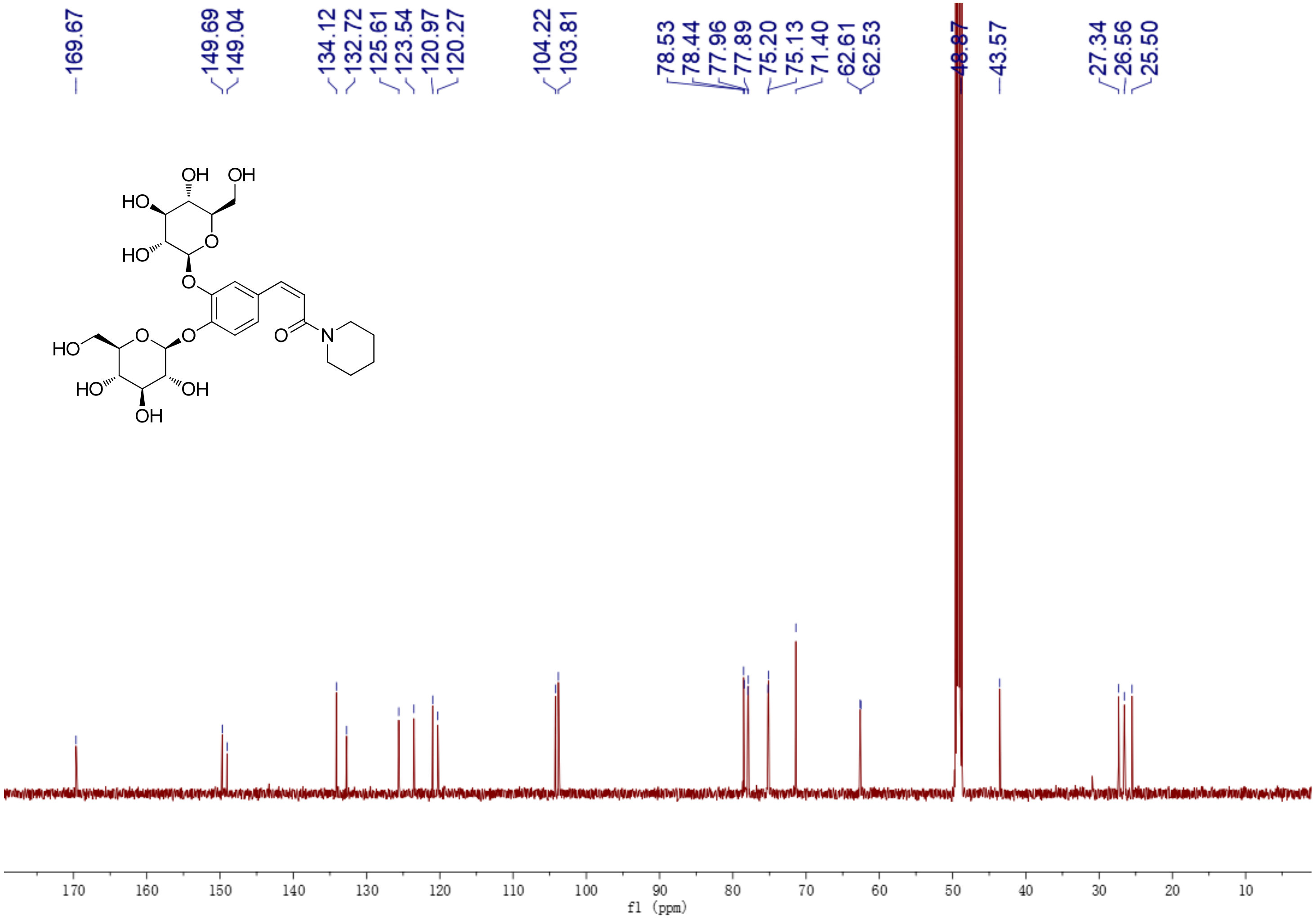


**Fig. S31** $^{13}$C NMR spectrum of compound **4** (methanol-$d_4$, 151 MHz)

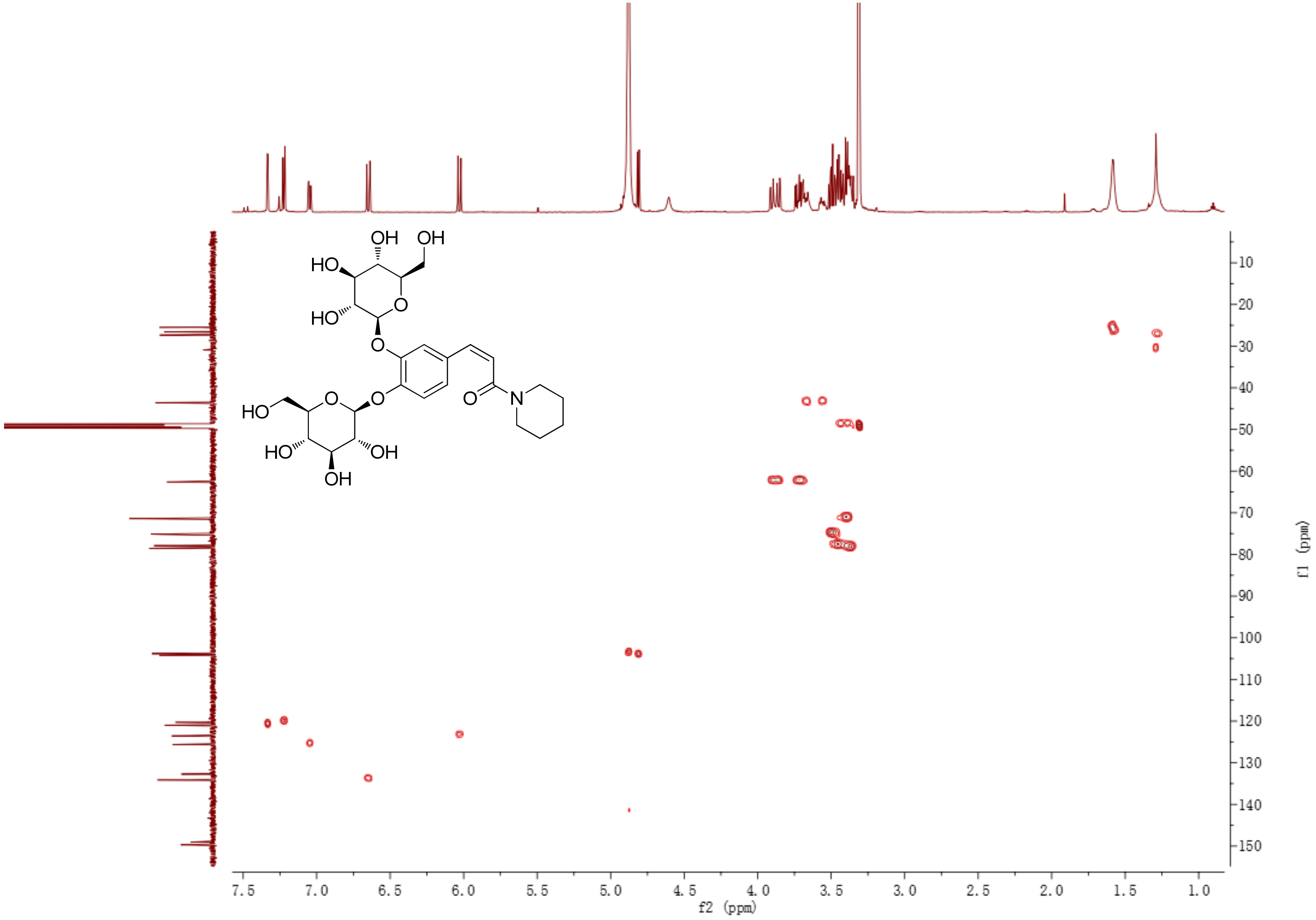


**Fig. S32** HSQC spectrum of compound **4**

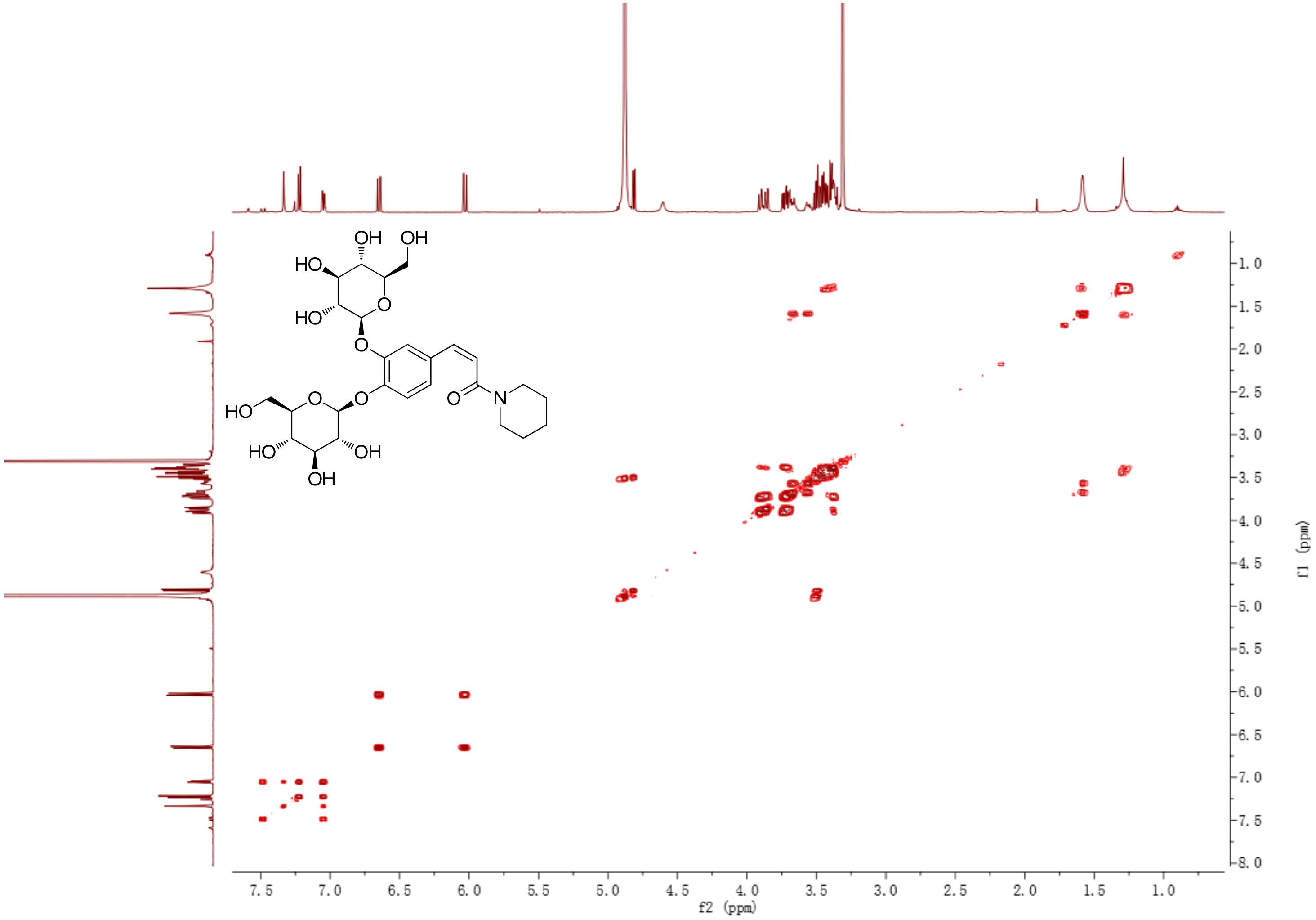


**Fig. S33** $^{1}H–^{1}H$ COSY spectrum of compound **4**

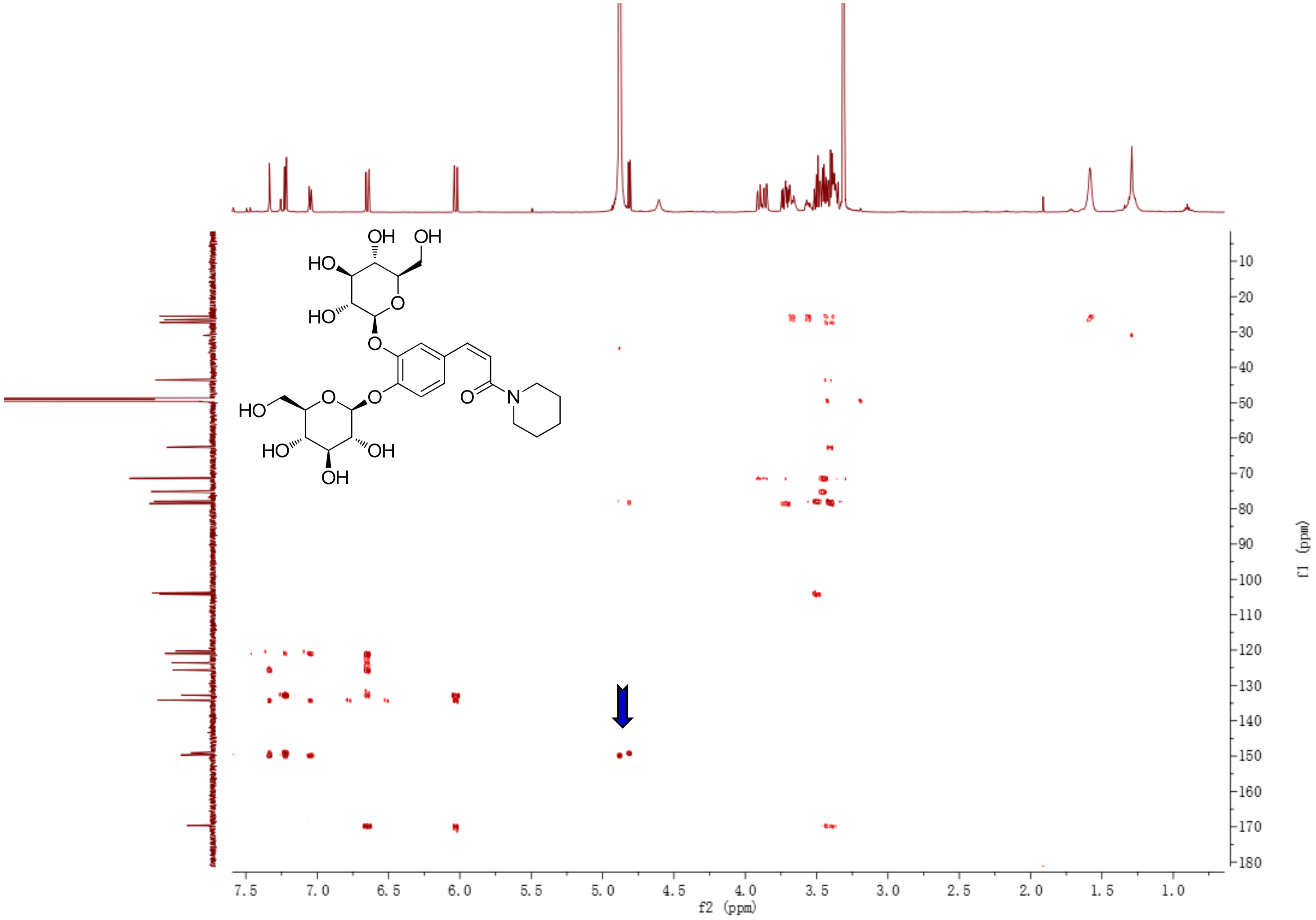


**Fig. S34** HMBC spectrum of compound **4**

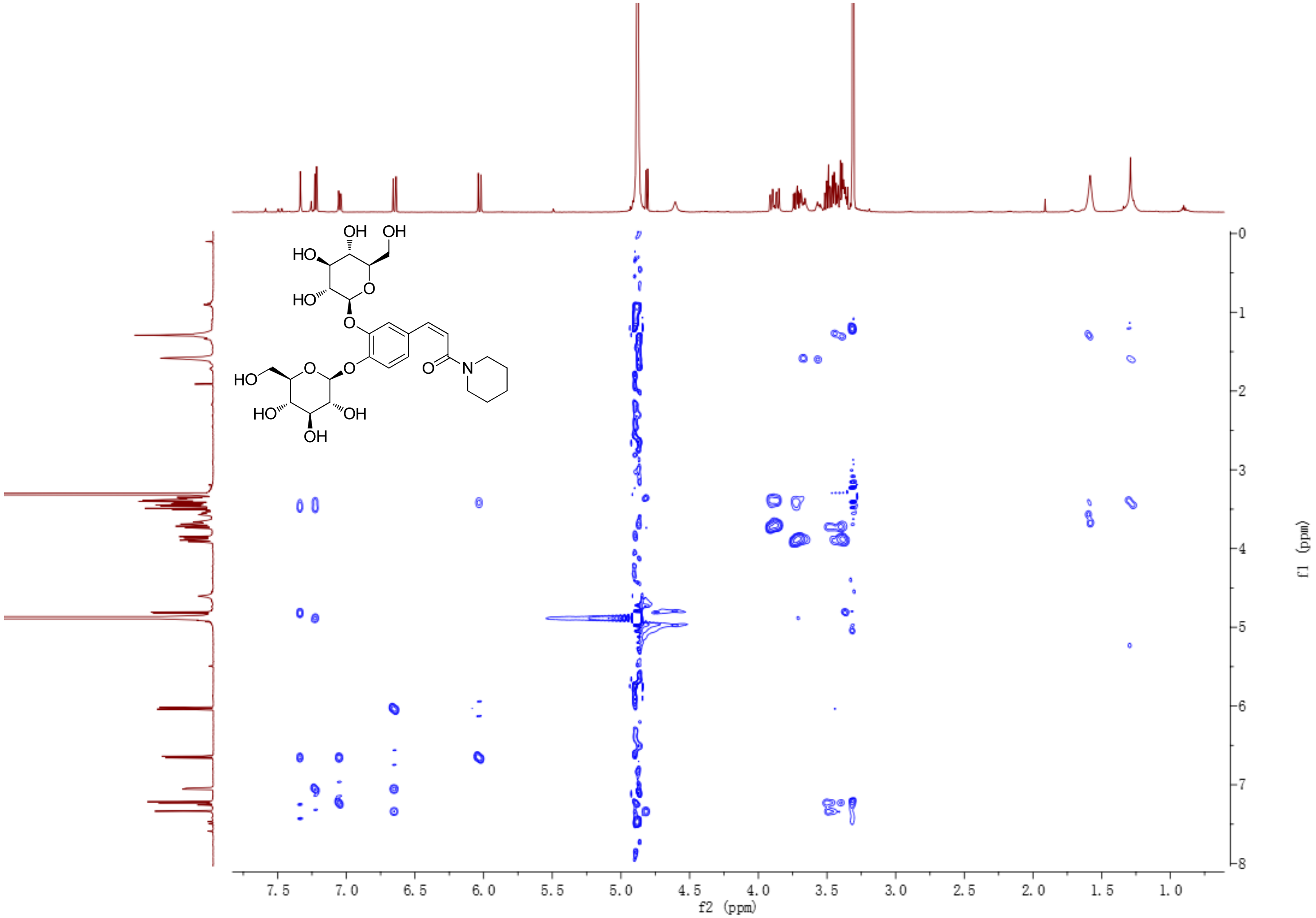


**Fig. S35** ROESY spectrum of compound **4**

## User Spectra

| Fragmentor Voltage | Collision Energy | Ionization Mode |
| --- | --- | --- |
| 200 | 0 | ESI |

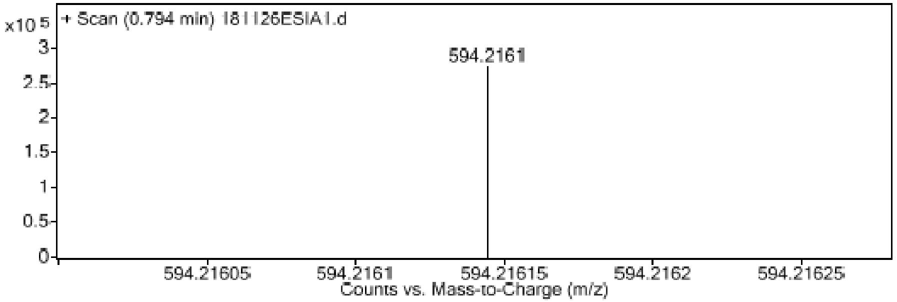


**Peak List**

| *m/z* | z | Abund | Formula | Ion |
| --- | --- | --- | --- | --- |
| 105.0426 | | 60627.76 | | |
| 118.1227 | | 18641.54 | | |
| 121.0509 | 1 | 215322.78 | | |
| 162.0771 | | 18998.56 | | |
| 284.3315 | | 20499.85 | | |
| 594.2161 | 1 | 272663.94 | C26 H37 N Na O13 | M+ |
| 595.2191 | 1 | 74589.77 | C26 H37 N Na O13 | M+ |
| 610.1895 | 1 | 22194.52 | | |
| 692.2133 | 1 | 40381.48 | | |
| 922.0098 | 1 | 83020.5 | | |

**Formula Calculator Element Limits**

| Element | Min | Max |
| --- | --- | --- |
| C | 0 | 200 |
| H | 0 | 400 |
| O | 1 | 15 |
| Na | 1 | 1 |
| N | 1 | 1 |

**Formula Calculator Results**

| Formula | CalculatedMass | Mz | Diff.(mDa) | Diff. (ppm) | DBE |
| --- | --- | --- | --- | --- | --- |
| C26 H37 N Na O13 | 594.2163 | 594.2161 | 0.2 | 0.3 | 8.5 |

--- End Of Report ---

**Fig. S36** HRESIMS spectrum of compound **4**

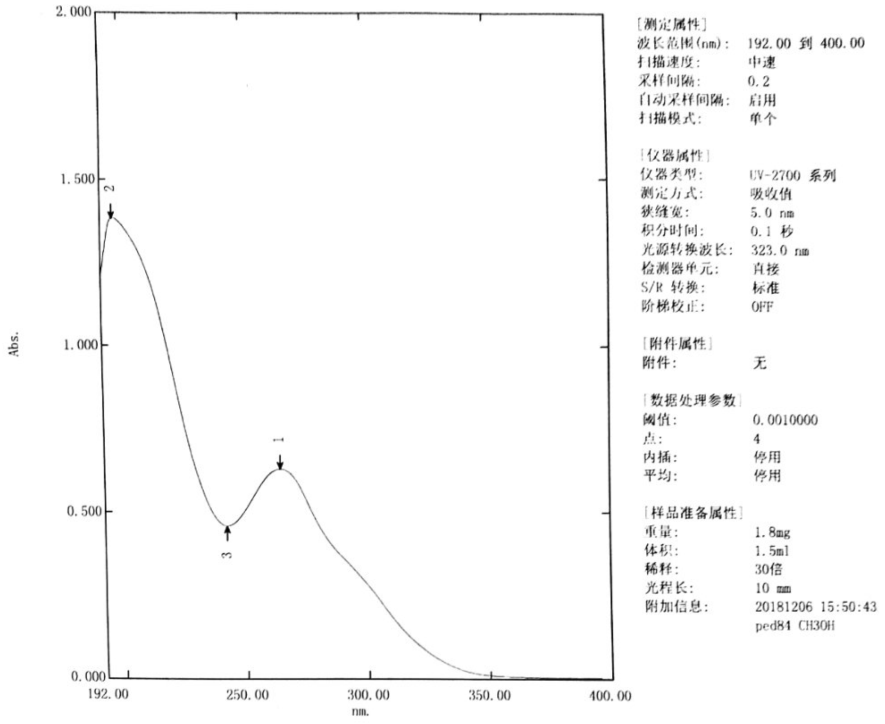


| No. | P/V | 波长(nm) | Abs. | 描述 |
|---|---|---|---|---|
| 1 | ↑ | 264.00 | 0.631 | |
| 2 | ↑ | 197.00 | 1.382 | |
| 3 | ↓ | 242.00 | 0.458 | |

**Fig. S37** UV spectrum of compound **4**

## Rudolph Research Analytical

This sample was measured on an Autopol VI, Serial #91058
Manufactured by Rudolph Research Analytical, Hackettstown, NJ, USA.

Measurement Date : Thursday, 06-DEC-2018

Set Temperature : OFF

Time Delay : Disabled

Delay between Measurement : Disabled

| n | Average | Std.Dev. | % RSD | Maximum | Minimum |
|---|---|---|---|---|---|
| 5 | -73.50 | 0.25 | -0.34 | -73.08 | -73.67 |

| S.No | Sample ID | Time | Result | Scale | OR °Arc | WLG.nm | Lg.mm | Conc.g/100ml | Temp. |
|---|---|---|---|---|---|---|---|---|---|
| 1 | ped84 | 03:42:53 PM | -73.08 | SR | -0.0877 | 589 | 100.00 | 0.120 | 21.3 |
| 2 | ped84 | 03:43:01 PM | -73.50 | SR | -0.0882 | 589 | 100.00 | 0.120 | 21.3 |
| 3 | ped84 | 03:43:09 PM | -73.67 | SR | -0.0884 | 589 | 100.00 | 0.120 | 21.3 |
| 4 | ped84 | 03:43:17 PM | -73.58 | SR | -0.0883 | 589 | 100.00 | 0.120 | 21.3 |
| 5 | ped84 | 03:43:25 PM | -73.67 | SR | -0.0884 | 589 | 100.00 | 0.120 | 21.3 |

**Fig. S38** OR spectrum of compound **4**

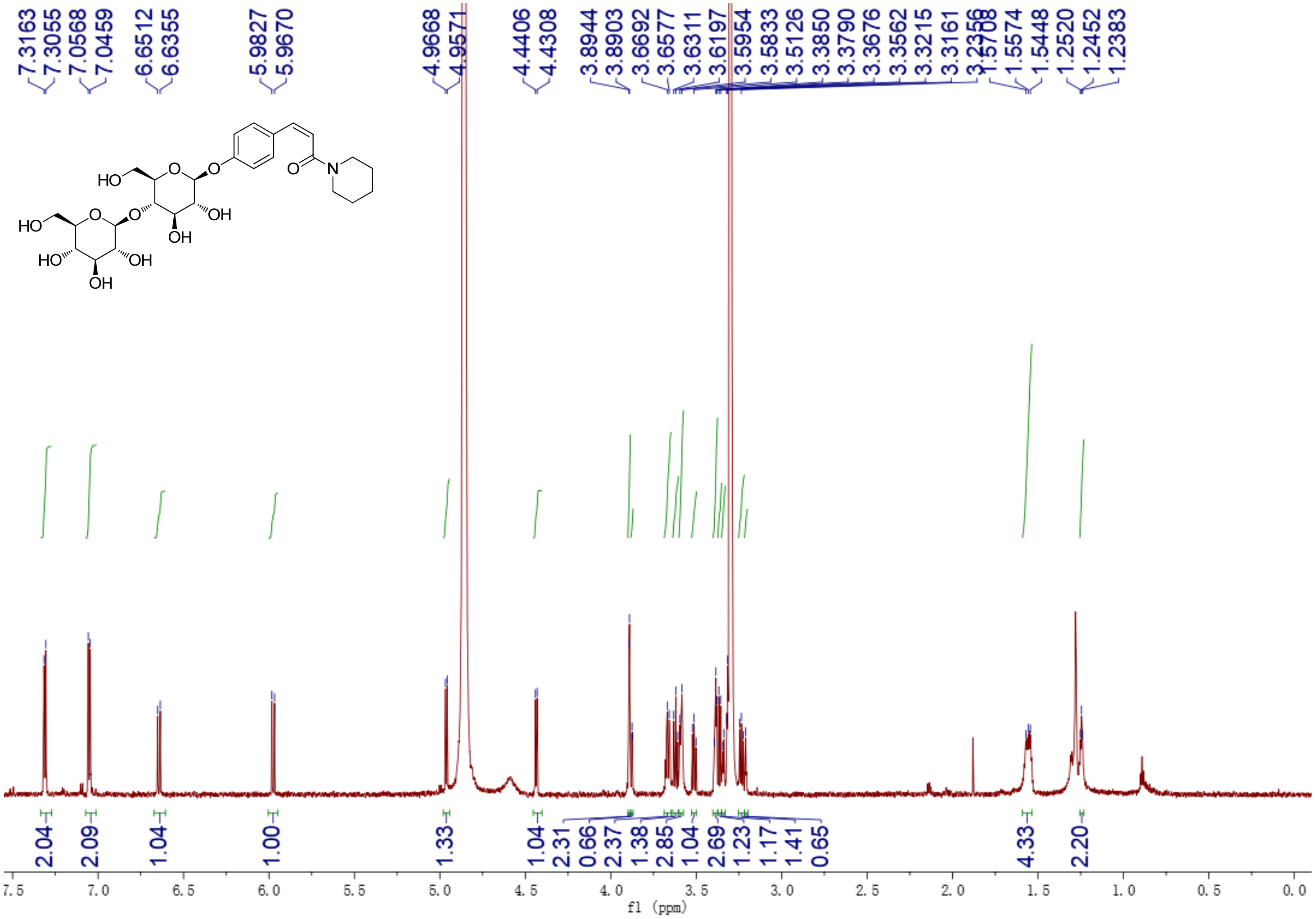


**Fig. S39** $^{1}$H NMR spectrum of compound **5** (methanol-$d_4$, 800 MHz)

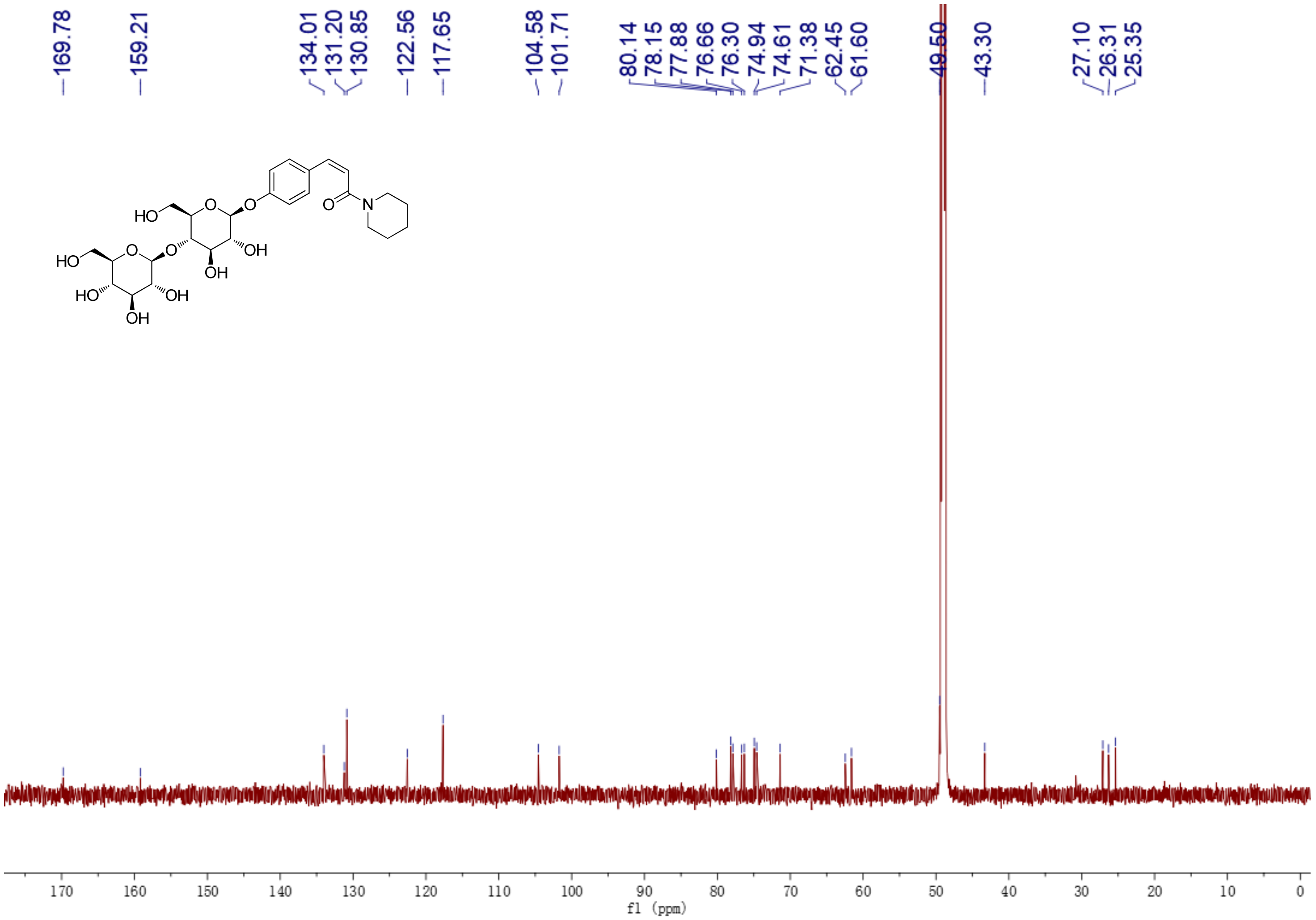


**Fig. S40** $^{13}$C NMR spectrum of compound **5** (methanol-$d_4$, 201 MHz)

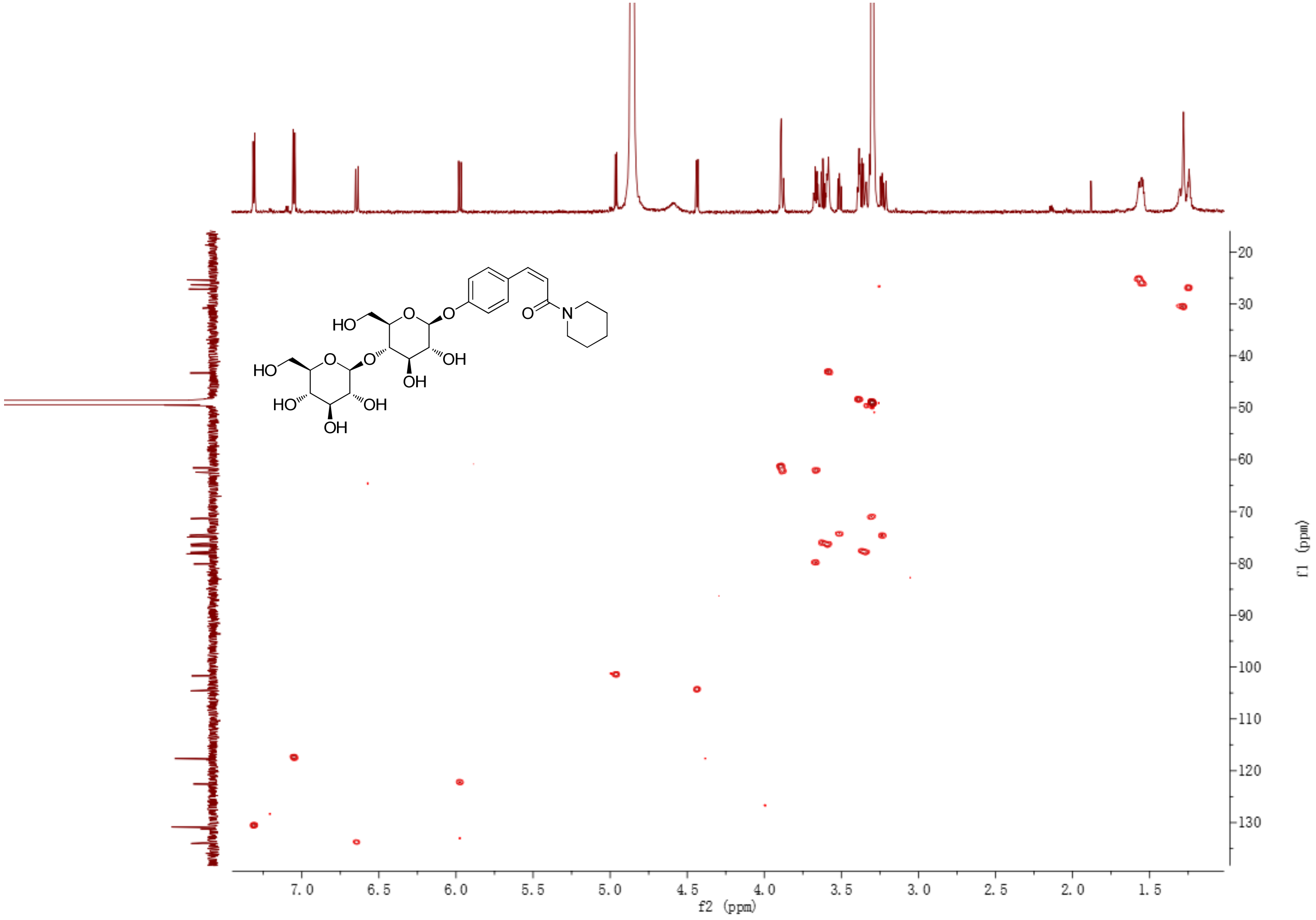


**Fig. S41** HSQC spectrum of compound **5**

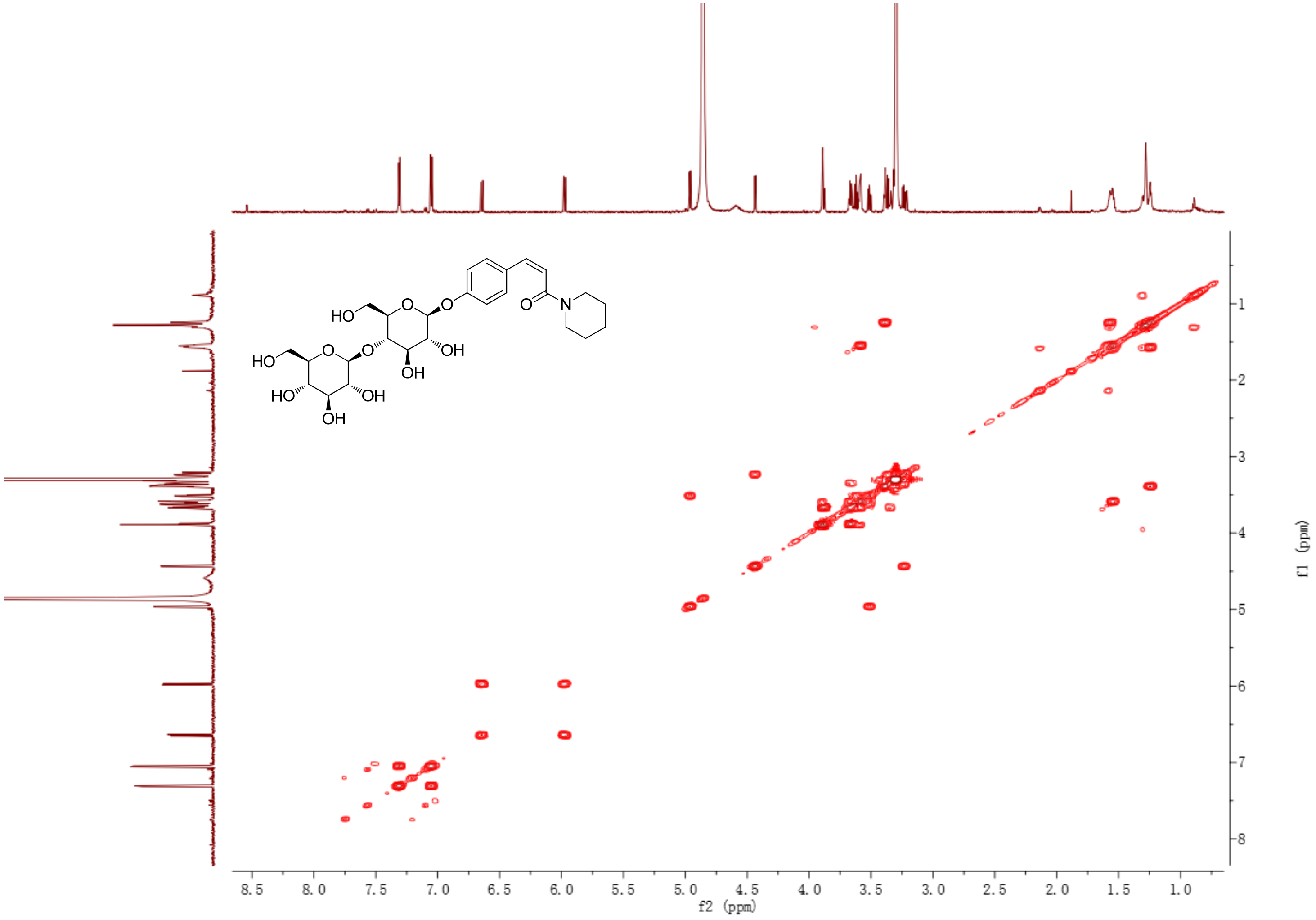


**Fig. S42** $^{1}$H–$^{1}$H COSY spectrum of compound **5**

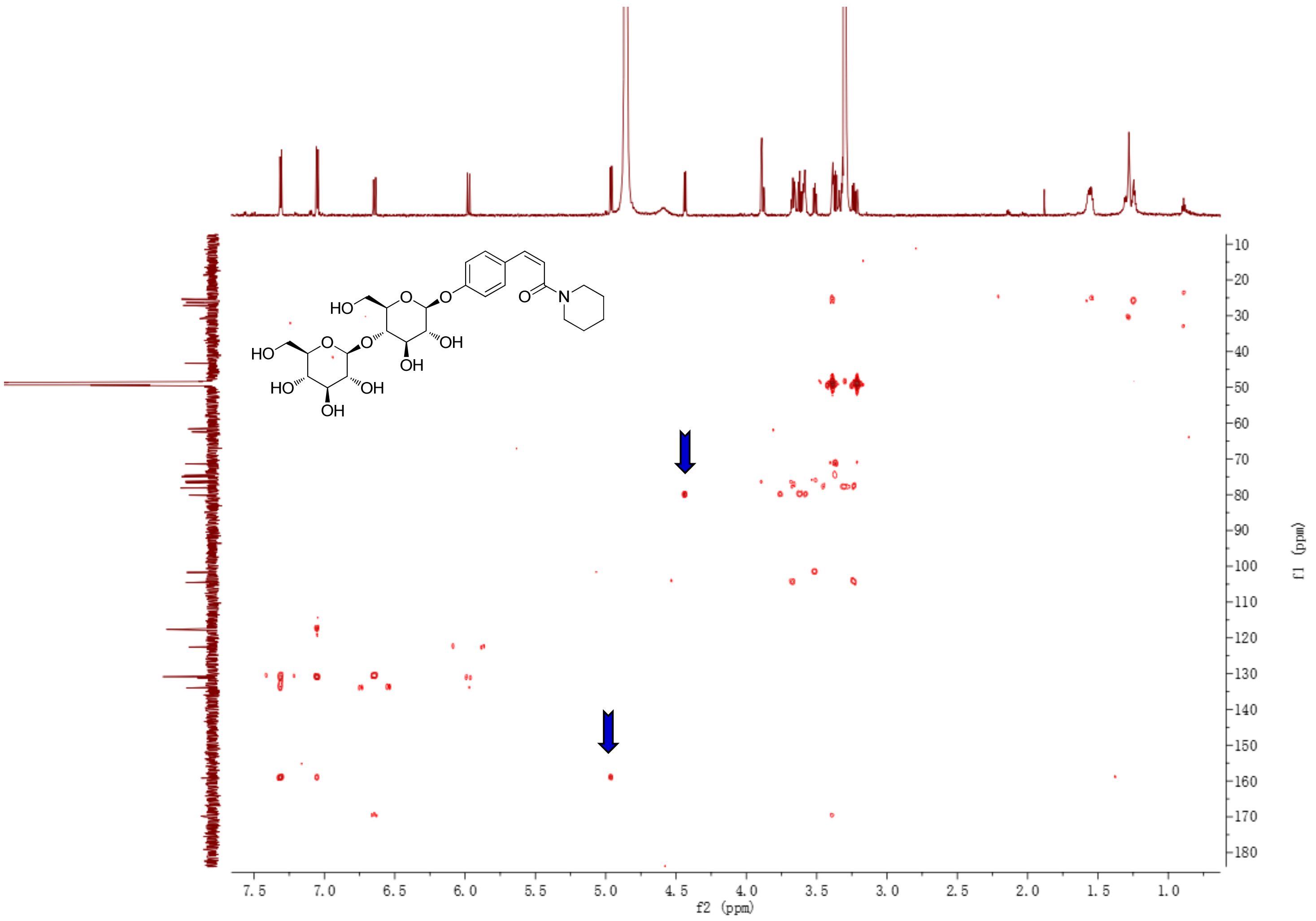


**Fig. S43** HMBC spectrum of compound **5**

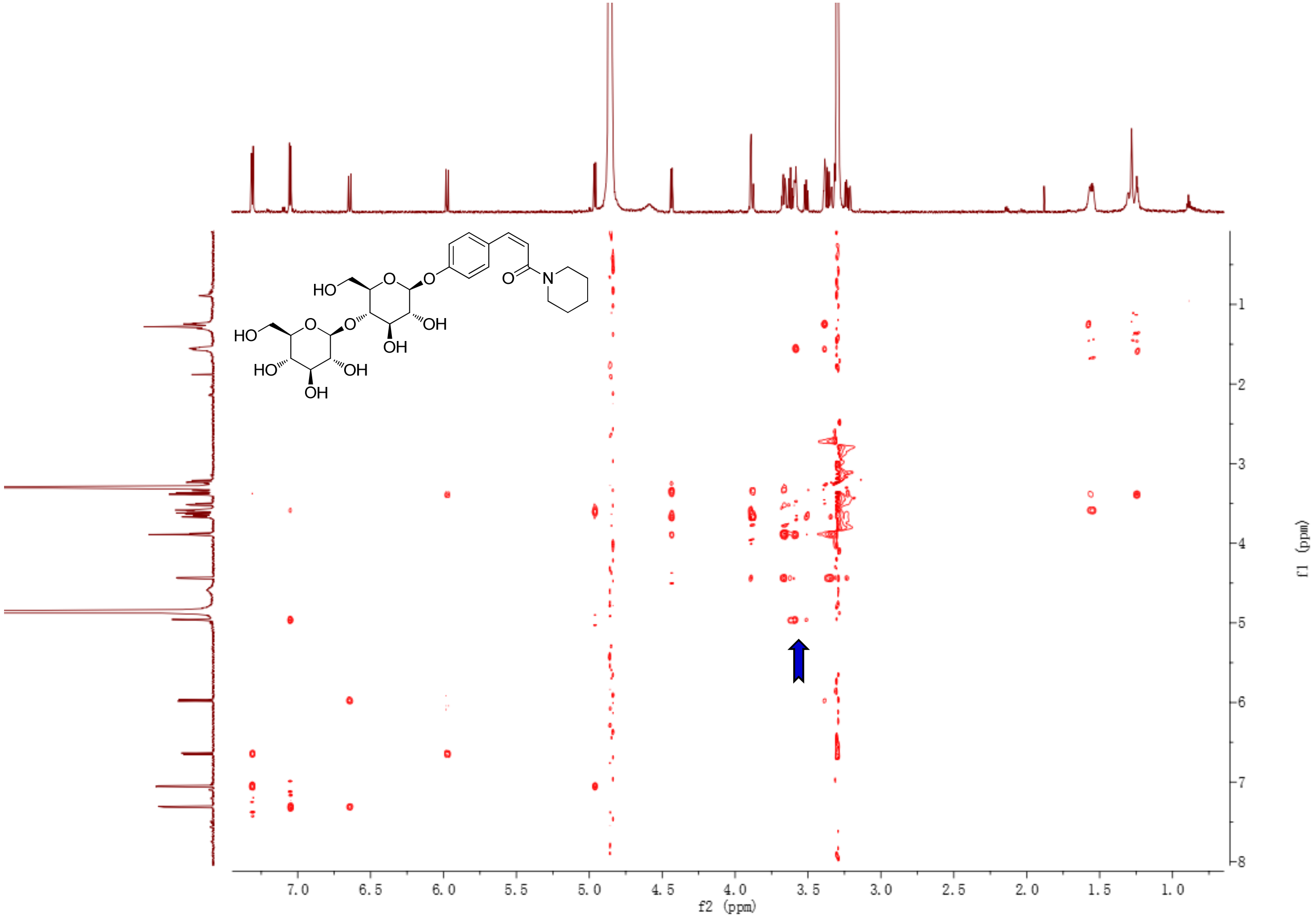


**Fig. S44** ROESY spectrum of compound **5**

## User Spectra

| Fragmentor Voltage | Collision Energy | Ionization Mode |
| --- | --- | --- |
| 200 | 0 | ESI |

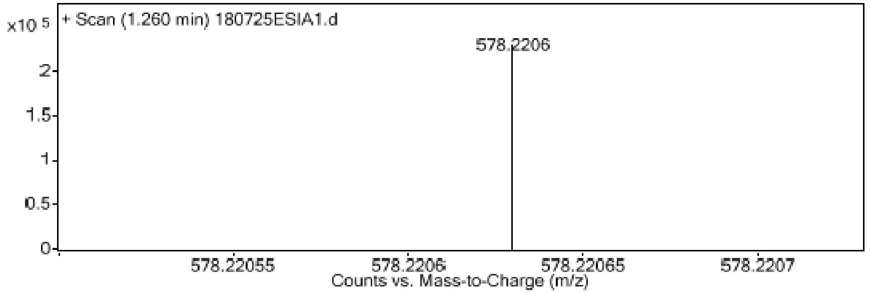


**Peak List**

| *m/z* | z | Abund | Formula | Ion |
| --- | --- | --- | --- | --- |
| 301.1411 | 1 | 30648.37 | | |
| 368.4248 | 1 | 59094.11 | | |
| 429.3189 | 1 | 30003.65 | | |
| 437.2212 | 1 | 26869.97 | | |
| 473.3444 | 1 | 29056.65 | | |
| 578.2206 | 1 | 227846.05 | C26 H37 N Na O12 | M+ |
| 579.2235 | 1 | 61222.16 | C26 H37 N Na O12 | M+ |
| 594.1944 | 1 | 28820.93 | | |
| 619.5263 | 1 | 52509.47 | | |
| 647.5577 | 1 | 37492.38 | | |

**Formula Calculator Element Limits**

| Element | Min | Max |
| --- | --- | --- |
| C | 0 | 200 |
| H | 0 | 400 |
| O | 8 | 15 |
| Na | 1 | 1 |
| N | 1 | 1 |

**Formula Calculator Results**

| Formula | CalculatedMass | Mz | Diff.(mDa) | Diff. (ppm) | DBE |
| --- | --- | --- | --- | --- | --- |
| C26 H37 N Na O12 | 578.2214 | 578.2206 | 0.8 | 1.3 | 8.5 |

--- End Of Report ---

**Fig. S45** HRESIMS spectrum of compound **5**

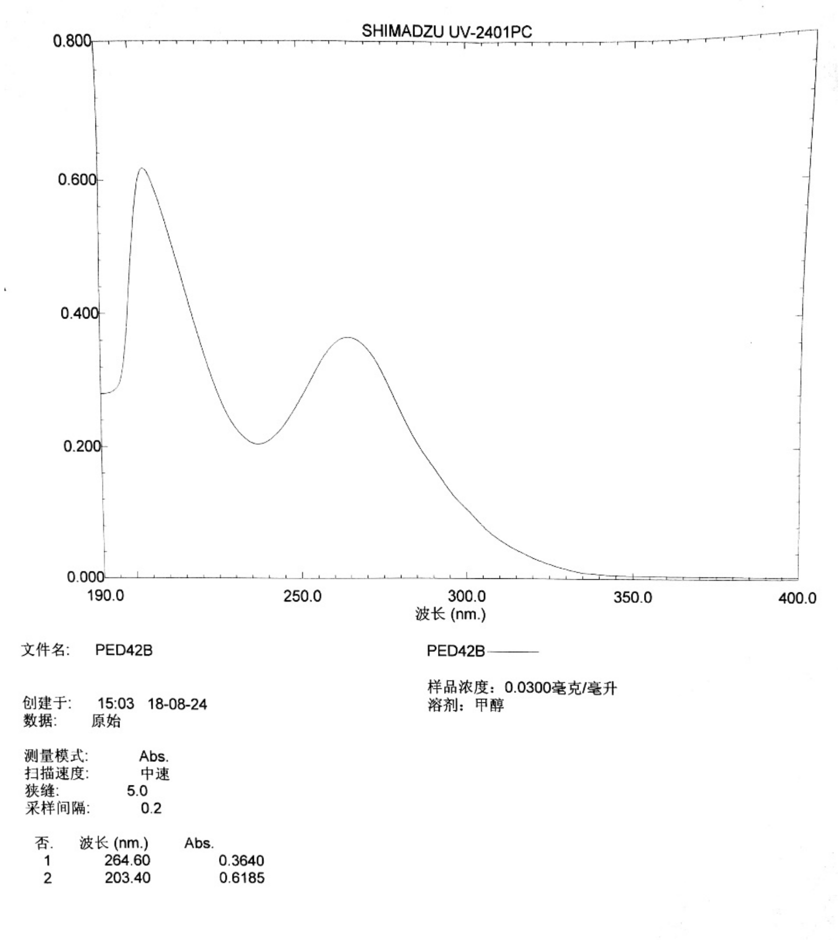


**Fig. S46** UV spectrum of compound **5**

Optical rotation measurement

Model : P-1020 (A060460638)

| No. | Sample | Mode | Data | Monitor<br>Blank | Temp.<br>Cell<br>Temp Point | Date<br>Comment<br>Sample Name | Light<br>Filter<br>Operator | Cycle Time<br>Integ Time |
|---|---|---|---|---|---|---|---|---|
| No.1 | 21 (1/3) | Sp.Rot | -45.0000 | -0.0018<br>0.0000 | 26.5<br>10.00<br>Cell | Thu Aug 23 14:21:52 2018<br>0.00040g/mL MeOH<br>PED42B | Na<br>589nm | 2 sec<br>2 sec |
| No.2 | 21 (2/3) | Sp.Rot | -45.0000 | -0.0018<br>0.0000 | 26.5<br>10.00<br>Cell | Thu Aug 23 14:21:58 2018<br>0.00040g/mL MeOH<br>PED42B | Na<br>589nm | 2 sec<br>2 sec |
| No.3 | 21 (3/3) | Sp.Rot | -47.5000 | -0.0019<br>0.0000 | 26.5<br>10.00<br>Cell | Thu Aug 23 14:22:03 2018<br>0.00040g/mL MeOH<br>PED42B | Na<br>589nm | 2 sec<br>2 sec |

**Fig. S47** OR spectrum of compound **5**

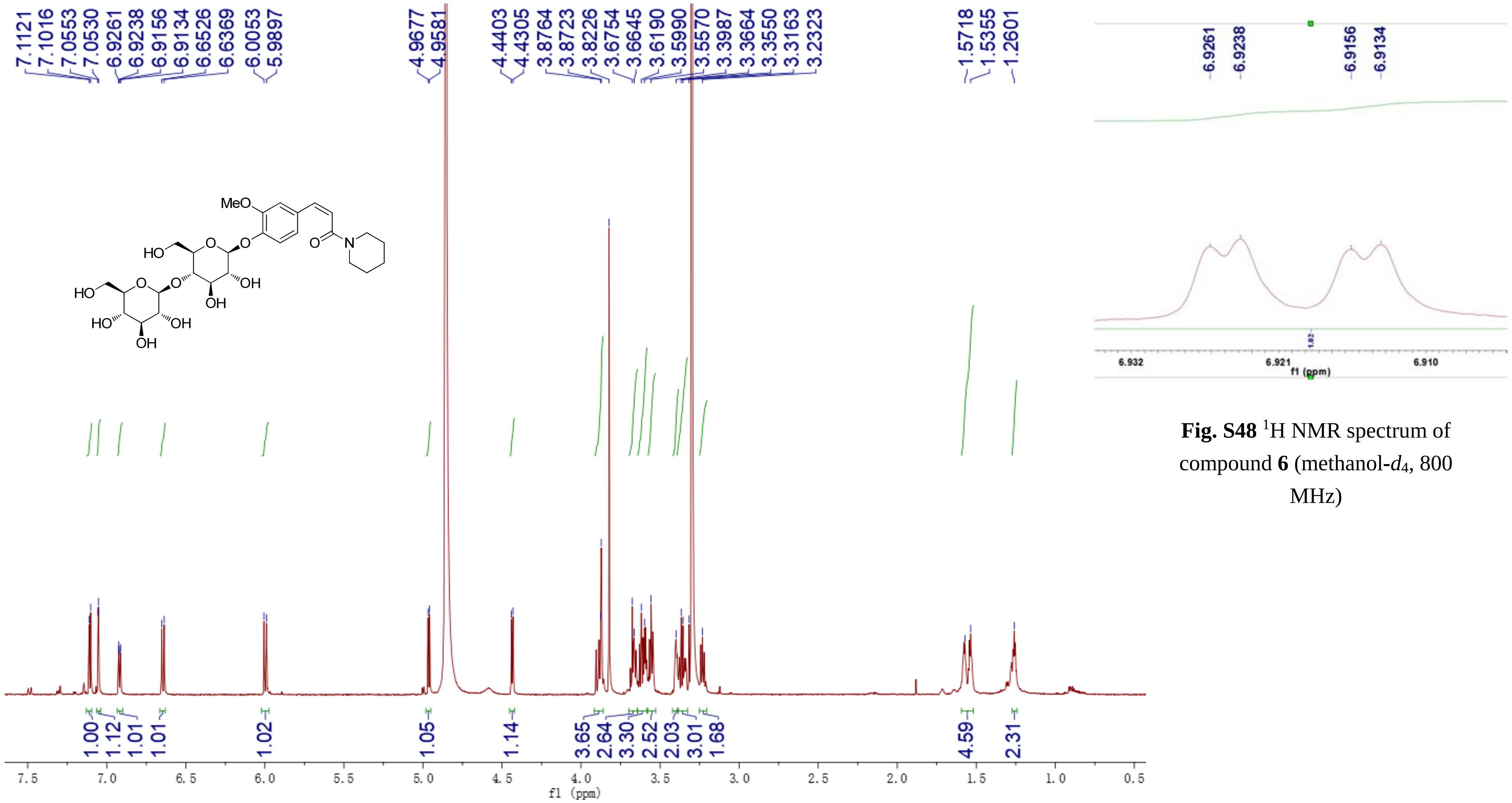


**Fig. S48** $^{1}$H NMR spectrum of compound **6** (methanol-$d_4$, 800 MHz)

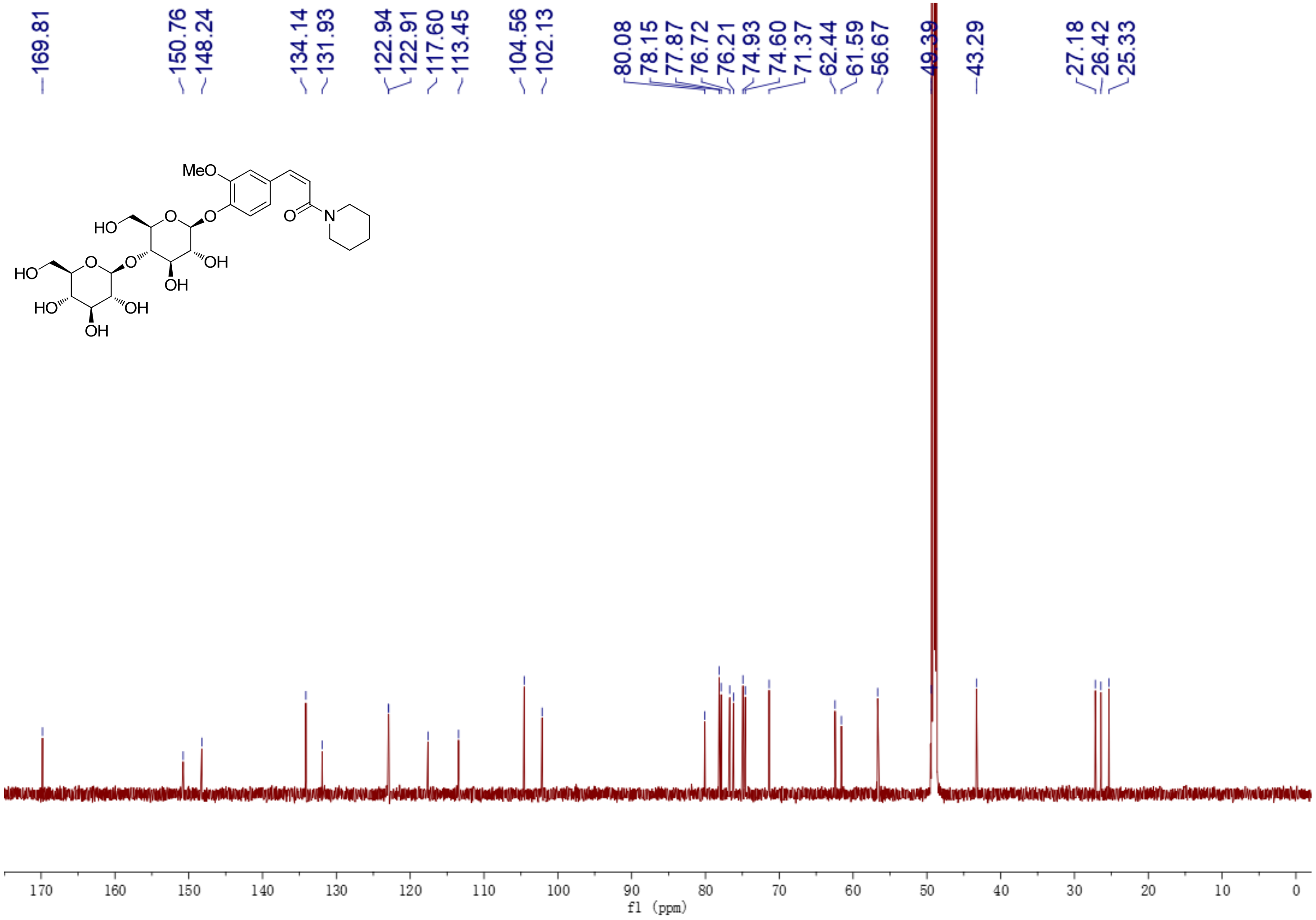


**Fig. S49** $^{13}C$ NMR spectrum of compound **6** (methanol-$d_4$, 201 MHz)

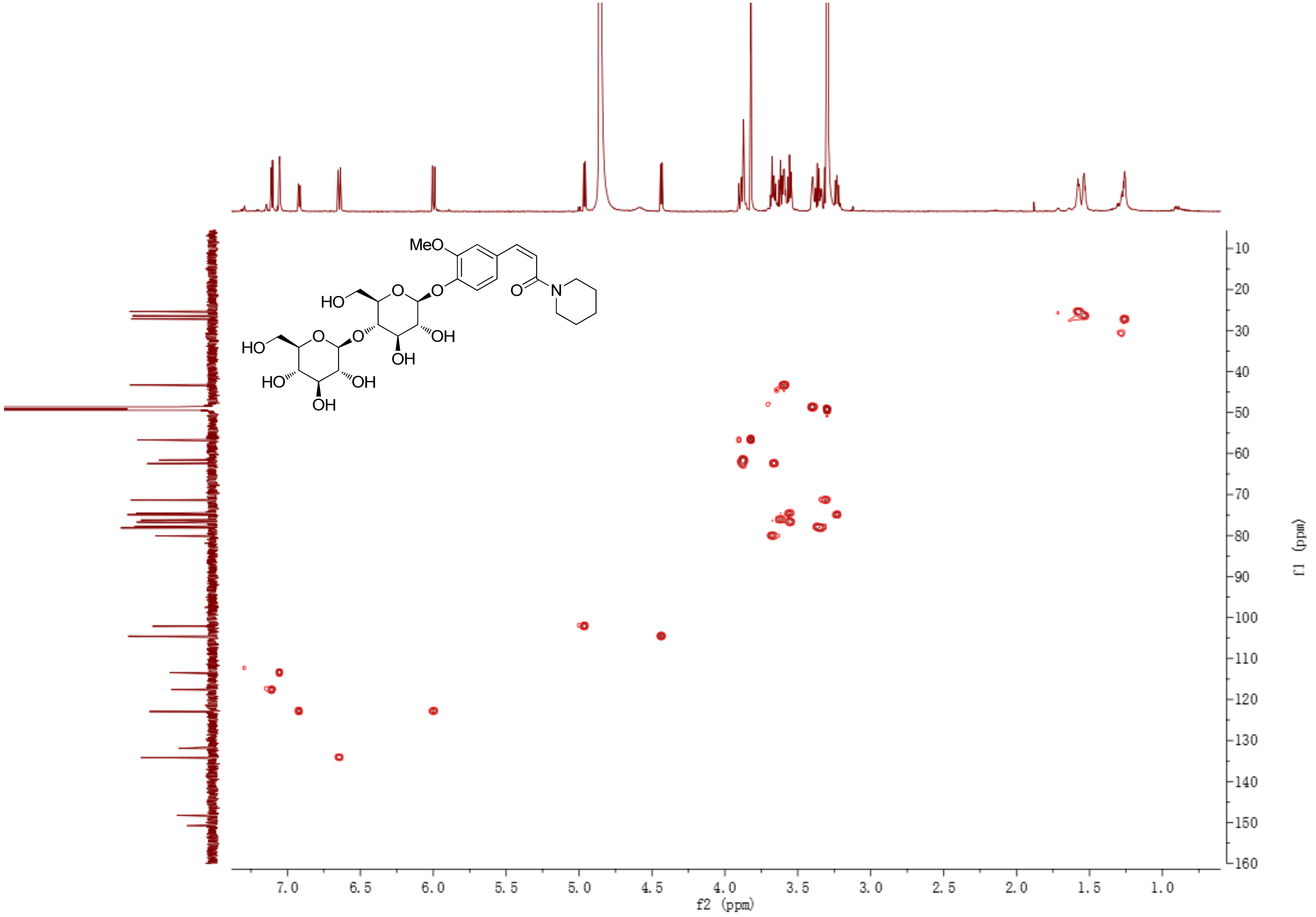


**Fig. S50** HSQC spectrum of compound **6**

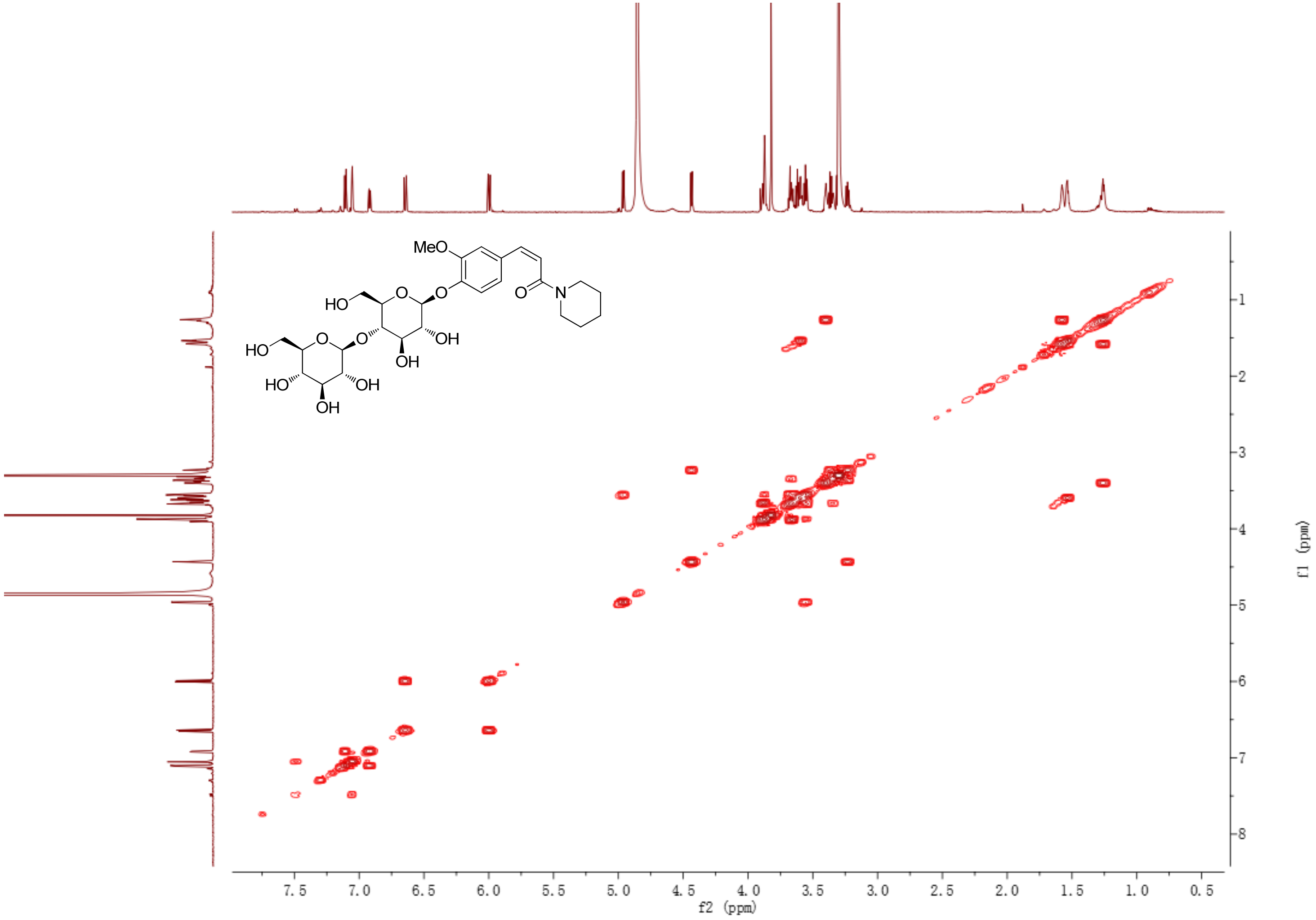


**Fig. S51** $^1H$–$^1H$ COSY spectrum of compound **6**

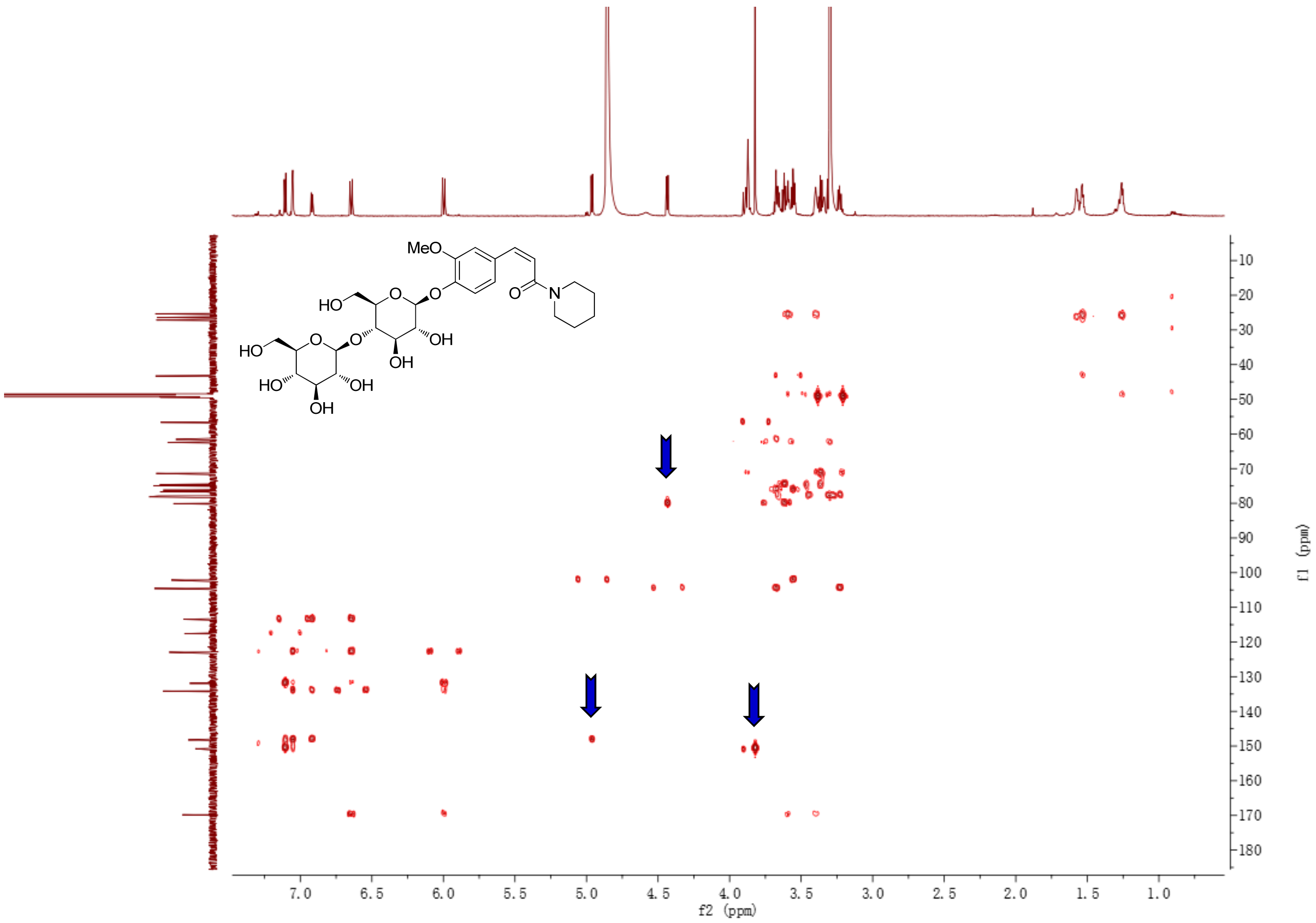


**Fig. S52** HMBC spectrum of compound **6**

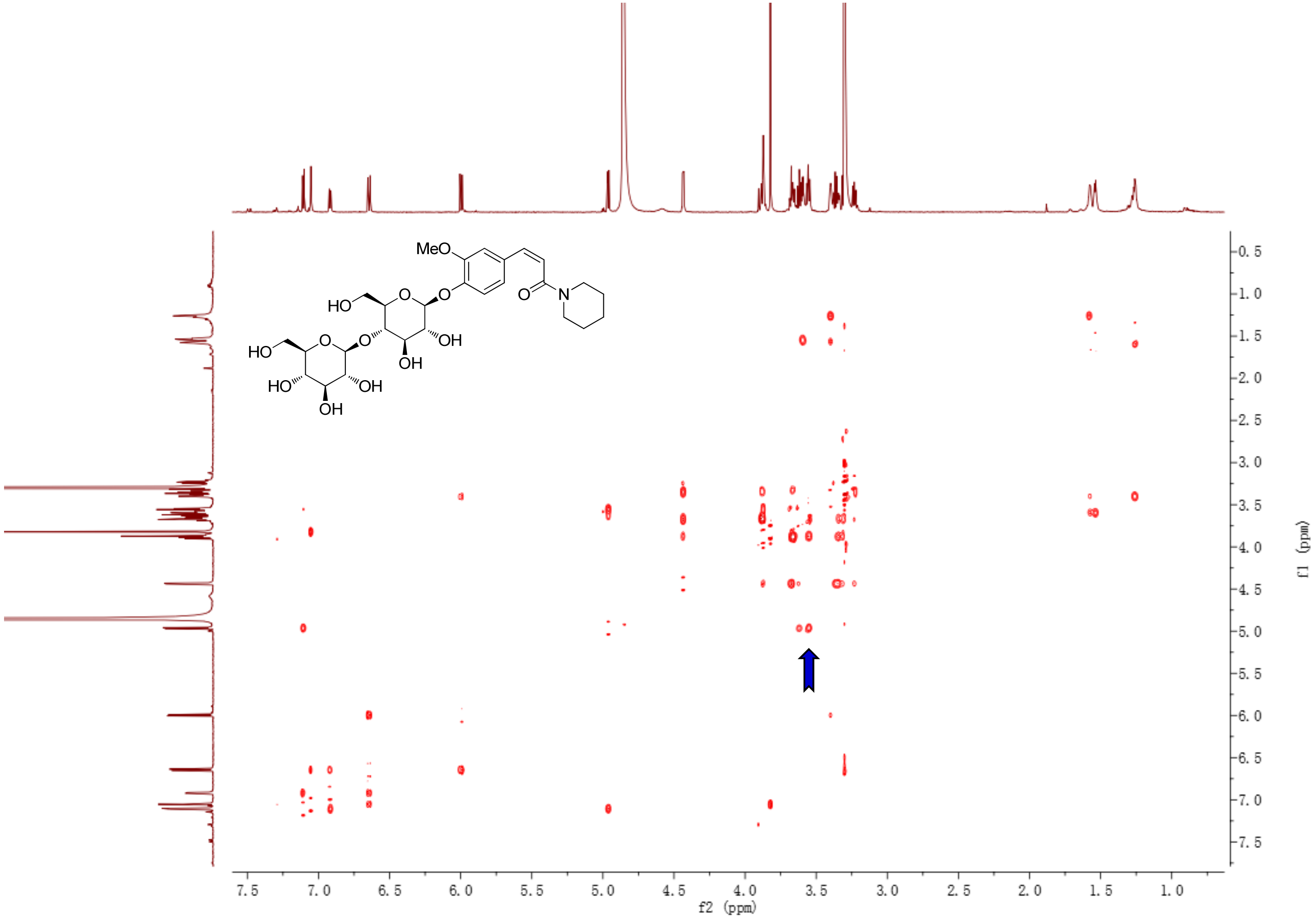


**Fig. S53** ROESY spectrum of compound **6**

## User Spectra

| Fragmentor Voltage | Collision Energy | Ionization Mode |
| --- | --- | --- |
| 200 | 0 | ESI |

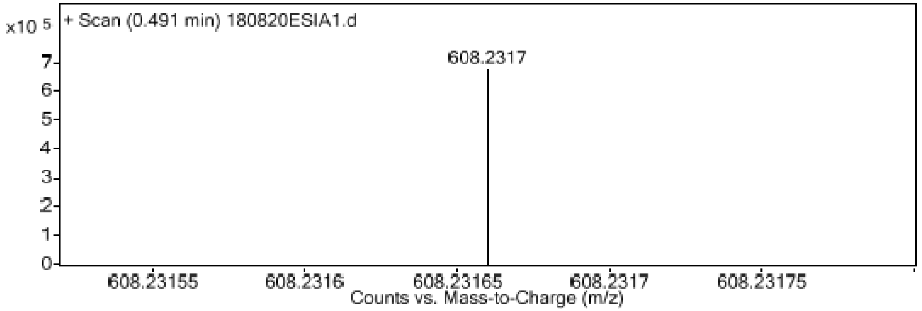


**Peak List**

| *m/z* | z | Abund | Formula | Ion |
| --- | --- | --- | --- | --- |
| 144.0805 | 1 | 56050.83 | | |
| 368.4247 | 1 | 32551.8 | | |
| 608.2317 | 1 | 674847.69 | C27 H39 N Na O13 | M+ |
| 609.2346 | 1 | 190837.41 | C27 H39 N Na O13 | M+ |
| 610.2362 | 1 | 39912.47 | C27 H39 N Na O13 | M+ |
| 619.5269 | 1 | 43459.67 | | |
| 624.2041 | 1 | 54995.59 | | |
| 630.2126 | 1 | 44484.79 | | |
| 706.2285 | 1 | 68653.93 | | |
| 1193.4715 | 1 | 34810.77 | | |

**Formula Calculator Element Limits**

| Element | Min | Max |
| --- | --- | --- |
| C | 0 | 200 |
| H | 0 | 400 |
| O | 10 | 20 |
| Na | 1 | 1 |
| N | 1 | 1 |

**Formula Calculator Results**

| Formula | CalculatedMass | Mz | Diff.(mDa) | Diff. (ppm) | DBE |
| --- | --- | --- | --- | --- | --- |
| C27 H39 N Na O13 | 608.2319 | 608.2317 | 0.2 | 0.3 | 8.5 |

--- End Of Report ---

**Fig. S54** HRESIMS spectrum of compound **6**

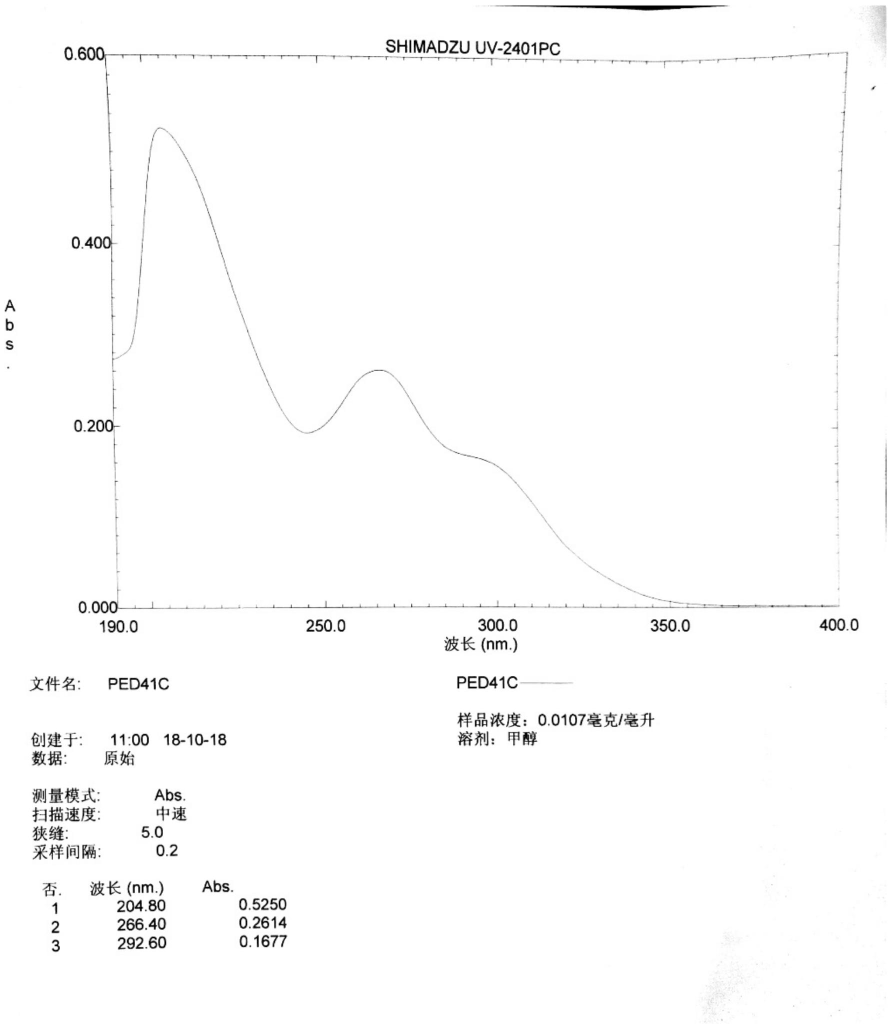


**Fig. S55** UV spectrum of compound **6**

Optical rotation measurement

Model : P-1020 (A060460638)

| No. | Sample | Mode | Data | Monitor<br>Blank | Temp.<br>Cell<br>Temp Point | Date<br>Comment<br>Sample Name | Light<br>Filter<br>Operator | Cycle Time<br>Integ Time |
|---|---|---|---|---|---|---|---|---|
| No.1 | 16 (1/3) | Sp.Rot | -56.6000 | -0.0283<br>0.0000 | 21.7<br>50.00<br>Cell | Mon Oct 15 19:35:16 2018<br>0.00100g/mL MeOH<br>PED41C | Na<br>589nm | 2 sec<br>2 sec |
| No.2 | 16 (2/3) | Sp.Rot | -58.6000 | -0.0293<br>0.0000 | 21.7<br>50.00<br>Cell | Mon Oct 15 19:35:22 2018<br>0.00100g/mL MeOH<br>PED41C | Na<br>589nm | 2 sec<br>2 sec |
| No.3 | 16 (3/3) | Sp.Rot | -58.4000 | -0.0292<br>0.0000 | 21.7<br>50.00<br>Cell | Mon Oct 15 19:35:27 2018<br>0.00100g/mL MeOH<br>PED41C | Na<br>589nm | 2 sec<br>2 sec |

**Fig. S56** OR spectrum of compound **6**

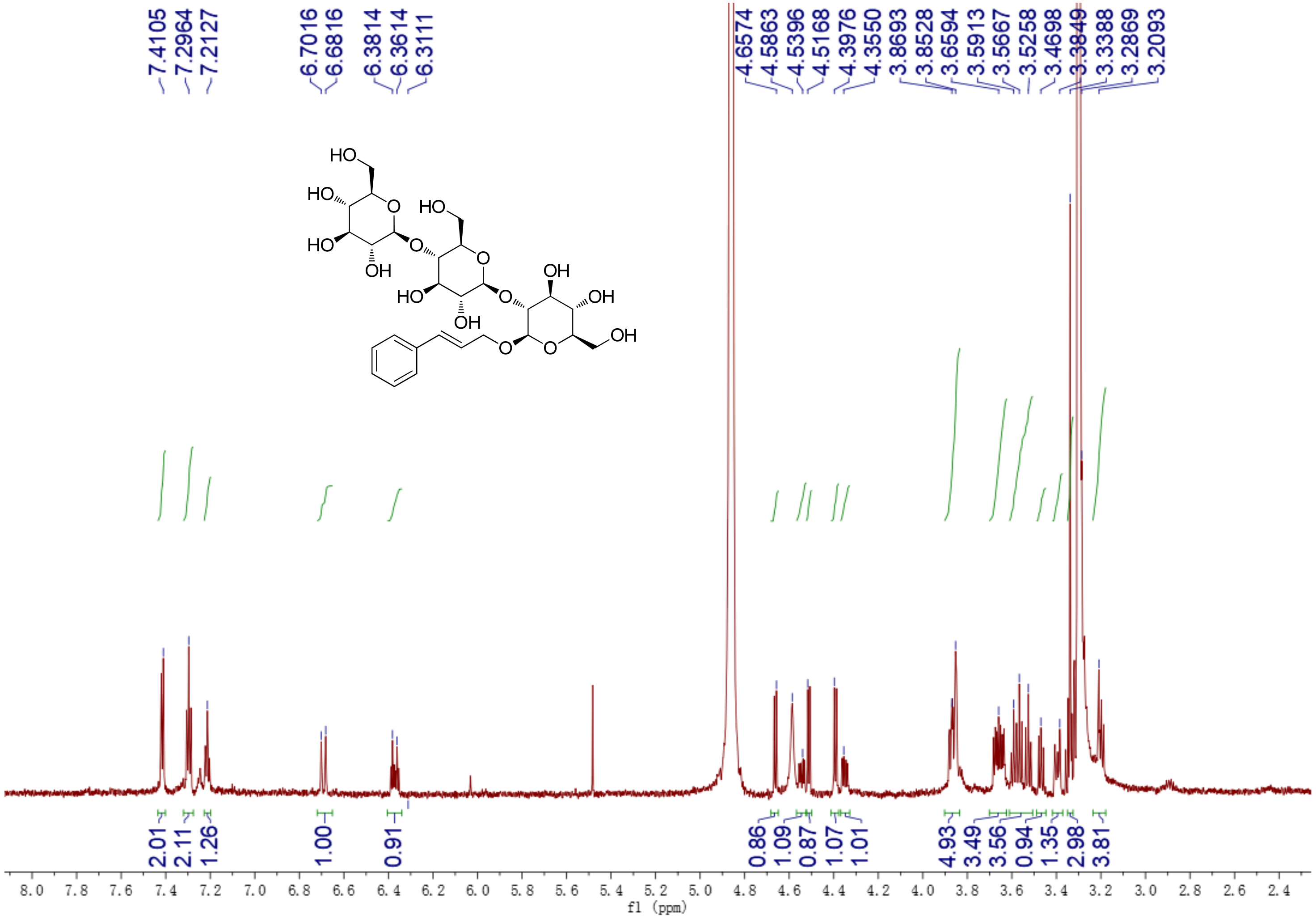


**Fig. S57** $^{1}$H NMR spectrum of compound **7** (methanol-$d_4$, 800 MHz)

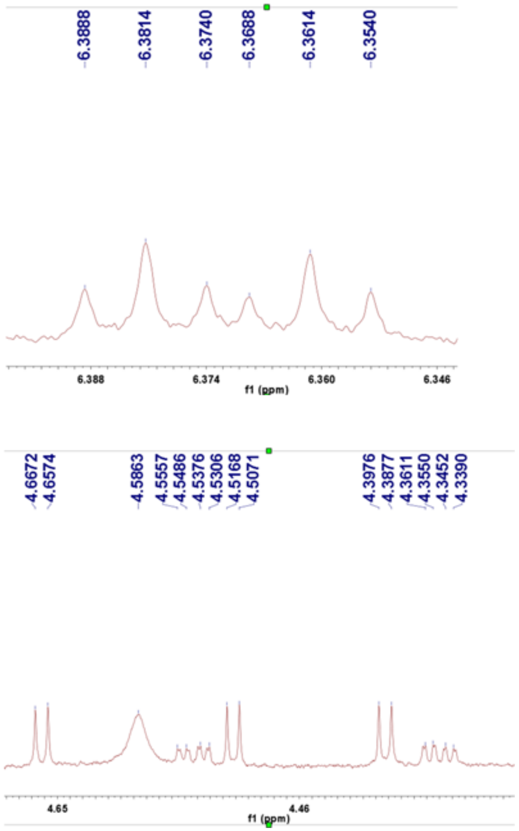

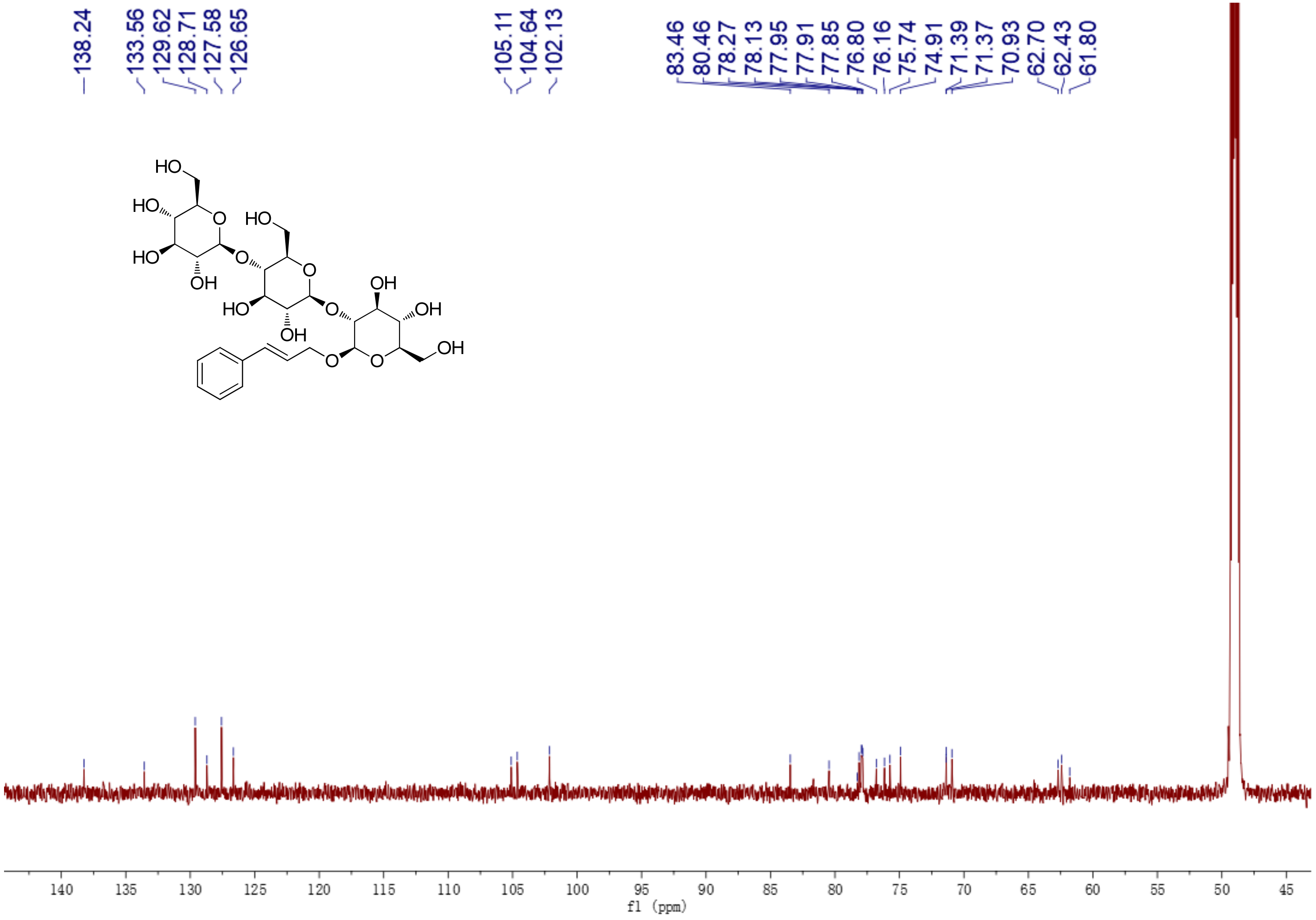


**Fig. S58** $^{13}$C NMR spectrum of compound **7** (methanol-$d_4$, 201 MHz)

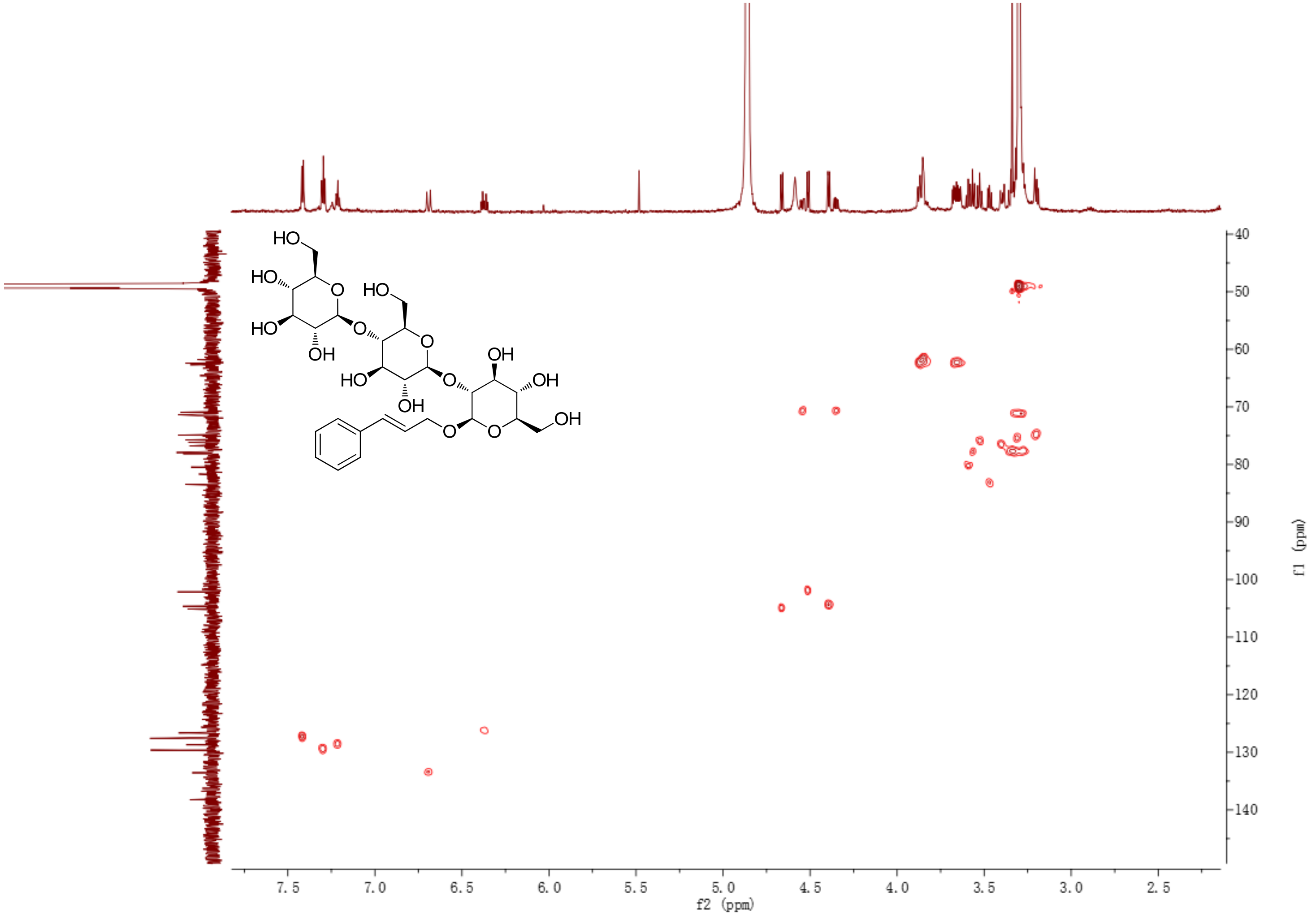


**Fig. S59** HSQC spectrum of compound **7**

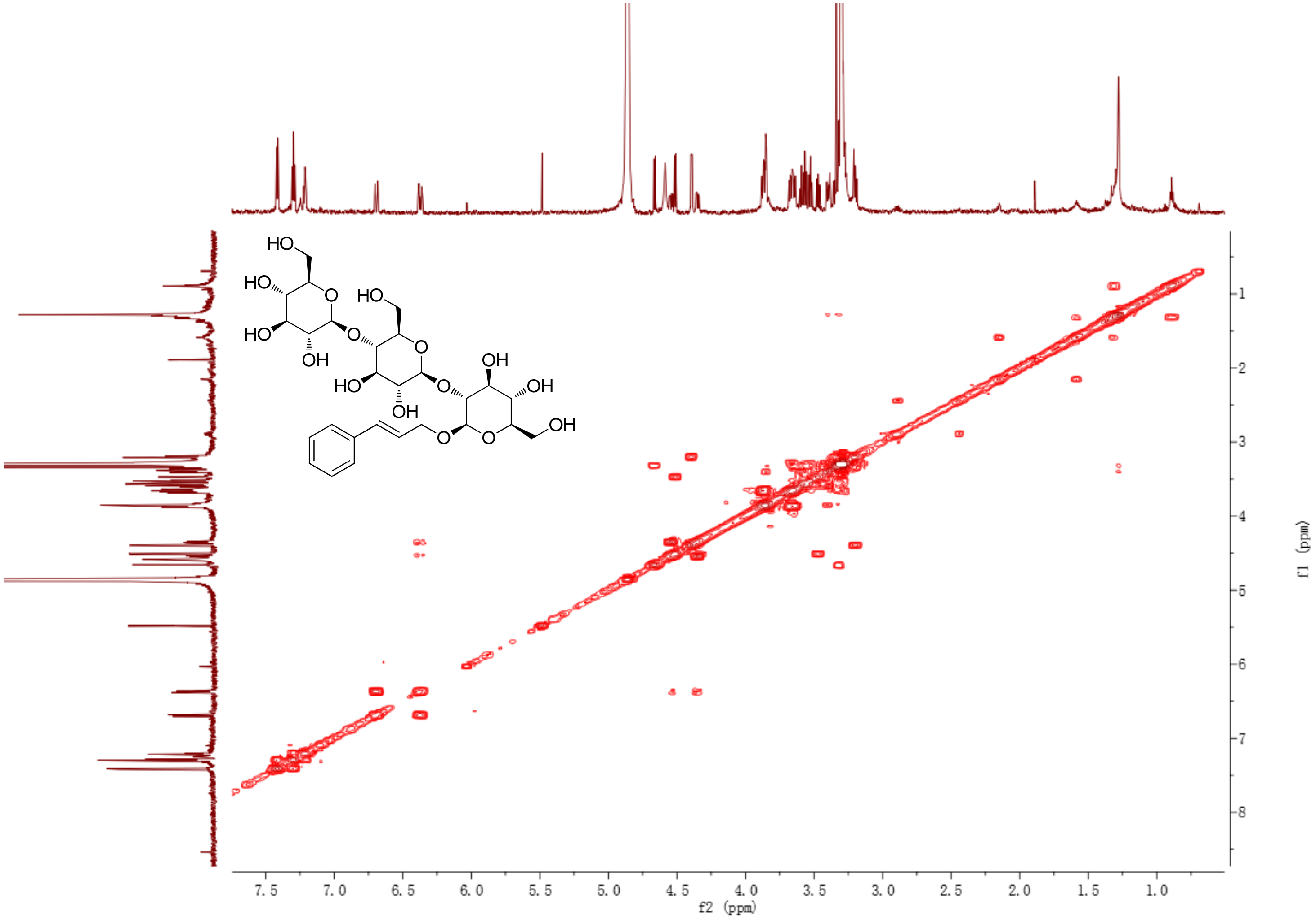


**Fig. S60** $^{1}$H–$^{1}$H COSY spectrum of compound **7**

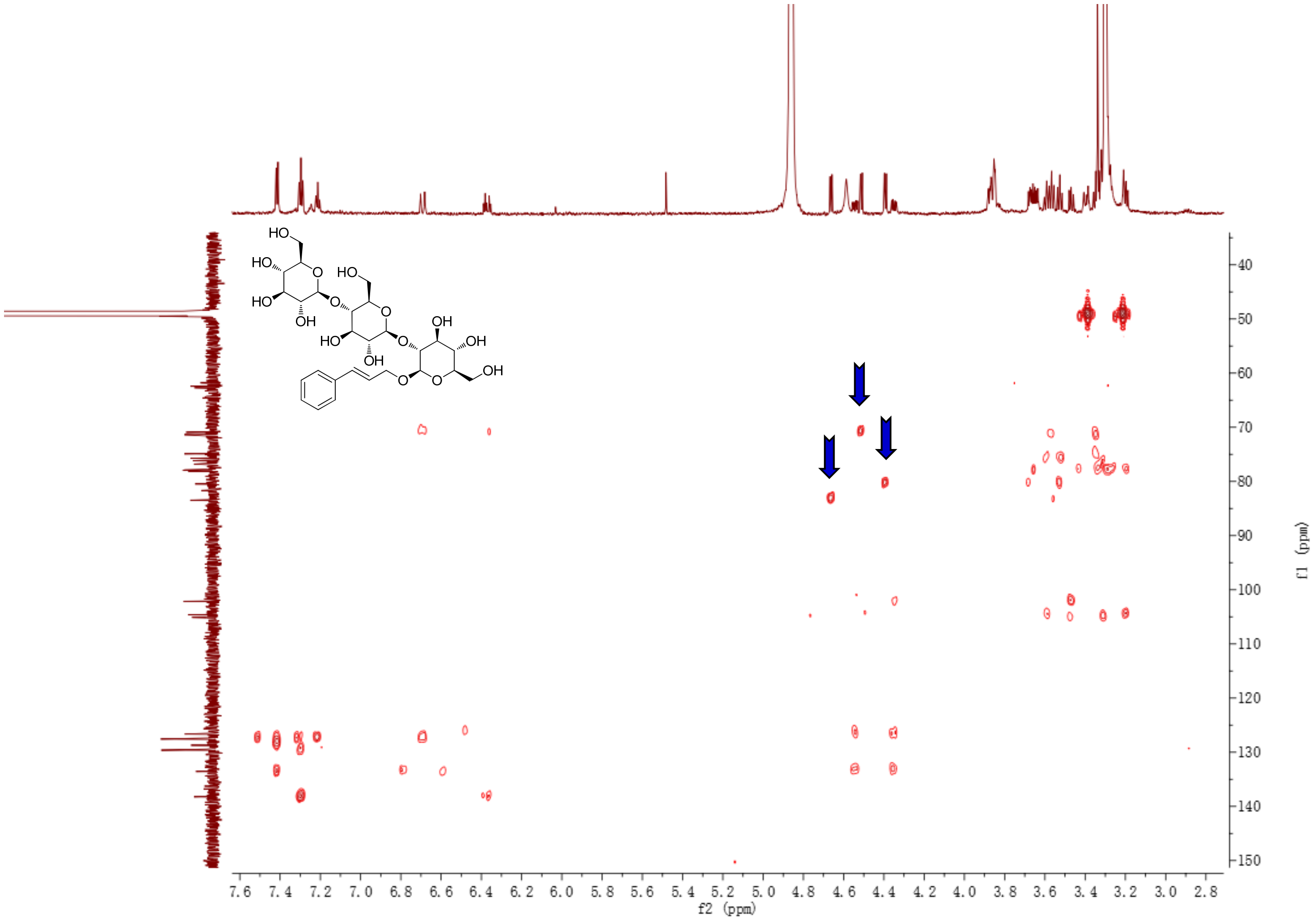


**Fig. S61** HMBC spectrum of compound **7**

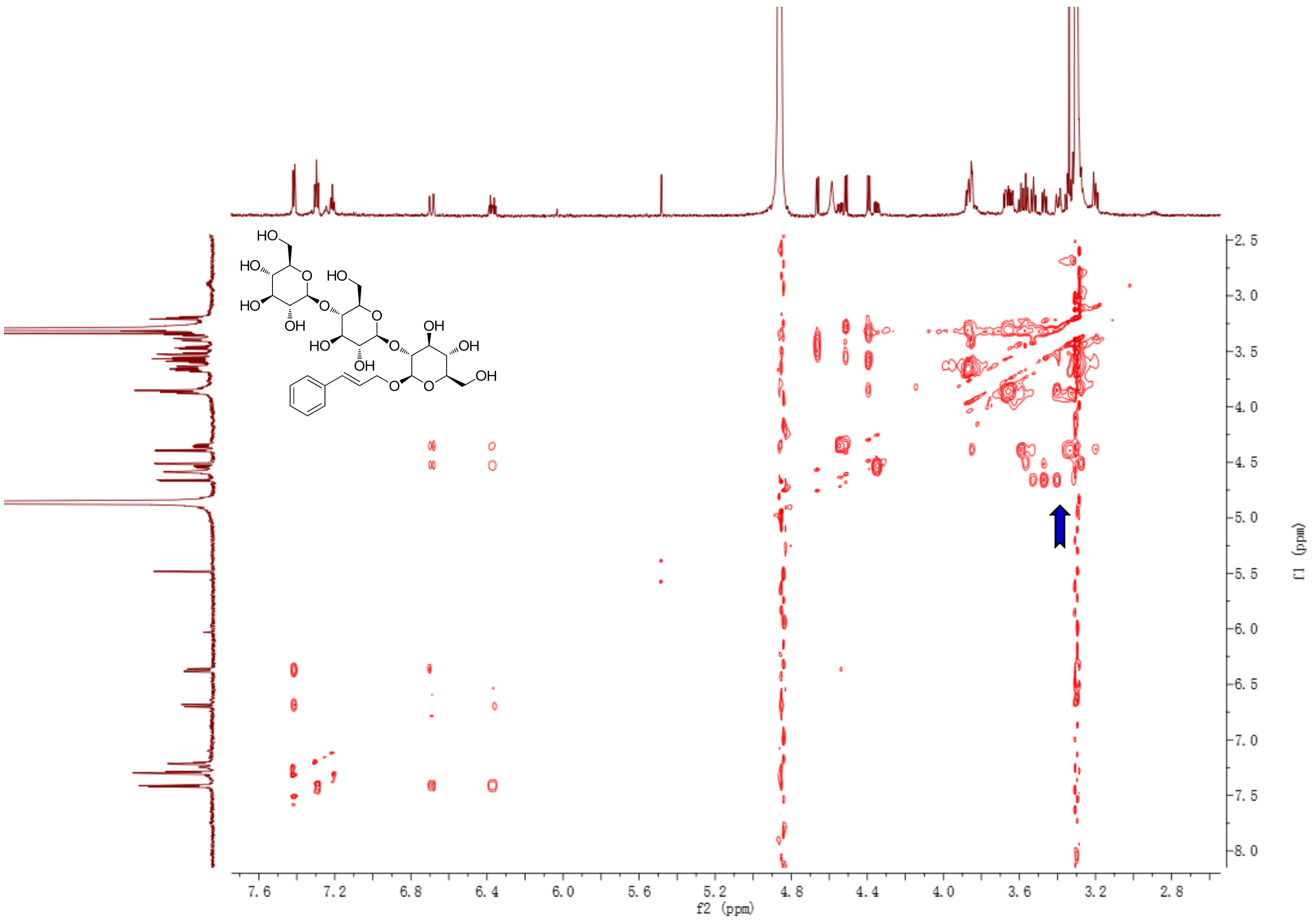


**Fig. S62** ROESY spectrum of compound **7**

## User Spectra

| Fragmentor Voltage | Collision Energy | Ionization Mode |
|---|---|---|
| 300 | 0 | ESI |

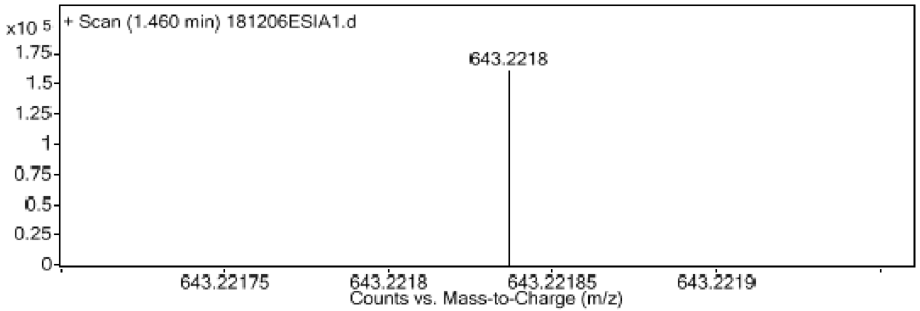


Peak List

| *m/z* | z | Abund | Formula | Ion |
|---|---|---|---|---|
| 360.3246 | 1 | 52920.93 | | |
| 437.1966 | 1 | 45872.7 | | |
| 441.2995 | 1 | 186783.45 | | |
| 442.3021 | 1 | 48050.51 | | |
| 478.323 | 1 | 30540.23 | | |
| 482.3256 | 1 | 57105.06 | | |
| 643.2218 | 1 | 161079.42 | C27 H40 Na O16 | M+ |
| 644.2253 | 1 | 44811.71 | C27 H40 Na O16 | M+ |
| 685.4361 | 1 | 58618.91 | | |
| 922.0098 | 1 | 80255.88 | | |

Formula Calculator Element Limits

| Element | Min | Max |
|---|---|---|
| C | 0 | 200 |
| H | 0 | 400 |
| O | 10 | 20 |
| Na | 1 | 1 |

Formula Calculator Results

| Formula | CalculatedMass | Mz | Diff.(mDa) | Diff. (ppm) | DBE |
|---|---|---|---|---|---|
| C27 H40 Na O16 | 643.2214 | 643.2218 | -0.4 | 0.6 | 7.5 |

--- End Of Report ---

**Fig. S63** HRESIMS spectrum of compound **7**

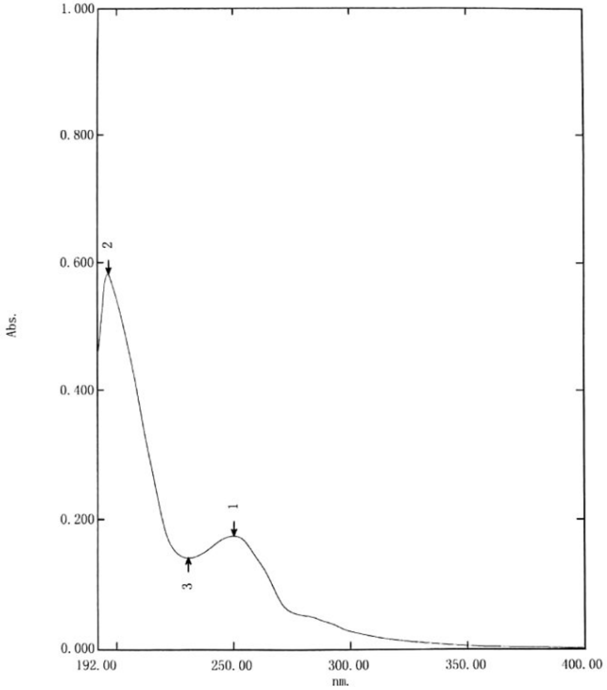


[测定属性]
波长范围(nm):　192.00 到 400.00
扫描速度:　中速
采样间隔:　0.2
自动采样间隔:　启用
扫描模式:　单个

[仪器属性]
仪器类型:　UV-2700 系列
测定方式:　吸收值
狭缝宽:　5.0 nm
积分时间:　0.1 秒
光源转换波长:　323.0 nm
检测器单元:　直接
S/R 转换:　标准
阶梯校正:　OFF

[附件属性]
附件:　无

[数据处理参数]
阈值:　0.0010000
点:　4
内插:　停用
平均:　停用

[样品准备属性]
重量:　1mg
体积:　1ml
稀释:　30倍
光程长:　10 mm
附加信息:　20181214 09:39:50
ped85 CH3OH

| No. | P/V | 波长(nm) | Abs. | 描述 |
|---|---|---|---|---|
| 1 | | 249.80 | 0.174 | |
| 2 | | 196.60 | 0.582 | |
| 3 | | 230.80 | 0.140 | |

**Fig. S64** UV spectrum of compound **7**

## Rudolph Research Analytical

This sample was measured on an Autopol VI, Serial #91058
Manufactured by Rudolph Research Analytical, Hackettstown, NJ, USA.

Measurement Date : Friday, 14-DEC-2018

Set Temperature : OFF

Time Delay : Disabled

Delay between Measurement : Disabled

| n | Average | Std.Dev. | % RSD | Maximum | Minimum |
|---|---|---|---|---|---|
| 5 | -51.36 | 0.40 | -0.77 | -51.10 | -52.00 |

| S.No | Sample ID | Time | Result | Scale | OR °Arc | WLG.nm | Lg.mm | Conc.g/100ml | Temp. |
|---|---|---|---|---|---|---|---|---|---|
| 1 | ped85 | 09:39:04 AM | -51.10 | SR | -0.0511 | 589 | 100.00 | 0.100 | 19.4 |
| 2 | ped85 | 09:39:12 AM | -51.10 | SR | -0.0511 | 589 | 100.00 | 0.100 | 19.4 |
| 3 | ped85 | 09:39:20 AM | -51.10 | SR | -0.0511 | 589 | 100.00 | 0.100 | 19.4 |
| 4 | ped85 | 09:39:28 AM | -51.50 | SR | -0.0515 | 589 | 100.00 | 0.100 | 19.4 |
| 5 | ped85 | 09:39:36 AM | -52.00 | SR | -0.0520 | 589 | 100.00 | 0.100 | 19.4 |

**Fig. S65** OR spectrum of compound **7**

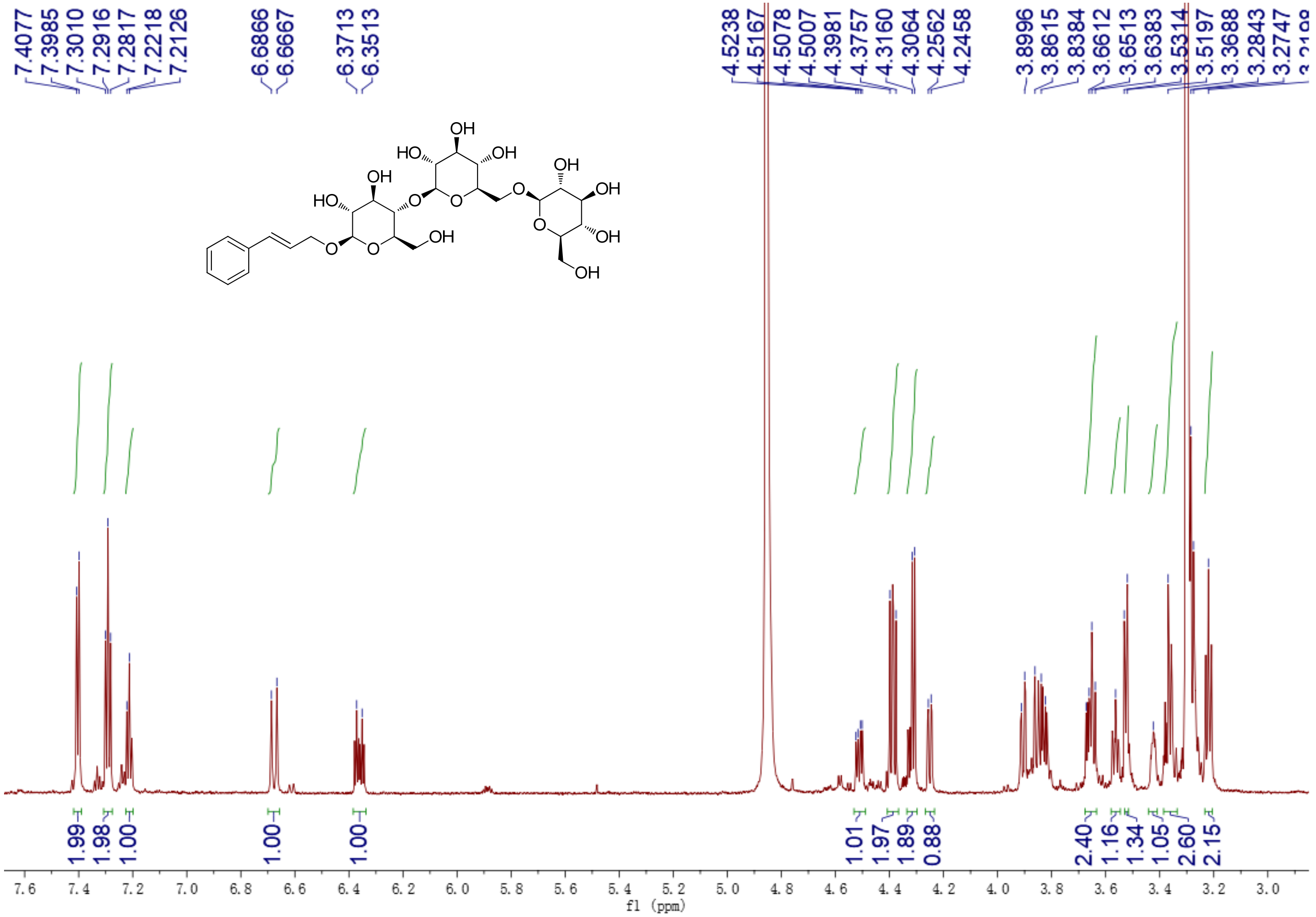


**Fig. S66** $^{1}$H NMR spectrum of compound **8** (methanol-$d_4$, 800 MHz)

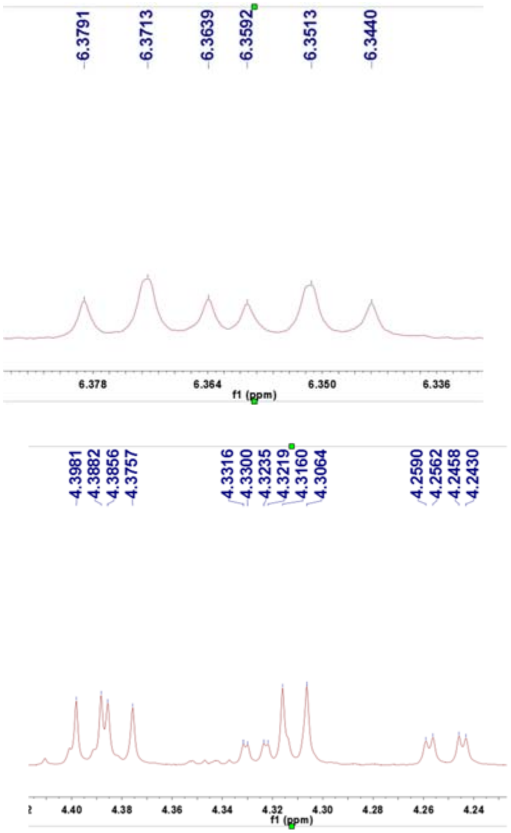

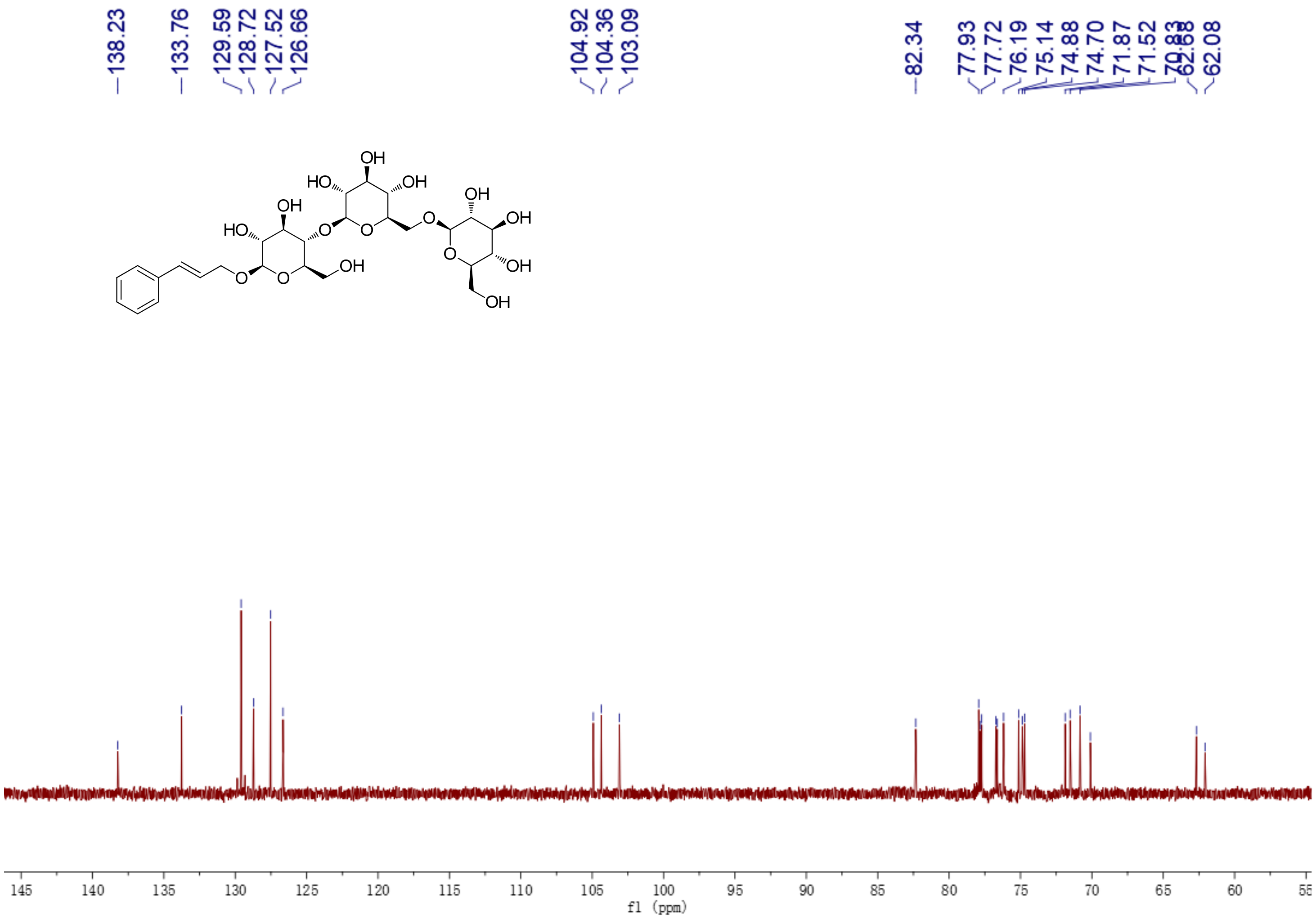


**Fig. S67** $^{13}$C NMR spectrum of compound **8** (methanol-$d_4$, 201 MHz)

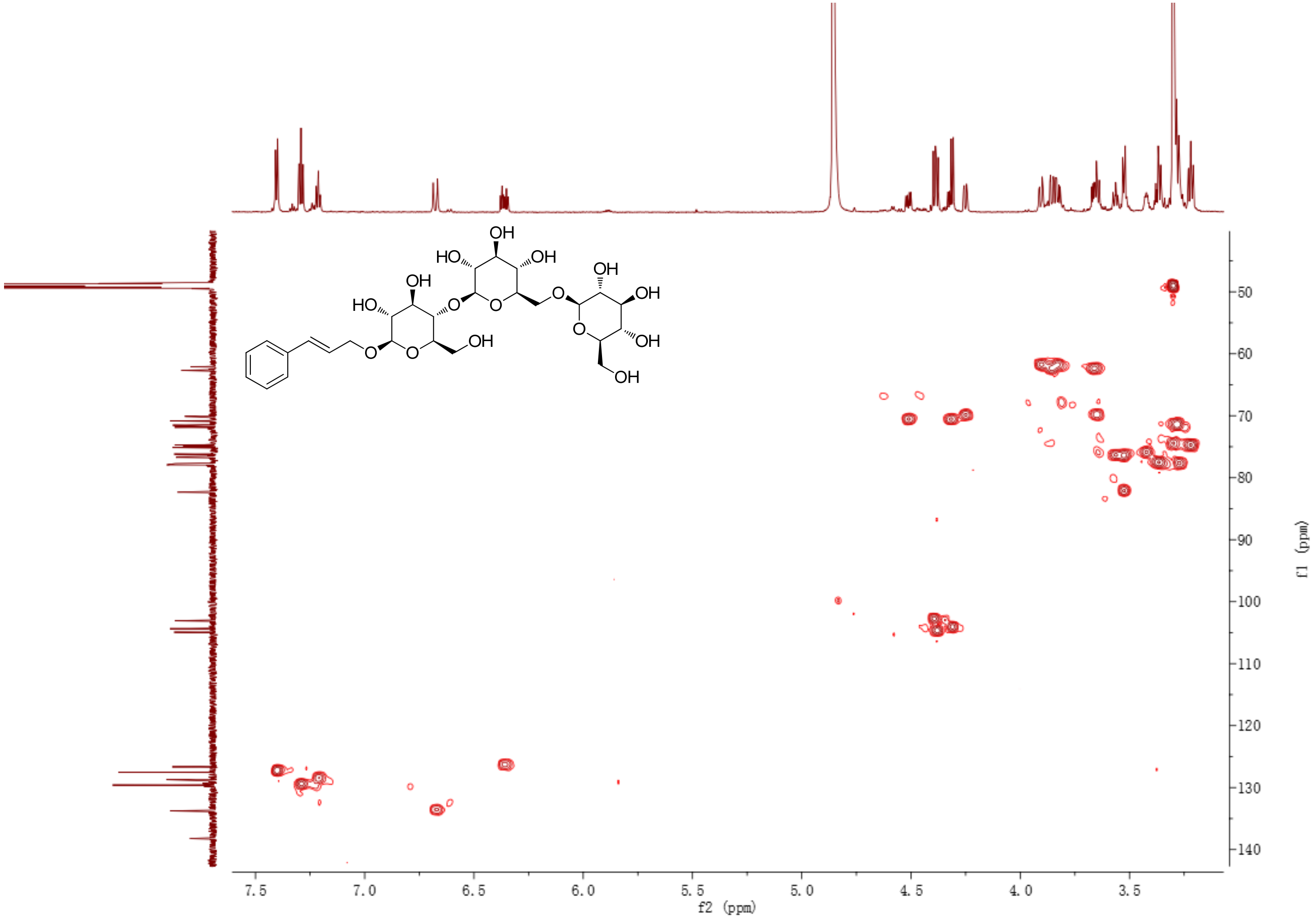


**Fig. S68** HSQC spectrum of compound **8**

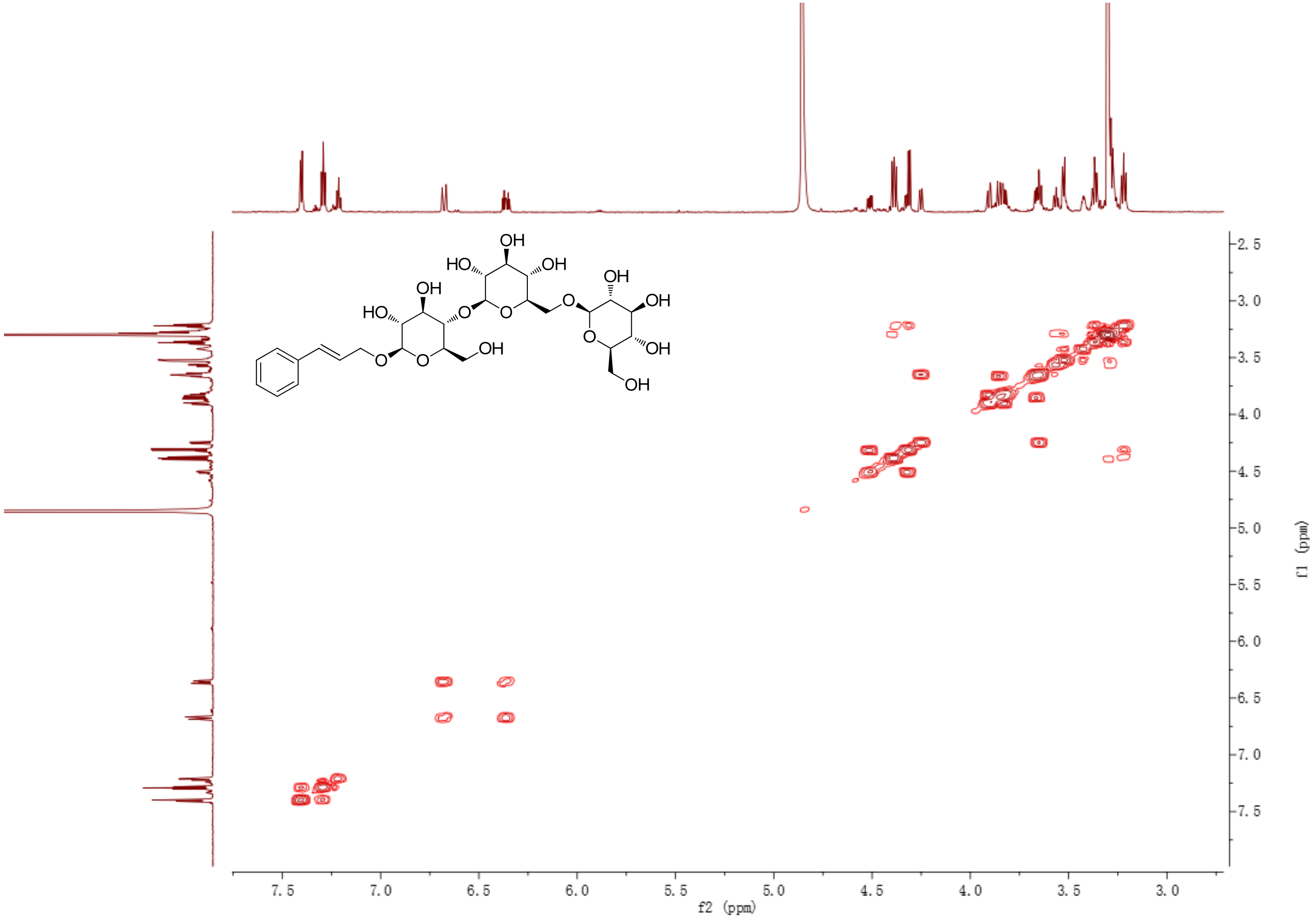


**Fig. S69** $^{1}H$–$^{1}H$ COSY spectrum of compound **8**

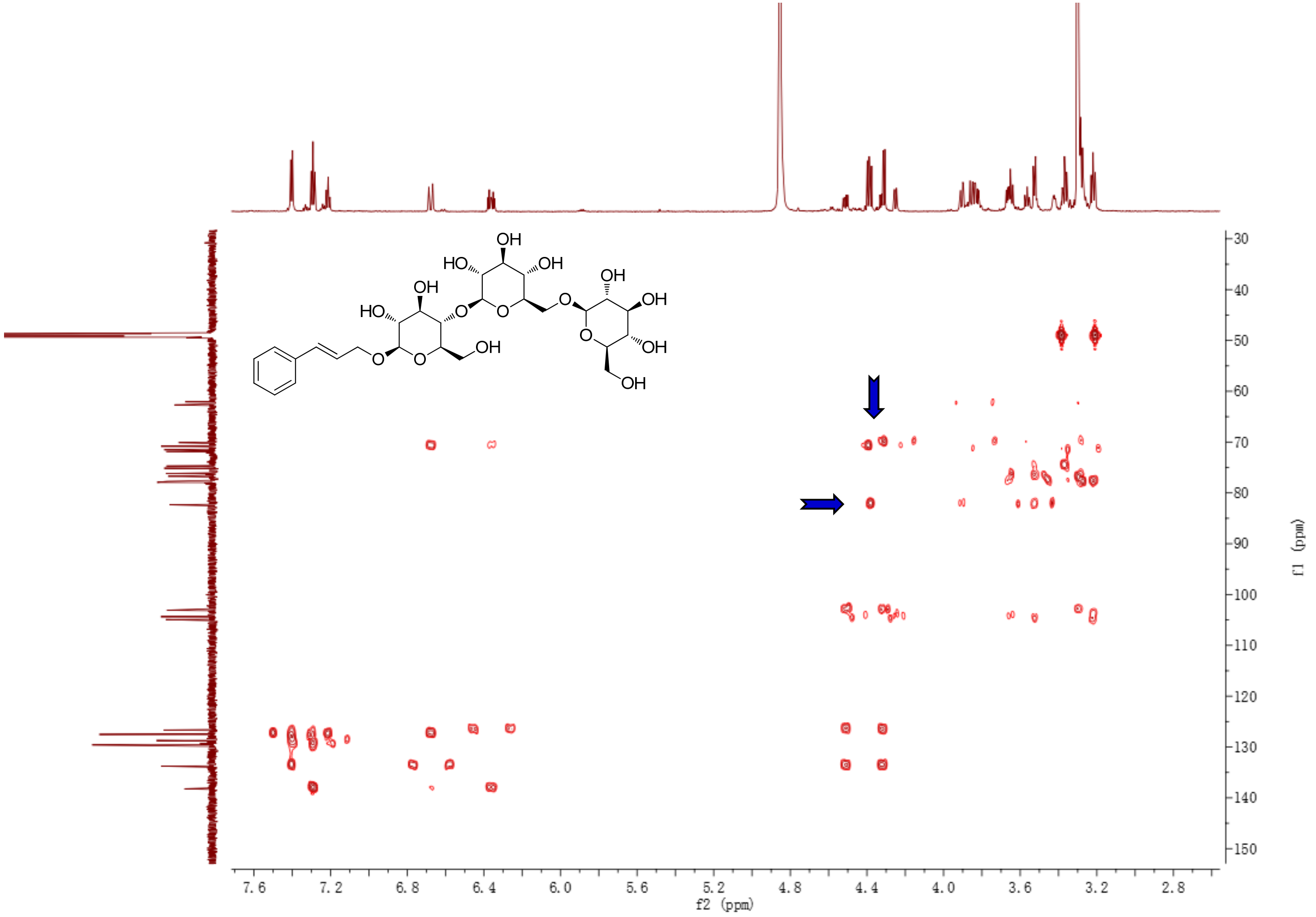


**Fig. S70** HMBC spectrum of compound **8**

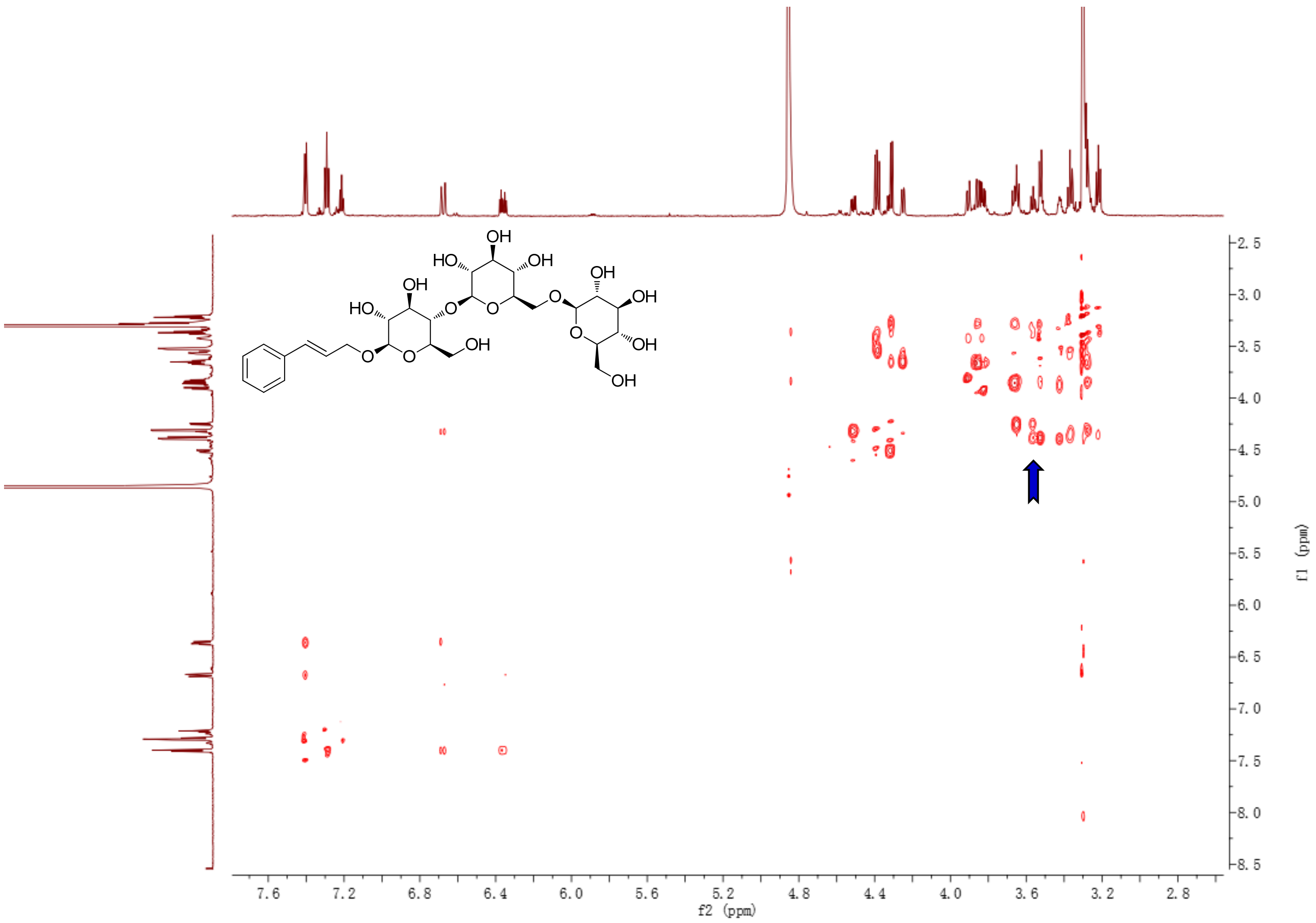


**Fig. S71** ROESY spectrum of compound **8**

## User Spectra

| Fragmentor Voltage | Collision Energy | Ionization Mode |
|---|---|---|
| 300 | 0 | ESI |

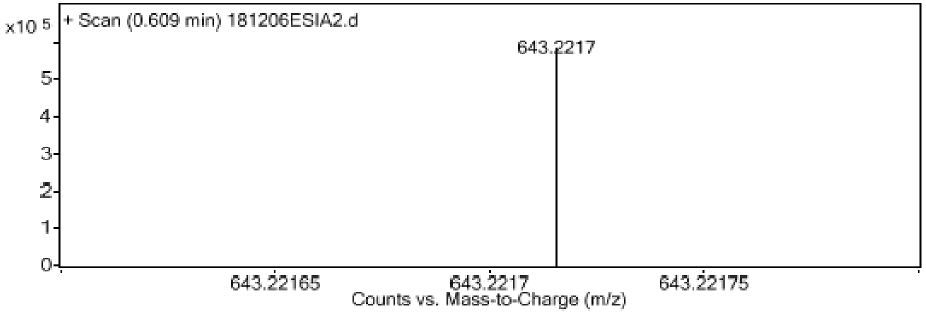


**Peak List**

| *m/z* | z | Abund | Formula | Ion |
|---|---|---|---|---|
| 105.044 | | 16083.98 | | |
| 627.2265 | 1 | 18620.26 | | |
| 643.2217 | 1 | 582532.56 | C27 H40 Na O16 | M+ |
| 644.2249 | 1 | 166235.64 | C27 H40 Na O16 | M+ |
| 645.2267 | 1 | 37175.2 | C27 H40 Na O16 | M+ |
| 659.1941 | 1 | 37907.19 | | |
| 665.2028 | 1 | 31903.84 | | |
| 922.0098 | 1 | 62282.75 | | |
| 1263.4492 | 1 | 24976.29 | | |
| 1264.4528 | 1 | 14969.83 | | |

**Formula Calculator Element Limits**

| Element | Min | Max |
|---|---|---|
| C | 0 | 200 |
| H | 0 | 400 |
| O | 10 | 20 |
| Na | 1 | 1 |

**Formula Calculator Results**

| Formula | CalculatedMass | Mz | Diff.(mDa) | Diff. (ppm) | DBE |
|---|---|---|---|---|---|
| C27 H40 Na O16 | 643.2214 | 643.2217 | -0.3 | 0.5 | 7.5 |

--- End Of Report ---

**Fig. S72** HRESIMS spectrum of compound **8**

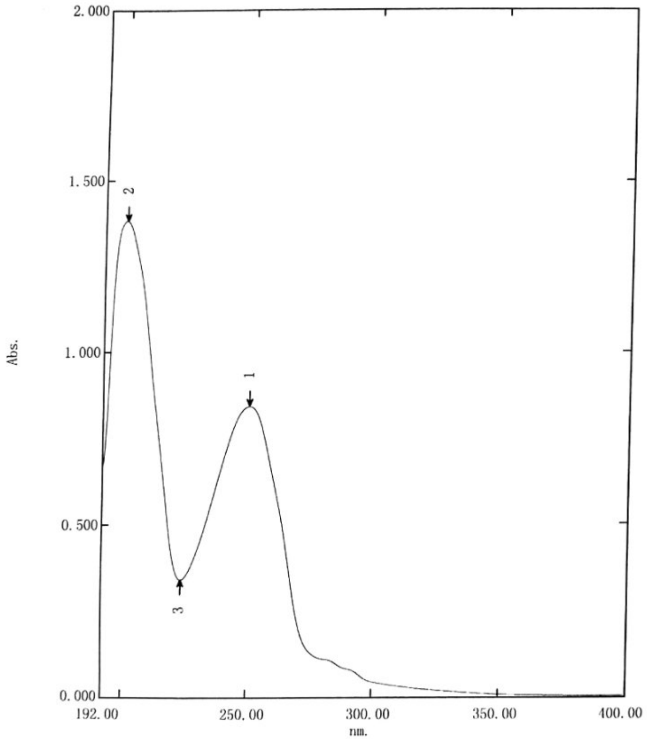


[测定属性]
波长范围(nm): 192.00 到 400.00
扫描速度: 中速
采样间隔: 0.2
自动采样间隔: 启用
扫描模式: 单个

[仪器属性]
仪器类型: UV-2700 系列
测定方式: 吸收值
狭缝宽: 5.0 nm
积分时间: 0.1 秒
光源转换波长: 323.0 nm
检测器单元: 直接
S/R 转换: 标准
阶梯校正: OFF

[附件属性]
附件: 无

[数据处理参数]
阈值: 0.0010000
点: 4
内插: 停用
平均: 停用

[样品准备属性]
重量: 0.8mg
体积: 1ml
稀释: 30倍
光程长: 10 mm
附加信息: 2018/12/20 10:22:42
ped87 CH3OH

| No. | P/V | 波长(nm) | Abs. | 描述 |
|---|---|---|---|---|
| 1 | | 249.40 | 0.844 | |
| 2 | | 200.40 | 1.381 | |
| 3 | | 223.80 | 0.340 | |

**Fig. S73** UV spectrum of compound **8**

## Rudolph Research Analytical

This sample was measured on an Autopol VI, Serial #91058
Manufactured by Rudolph Research Analytical, Hackettstown, NJ, USA.

Measurement Date : Thursday, 20-DEC-2018

Set Temperature : OFF

Time Delay : Disabled

Delay between Measurement : Disabled

| n | Average | Std.Dev. | % RSD | Maximum | Minimum |
|---|---|---|---|---|---|
| 5 | -68.48 | 0.65 | -0.94 | -67.50 | -69.25 |

| S.No | Sample ID | Time | Result | Scale | OR °Arc | WLG.nm | Lg.mm | Conc.g/100ml | Temp. |
|---|---|---|---|---|---|---|---|---|---|
| 1 | ped87 | 10:49:05 AM | -67.50 | SR | -0.0540 | 589 | 100.00 | 0.080 | 19.5 |
| 2 | ped87 | 10:49:13 AM | -68.25 | SR | -0.0546 | 589 | 100.00 | 0.080 | 19.5 |
| 3 | ped87 | 10:49:21 AM | -68.63 | SR | -0.0549 | 589 | 100.00 | 0.080 | 19.5 |
| 4 | ped87 | 10:49:29 AM | -68.75 | SR | -0.0550 | 589 | 100.00 | 0.080 | 19.5 |
| 5 | ped87 | 10:49:37 AM | -69.25 | SR | -0.0554 | 589 | 100.00 | 0.080 | 19.6 |

**Fig. S74** OR spectrum of compound **8**
